# Evaluating the performance of wearable EEG sleep monitoring devices: a meta-analysis approach

**DOI:** 10.1038/s44385-025-00034-w

**Published:** 2025-10-09

**Authors:** Karmen Markov, Mohamed Elgendi, Carlo Menon

**Affiliations:** 1 Biomedical and Mobile Health Technology Lab, Department of Health Sciences and Technology, ETH Zurich, Zurich, Switzerland; 2 Department of Biomedical Engineering and Biotechnology, Khalifa University of Science and Technology, Abu Dhabi, UAE; 3 Centre for Biotechnology (BTC), Khalifa University of Science and Technology, Abu Dhabi, UAE

**Keywords:** Biomarkers, Medical research, Nephrology, Signs and symptoms

## Abstract

Wearable EEG sleep monitoring devices (wEEGs) are increasingly popular in both clinical and consumer applications. However, their performance compared to polysomnography (PSG), the gold standard, remains under study. This meta-analysis of 43 validation studies assessed wEEGs against PSG, analyzing the influence of study design and device characteristics. The results revealed moderate to substantial agreement between wEEGs and PSG, with performance varying across sleep stages. The N1 stage posed significant classification challenges, while N3 (Deep Sleep) was most reliably detected. Manually scored wEEG data outperformed automatic scoring for N1 detection, and a higher electrode count was associated with improved N3 classification. This study proposes a standardized framework with balanced metrics like MCC and *κ* to address stage-specific performance variabilities, enhancing device comparability. The findings highlight the strengths and weaknesses of wEEGs and guide future research to refine automatic staging, contributing to their optimization for clinical and consumer applications.

## Introduction

The accurate detection of sleep stages is essential for understanding, diagnosing, and treating sleep disorders. Alterations in sleep stages have been linked to conditions like obstructive sleep apnea (N1 changes)^1^, restless legs syndrome (decreased rapid eye movement (REM) and N2 stages)^2^, obstructive sleep apnea-hypopnea syndrome (N3 changes)^3^, and narcolepsy (disrupted N2/N3 to REM transition)^4^. Moreover, sleep disturbances are commonly associated with neurodegenerative diseases^5–10^, epilepsy^11^, chronic pain^12,13^, depression^14,15^ and anxiety^16^. These conditions exhibit a bidirectional relationship with sleep, suggesting that addressing sleep disturbances could potentially delay the onset or ameliorate the symptoms, thereby improving patients’ quality of life^15,17^. Beyond clinical disorders, a recent meta-analysis demonstrated that greater proportions of N2 and REM sleep are positively associated with cognitive performance in healthy older adults, reinforcing the broader importance of accurate sleep staging^18^.

The American Academy of Sleep Medicine (AASM)^19^ provides the most widely used framework for sleep staging, grouping sleep into non-REM (NREM), comprising N1, N2 and N3 stages, and REM sleep. In sleep staging, 30-second record segments, known as epochs, are classified based on the most dominant characteristics: alpha rhythms for Wake, theta waves for N1, K complexes and sleep spindles for N2, and delta waves for N3 or deep sleep. REM sleep is characterized by mixed frequency electroencephalography (EEG), rapid eye movements, and sawtooth waves^19^.

Polysomnography (PSG) is the gold standard for clinical sleep assessment and diagnostics, capturing EEG, electrooculogram (EOG), and electromyogram (EMG) activity, as well as breathing effort, airflow, pulse, and blood oxygen saturation. However, PSG studies are expensive, time-consuming, require professional supervision and manual labelling, and suffer from high inter-rater variability (82.6% average agreement)^20^. Recent advancements in automatic sleep staging algorithms, as reviewed by Gaiduk et al.^21^ have shown high agreement with expert scorers and are increasingly being developed for clinical integration. These tools can accelerate diagnosis, reduce manual workload, and enable responsive interventions, such as real-time monitoring in sleep labs. Automated scoring may also help mitigate inter-rater variability, contributing to more consistent and objective assessments. Despite these advances, PSG remains uncomfortable for many patients, often requiring overnight stays in unfamiliar environments. Moreover, one-night PSG studies may not accurately reflect a patient’s typical sleep patterns and fail to capture night-to-night variability^22,23^. Recent advancements in dry electrodes have facilitated the development of wearable EEG sleep monitoring devices (wEEGs)^24^. These devices, using electrodes placed on the forehead, ear, or neck to acquire EEG signals, can offer a practical alternative to traditional PSG. Suitable for home use, these devices enable continuous sleep monitoring without the need for professional supervision.

Despite the growing popularity of wEEGs, their performance in accurately classifying sleep stages remains a subject of ongoing research. Prior systematic reviews have identified EEG as the most accurate sensing modality for detecting all sleep stages, whereas PPG-based systems, while more user-friendly, are limited in their staging accuracy^25^. A recent meta-analysis of EEG-based wearable devices has further highlighted the growing number of validated systems available, as well as considerable variability in their measurement properties, including accuracy, target population, and device features^26^. Nevertheless, the lack of standardized evaluation frameworks and inconsistent reporting of stage-specific performance metrics continue to hinder direct comparison between studies and devices. While overall per-epoch accuracy (ACC) is commonly reported, it may not adequately reflect the true performance of wEEGs due to the inherent class imbalance across sleep stages.

This meta-analysis evaluates the overall and sleep-stage-specific performance of wEEGs compared to PSG. Furthermore, we propose the use of Matthews correlation coefficient (MCC) as an alternative balanced evaluation metric. By providing a comprehensive evaluation of the wEEGs’ performance across sleep stages, this meta-analysis contributes valuable insights into their strengths and areas for further development, ultimately contributing to the optimization of wEEGs for clinical and consumer applications.

## Results

This section presents the findings from 43 validation studies that compare the performance of wEEGs in sleep stage classification with reference methods. 32 studies^22,27–57^ classified five stages—Wake, N1, N2, N3, and REM (Table 1). Seven studies^58–64^ classified four stages—Wake, Light (combining N1 and N2), Deep, and REM Sleep (Table 2). The remaining four studies used a three-stage classification (Wake, NREM, REM)^65,66^ or non-standard staging systems—one classified Wake, N1, N2 & N3, and REM^67^, and another reported Wake, N1, N2, and N3 without REM^68^ (Table 3).

**Table 1:** Overview of validation studies classifying five stages

Study	Device	# of al.^a^	El. position	El. type	# of part.	Age	Part.	Env.^b^	Nights	Ref.	Epochs	Device scoring^c^		OA (mi)	OA (ma)	Wake	N1	N2	N3	REM
*Forehead*																				
*Headband*																				
Li et al. (2025)^27^	WPSG-I^27^	6	Fp1, Fp2, M1, M2, Chin 1 (EMG), Chin 2 (EMG)	Dry	20 (M: 13, F: 7)	56.2 ± 9.5	Healthy: 6, PD: 8, ALS: 5, nacrolepsy: 1	Controlled	1	PSG scored by 2 experts according to AASM	26,572	WPSG-I proprietary algorithm	ACC	0.89	0.96	0.96	0.93	0.93	0.98	0.98
													*κ*	0.80	0.71	0.91	0.32	0.79	0.82	0.70
													SE	0.89	0.74	0.97	0.34	0.83	0.93	0.63
													SP	0.97	0.97	0.93	0.97	0.96	0.98	0.99
													PPV	0.89	0.75	0.96	0.36	0.85	0.75	0.81
													NPV	0.97	0.97	0.95	0.96	0.95	1.00	0.98
													F1	0.89	0.74	0.97	0.35	0.84	0.83	0.71
													MCC	0.80	0.71	0.91	0.32	0.79	0.82	0.70
												Manual scoring	ACC	0.95	0.98	0.98	0.96	0.97	0.99	0.99
													*κ*	0.90	0.86	0.95	0.65	0.91	0.91	0.89
													SE	0.95	0.89	0.98	0.70	0.91	0.98	0.89
													SP	0.99	0.98	0.97	0.98	0.99	0.99	1.00
													PPV	0.95	0.86	0.98	0.63	0.95	0.86	0.89
													NPV	0.99	0.98	0.97	0.98	0.97	1.00	1.00
													F1	0.95	0.88	0.98	0.66	0.93	0.91	0.89
													MCC	0.90	0.86	0.95	0.65	0.91	0.91	0.89
Ravindran et al. (2025)^28^	Dreem^69^	5	F7, F8, O1, O2, Fpz	Dry	62 (M: 35, F: 27)	70.5 ± 6.7 (44–83)	Healthy: 50, AD: 12	Controlled	1	PSG scored by 2 experts according to AASM	63,546	Dreem proprietary algorithm	ACC	0.69	0.88	0.89	0.88	0.77	0.92	0.93
													*κ*	0.58	0.54	0.74	0.14	0.52	0.68	0.61
													SE	0.69	0.61	0.74	0.14	0.86	0.65	0.68
													SP	0.92	0.92	0.96	0.97	0.73	0.97	0.95
													PPV	0.69	0.65	0.89	0.34	0.59	0.83	0.62
													NPV	0.92	0.92	0.89	0.90	0.92	0.93	0.96
													F1	0.69	0.62	0.81	0.20	0.70	0.73	0.65
													MCC	0.59	0.55	0.74	0.16	0.54	0.69	0.61
Seol et al. (2024)^29^	Insomnograf K2^73^	4	Fp1, Fp2, M1, M2 (Fpz as ref)	Wet	77 (N/R)	>20 years	Suspected or known OSA	Controlled	1	PSG scored by an expert according to AASM	75,677	Manual scoring	ACC	0.78	0.91	0.96	0.84	0.85	0.96	0.96
													*κ*	0.71	0.73	0.86	0.55	0.69	0.71	0.81
													SE	0.78	0.78	0.92	0.56	0.90	0.67	0.85
													SP	0.95	0.94	0.97	0.94	0.82	0.99	0.97
													PPV	0.78	0.80	0.85	0.78	0.75	0.81	0.83
													NPV	0.95	0.94	0.99	0.85	0.93	0.97	0.98
													F1	0.78	0.78	0.88	0.65	0.82	0.73	0.84
													MCC	0.71	0.73	0.86	0.56	0.70	0.71	0.81
Rusanen et al. (2023)^30^	Focusband^78^	2	Fp1, Fp2 (Fpz as ref)	Dry	10 (M: 7, F: 3)	23–37	Healthy	Home (devices fitted by a specialist)	1	PSG scored by an expert according to AASM	9337	Deep learning (CNN)	ACC	0.82	0.93	0.94	0.98	0.85	0.93	0.94
													*κ*	0.75	0.67	0.78	0.26	0.69	0.79	0.67
													SE	0.82	0.70	0.80	0.21	0.87	0.77	0.86
													SP	0.96	0.95	0.97	0.99	0.83	0.98	0.96
													PPV	0.82	0.74	0.83	0.35	0.79	0.89	0.85
													NPV	0.96	0.95	0.96	0.98	0.90	0.94	0.97
													F1	0.82	0.72	0.82	0.28	0.82	0.83	0.85
													MCC	0.75	0.67	0.78	0.28	0.69	0.79	0.82
Casciola et al. (2021)^31^	Cognionics^79^	4	F3, F4, A1, A2	Dry	12 (M: 6, F: 6)	21–61	Healthy	Controlled	1	PSG scored by an expert according to AASM	9747	Deep learning (CNN + LSTM)	ACC	0.74	0.90	0.92	0.89	0.83	0.95	0.90
													*κ*	0.64	0.59	0.76	0.20	0.65	0.76	0.57
													SE	0.74	0.69	0.82	0.28	0.74	0.87	0.73
													SP	0.93	0.93	0.94	0.93	0.91	0.96	0.92
													PPV	0.74	0.64	0.81	0.24	0.88	0.73	0.55
													NPV	0.93	0.93	0.95	0.95	0.79	0.98	0.96
													F1	0.74	0.66	0.81	0.26	0.80	0.79	0.63
													MCC	0.64	0.59	0.76	0.20	0.66	0.77	0.58
												Machine learning (Ensemble-bagged trees model)	ACC	0.68^d^	0.49^d^	N/R	N/R	N/R	N/R	N/R
													SE	N/R	N/R	0.80^d^	0.04^d^	0.82^d^	0.50^d^	0.28^d^
Arnal et al. (2020)^32^	Dreem^69^	4	F7, F8, O1, O2, Fpz	Dry	25 (M: 19, F: 6)	35.3 ± 7.5 (23–50)	Mostly healthy, some with mild symptoms of anxiety or depression, one with insomnia	Controlled	1	PSG scored by 5 experts according to AASM	24,662	Deep learning (2 layers of LSTM + Softmax function)	ACC	0.81	0.92	0.95	0.93	0.86	0.95	0.93
													*κ*	0.73	0.69	0.76	0.43	0.72	0.78	0.78
													SE	0.81	0.76	0.78	0.48	0.83	0.87	0.86
													SP	0.95	0.95	0.97	0.96	0.90	0.96	0.95
													PPV	0.81	0.74	0.80	0.46	0.89	0.76	0.79
													NPV	0.95	0.94	0.97	0.96	0.84	0.98	0.97
													F1	0.81	0.75	0.79	0.47	0.86	0.81	0.83
													MCC	0.73	0.70	0.76	0.43	0.72	0.78	0.78
Lin et al. (2017)^33^	Prototype developed in the study^33^	4	AF7, Fp1, Fp2, AF8	Dry	10 (M: 10, F: 0)	24 ± 6	Healthy	Controlled	1	PSG scored by an expert according to AASM	8251	Machine learning (RVM)	ACC	0.77	0.91	0.94	0.86	0.86	0.95	0.92
													*κ*	0.69	0.65	0.80	0.23	0.70	0.83	0.72
													SE	0.77	0.72	0.84	0.22	0.85	0.87	0.83
													SP	0.94	0.94	0.96	0.96	0.86	0.97	0.94
													PPV	0.77	0.72	0.83	0.43	0.79	0.84	0.71
													NPV	0.94	0.94	0.96	0.89	0.90	0.98	0.97
													F1	0.77	0.71	0.83	0.29	0.82	0.86	0.77
													MCC	0.69	0.66	0.80	0.24	0.70	0.83	0.73
Levendowski et al. (2017)^22^	X4 Sleep Profiler^70^	2	AF7, AF8 (Fpz as ref)	Dry	47 (M: 35, F:12)	23–77	Sleep-disordered breathing and healthy	Controlled	1	PSG scored by 5 experts according to AASM	33,635	X4 Sleep Profiler proprietary algorithm	ACC	0.77	0.91	0.90	0.87	0.86	0.96	0.95
													*κ*	0.68	0.64	0.73	0.22	0.72	0.78	0.75
													SE	0.77	0.71	0.77	0.32	0.83	0.79	0.83
													SP	0.94	0.94	0.94	0.92	0.89	0.98	0.96
													PPV	0.77	0.70	0.82	0.26	0.85	0.82	0.73
													NPV	0.94	0.94	0.92	0.94	0.87	0.97	0.98
													F1	0.77	0.70	0.79	0.29	0.84	0.81	0.78
													MCC	0.68	0.64	0.73	0.22	0.72	0.78	0.75
												Automatic scoring corrected by reviewer	ACC	0.80	0.92	0.92	0.88	0.87	0.96	0.97
													*κ*	0.72	0.69	0.79	0.28	0.74	0.79	0.85
													SE	0.80	0.75	0.82	0.36	0.85	0.80	0.94
													SP	0.95	0.95	0.96	0.93	0.89	0.98	0.97
													PPV	0.80	0.74	0.87	0.33	0.86	0.83	0.80
													NPV	0.95	0.95	0.94	0.94	0.88	0.97	0.99
													F1	0.80	0.74	0.84	0.34	0.85	0.81	0.87
													MCC	0.72	0.69	0.79	0.28	0.74	0.79	0.85
Finan et al. (2016)^34^	X4 Sleep Profiler^70^	2	AF7, AF8 (Fpz as ref)	Dry	14 (M: 6, F: 8)	26.4 ± 3.7 (22–34)	Healthy	Controlled	1	PSG scored by an expert according to AASM	13,445*	X4 Sleep Profiler proprietary algorithm	ACC	0.66	0.86	0.92	0.89	0.78	0.88	0.85
													*κ*	0.52	0.45	0.42	0.07	0.55	0.62	0.58
													SE	0.66	0.55	0.44	0.27	0.72	0.60	0.72
													SP	0.92	0.91	0.96	0.91	0.83	0.96	0.89
													PPV	0.66	0.56	0.47	0.07	0.79	0.81	0.63
													NPV	0.92	0.91	0.96	0.98	0.77	0.89	0.92
													F1	0.66	0.54	0.46	0.11	0.75	0.69	0.67
													MCC	0.53	0.46	0.42	0.09	0.56	0.58	0.58
												Manual scoring	ACC	0.74	0.90	0.94	0.93	0.81	0.90	0.90
													*κ*	0.62	0.52	0.44	0.14	0.61	0.71	0.72
													SE	0.74	0.61	0.40	0.27	0.76	0.74	0.86
													SP	0.93	0.93	0.98	0.95	0.85	0.95	0.91
													PPV	0.74	0.61	0.57	0.12	0.81	0.80	0.72
													NPV	0.93	0.92	0.95	0.98	0.80	0.93	0.96
													F1	0.74	0.60	0.47	0.17	0.79	0.77	0.79
													MCC	0.62	0.53	0.45	0.15	0.61	0.71	0.73
*Sleepmask*																				
Liang et al. (2015)^35^	Prototype developed in the study^35^	2	EOG L, EOG R (Fpz as ref)	Dry	16 (M:11, F:5)	25.3 ± 2.5	Healthy	Controlled	1	PSG scored by an expert according to AASM	6480	Machine learning (LDA)	ACC	0.84	0.94	0.96	0.97	0.87	0.93	0.96
													*κ*	0.77	0.69	0.69	0.33	0.74	0.80	0.87
													SE	0.84	0.77	0.84	0.43	0.83	0.81	0.94
													SP	0.96	0.96	0.97	0.98	0.91	0.97	0.96
													PPV	0.84	0.71	0.62	0.29	0.89	0.87	0.87
													NPV	0.96	0.95	0.99	0.99	0.85	0.95	0.98
													F1	0.84	0.73	0.71	0.35	0.86	0.84	0.90
													MCC	0.77	0.69	0.70	0.34	0.74	0.80	0.88
*Sheet-like/patches*																				
Massie et al. (2025)^36^	Prototype developed in the study^36^	1	EOG R (Fpz as ref)	Dry	106 (M: 60, F: 46)	58 ± 15 (22–82)	Suspected OSA	Controlled	1	PSG scored by experts according to AASM	81,786	Deep learning (RNN)	ACC	0.80	0.92	0.92	0.95	0.84	0.94	0.95
													*κ*	0.70	0.66	0.77	0.44	0.69	0.62	0.78
													SE	0.80	0.74	0.84	0.47	0.82	0.76	0.80
													SP	0.95	0.94	0.94	0.97	0.88	0.95	0.98
													PPV	0.80	0.71	0.80	0.47	0.87	0.57	0.82
													NPV	0.95	0.94	0.95	0.97	0.82	0.98	0.97
													F1	0.80	0.72	0.82	0.47	0.84	0.65	0.81
													MCC	0.70	0.66	0.77	0.44	0.69	0.62	0.78
Roach et al. (2025)^37^	Somfit^80^	1	Fpz	Wet	27 (M: 13, F: 14)	22.3 ± 5.1	Healthy	Controlled	1	PSG scored by 3 experts according to AASM	21,600	Somfit proprietary algorithm	ACC	N/R	N/R	N/R	N/R	N/R	N/R	N/R
													*κ*	0.47^d^	N/R	N/R	N/R	N/R	N/R	N/R
													SE	N/R	N/R	0.60^d^	0.19^d^	0.69^d^	0.61^d^	0.53^d^
Um et al. (2025)^38^	Prototype developed in the study^38^	4	F7, F8, EOG L, EMG L, chin	Dry	1 (N/R)	N/R	Healthy	Controlled	1	PSG scored by an expert according to AASM	688	Deep learning (BiLSTM + attention model on spectrogram input)	ACC	0.73	0.89	0.96	0.78	0.79	0.98	0.95
													*κ*	0.61	0.65	0.74	0.42	0.59	0.66	0.83
													SE	0.73	0.75	0.72	0.73	0.70	0.77	0.83
													SP	0.93	0.93	0.98	0.79	0.90	0.99	0.98
													PPV	0.73	0.72	0.81	0.45	0.88	0.59	0.89
													NPV	0.93	0.92	0.98	0.93	0.74	0.99	0.96
													F1	0.73	0.72	0.76	0.56	0.78	0.67	0.86
													MCC	0.63	0.66	0.74	0.45	0.60	0.66	0.83
McMahon et al. (2024)^39^	Somfit^80^	1	Fpz	Wet	106 (M: 59, F: 47)	<65:85 ≥ 65:21	Suspected or known OSA	Controlled	1	PSG scored by 3 experts according to AASM	N/R	Deep learning (U-sleep CNN)	ACC	N/R	N/R	0.89^d^	0.91^d^	0.84^d^	0.94^d^	0.95^d^
													*κ*	0.67^d^	N/R	N/R	N/R	N/R	N/R	N/R
													SE	N/R	N/R	0.78^d^	0.22^d^	0.84^d^	0.58^d^	0.87^d^
													SP	N/R	N/R	0.91^d^	0.97^d^	0.83^d^	0.99^d^	0.96^d^
													PPV	N/R	N/R	0.76^d^	0.38^d^	0.76^d^	0.85^d^	0.76^d^
													NPV	N/R	N/R	0.93^d^	0.93^d^	0.90^d^	0.94^d^	0.98^d^
													F1	N/R	N/R	0.77^e^	0.28^e^	0.80^e^	0.69^e^	0.81^e^
Oz et al. (2023)^40^	X-trodes soft electrode array^81^	8	4 EEG (forehead), 2 EOG R, 2 EMG R (chin)	Dry	50 (M: 32, F: 18)	61.4 ± 7.9	Healthy: 21 PD: 29	Controlled	1	PSG scored by 2 experts according to the AASM	N/R	Manual scoring	ACC	0.77^d^	N/R	N/R	N/R	N/R	N/R	N/R
													*κ*	0.69^d^	N/R	0.70^d^	0.22^d^	0.58^d^	0.41^d^	0.72^d^
													SE	N/R	N/R	0.91^d^	0.16^d^	0.84^d^	0.68^d^	0.77^d^
													SP	N/R	N/R	0.94^d^	0.99	0.80^d^	0.97^d^	0.98^d^
													PPV	N/R	N/R	0.84^d^	0.44^d^	0.71^d^	0.83^d^	0.85^d^
													F1	N/R	N/R	0.87^e^	0.23^e^	0.77^e^	0.75^e^	0.81^e^
Kwon et al. (2023)^41^	Skin patch developed in the study^41^	5	2 EEG (forehead), EOG R, EOG L, EMG (chin)	Dry	8 (N/R)	N/R	Healthy	Controlled	1	PSG scored by an expert according to AASM	4961	Deep learning (CNN)	ACC	0.84	0.94	0.94	0.93	0.89	0.97	0.96
													*κ*	0.76	0.68	0.82	0.23	0.77	0.80	0.80
													SE	0.84	0.72	0.84	0.17	0.94	0.78	0.88
													SP	0.96	0.95	0.97	0.99	0.84	0.99	0.97
													PPV	0.84	0.78	0.88	0.52	0.85	0.86	0.77
													NPV	0.96	0.96	0.95	0.94	0.93	0.98	0.99
													F1	0.84	0.73	0.86	0.25	0.89	0.82	0.82
													MCC	0.76	0.69	0.82	0.27	0.78	0.81	0.80
												Manual scoring	ACC	0.82	0.93	0.92	0.90	0.88	0.96	0.98
													κ	0.74	0.69	0.76	0.23	0.76	0.78	0.92
													SE	0.82	0.73	0.72	0.30	0.94	0.75	0.92
													SP	0.96	0.95	0.98	0.94	0.83	0.99	0.99
													PPV	0.82	0.77	0.93	0.28	0.84	0.86	0.93
													NPV	0.96	0.95	0.92	0.95	0.93	0.97	0.99
													F1	0.82	0.74	0.81	0.29	0.89	0.80	0.93
													MCC	0.74	0.70	0.77	0.23	0.77	0.79	0.92
Matsumori et al. (2022)^42^	Prototype developed in the study^42^	6	Forehead (ref on mastoid)	Wet	27 (M:23, F:4)	27.4 ± 9.2	Healthy	Controlled	1	PSG scored by an expert according to the AASM	24,979	Deep learning (DSN: CNN + BiLSTM)	ACC	0.81	0.92	0.97	0.90	0.92	0.95	0.89
													*κ*	0.74	0.71	0.77	0.47	0.83	0.82	0.69
													SE	0.81	0.76	0.70	0.58	0.85	0.85	0.83
													SP	0.95	0.95	0.99	0.93	0.97	0.97	0.90
													PPV	0.81	0.77	0.88	0.48	0.95	0.86	0.70
													NPV	0.95	0.95	0.97	0.95	0.90	0.97	0.95
													F1	0.81	0.76	0.78	0.52	0.90	0.85	0.76
													MCC	0.74	0.72	0.77	0.47	0.83	0.82	0.69
Myllymaa et al. (2016)^43^	Bittium Brainstatus EEG^82^	11	Fp1, Fp2, AF7, AF8, F8, F7, Sp1, Sp2, T10, T9, EOG R	Wet	31 (M:10, F: 21)	31.3 ± 11.8	Sleep bruxism or healthy	Controlled	1	PSG scored by 2 experts according to the AASM	27,692	Manual scoring	ACC	0.80	0.92	0.94	0.89	0.86	0.94	0.96
													*κ*	0.71	0.69	0.76	0.43	0.72	0.79	0.77
													SE	0.80	0.74	0.76	0.57	0.87	0.77	0.75
													SP	0.95	0.94	0.97	0.93	0.85	0.98	0.98
													PPV	0.80	0.77	0.83	0.44	0.83	0.89	0.85
													NPV	0.95	0.94	0.96	0.96	0.89	0.95	0.97
													F1	0.80	0.75	0.79	0.49	0.85	0.83	0.79
													MCC	0.71	0.70	0.76	0.44	0.72	0.79	0.77
Ear																				
In-ear																				
Borges et al. (2025)^44^	Prototype developed in Goverdovsky et al. (2016^74^, 2017)^75^	4	2 per ear	Wet	14 (M: 6, F: 8)	53.2 ± 17.4 (25–78)	Mostly OSA	Controlled	1	PSG scored by an expert according to AASM	30,960	Automatic (not specified)	ACC	0.83	0.93	0.94	0.91	0.89	0.95	0.98
													*κ*	0.77	0.77	0.83	0.56	0.77	0.77	0.89
													SE	0.83	0.80	0.81	0.60	0.91	0.74	0.93
													SP	0.96	0.95	0.98	0.95	0.87	0.98	0.98
													PPV	0.83	0.83	0.93	0.62	0.82	0.87	0.88
													NPV	0.96	0.96	0.95	0.94	0.94	0.96	0.99
													F1	0.83	0.81	0.87	0.61	0.86	0.80	0.91
													MCC	0.77	0.77	0.83	0.56	0.77	0.78	0.89
Hammour et al. (2024)^45^	Prototype developed in Goverdovsky et al. (2016)^74^, (2017)^75^	4	2 per ear	Wet	13 (M: 9, F: 4)	71.8 ± 4.4 (65–83)	Mostly healthy; some with stable comorbidities (type-2 diabetes, sleep apnea, hypertension)	Controlled	1	PSG scored by 2 scorers according to AASM	13,403	Machine learning (fine-tuned pre-trained LightGBM^83^)	ACC	0.74	0.90	0.90	0.86	0.83	0.94	0.95
													*κ*	0.64	0.62	0.79	0.29	0.59	0.73	0.69
													SE	0.74	0.67	0.90	0.36	0.72	0.74	0.64
													SP	0.93	0.93	0.90	0.92	0.87	0.97	0.98
													PPV	0.74	0.71	0.84	0.38	0.70	0.79	0.81
													NPV	0.93	0.93	0.94	0.91	0.88	0.96	0.96
													F1	0.74	0.69	0.87	0.37	0.71	0.77	0.72
													MCC	0.64	0.62	0.79	0.29	0.59	0.73	0.70
Borup et al. (2023)^46^	Prototype developed in Kappel et al. (2018)^72^	12	6 per ear	Dry	20 (M: 7, F:13)	25.9 (22–36)	Healthy	Home (devices fitted by a specialist)	4	PSG scored by an expert according to AASM	72,942	Deep learning (personalized ensemble deep learning)	ACC	0.84	0.94	0.98	0.94	0.88	0.95	0.94
													*κ*	0.78	0.75	0.88	0.47	0.76	0.85	0.80
													SE	0.84	0.79	0.92	0.44	0.89	0.83	0.85
													SP	0.96	0.96	0.98	0.98	0.87	0.99	0.96
													PPV	0.84	0.81	0.88	0.57	0.83	0.95	0.82
													NPV	0.96	0.96	0.99	0.96	0.92	0.95	0.97
													F1	0.84	0.80	0.90	0.50	0.86	0.88	0.84
													MCC	0.78	0.75	0.88	0.47	0.76	0.86	0.80
Tabar et al. (2023)^47^	Prototype developed in the study^47^	4	2 per ear	Dry	10 (M: 6, F: 4)	27.4 ± 4.9 (22–35)	Healthy	Home (device fitted by participants, PSG by specialist)	2	Partial PSG scored by an expert according to AASM	15,709	Machine learning (Random Forest)	ACC	0.81	0.92	0.96	0.92	0.85	0.96	0.93
													*κ*	0.72	0.65	0.70	0.23	0.70	0.86	0.77
													SE	0.81	0.71	0.76	0.17	0.89	0.90	0.81
													SP	0.95	0.94	0.97	0.99	0.81	0.97	0.96
													PPV	0.81	0.75	0.70	0.55	0.81	0.87	0.82
													NPV	0.95	0.95	0.98	0.93	0.89	0.98	0.95
													F1	0.81	0.71	0.73	0.25	0.85	0.89	0.81
													MCC	0.72	0.66	0.70	0.27	0.71	0.86	0.77
Jørgensen et al. (2023)^48^	Prototype developed in Kappel et al. (2018)^72^	12	6 per ear	Dry	1 (M: 0, F:1)	29 ± 3.8 (22–35)	Healthy	Home (devices fitted by a specialist)	2	PSG scored by an expert according to AASM	1578	Machine learning (Random Forest)	ACC	0.79	0.92	0.98	0.91	0.82	0.93	0.95
													*κ*	0.72	0.67	0.83	0.24	0.60	0.81	0.88
													SE	0.79	0.71	0.90	0.48	0.58	1.00	0.96
													SP	0.95	0.95	0.99	0.93	0.98	0.91	0.95
													PPV	0.79	0.78	0.78	0.20	0.94	0.75	0.89
													NPV	0.95	0.95	1.00	0.98	0.77	1.00	0.98
													F1	0.79	0.72	0.84	0.28	0.72	0.86	0.92
													MCC	0.73	0.69	0.83	0.27	0.63	0.83	0.89
Kjaer et al. (2022)^49^	Prototype developed in Kappel et al. (2018)^72^	12	6 per ear	Dry	20 (M: 7, F: 13)	25.9 ± 3.8 (22–36)	Healthy	Home (devices fitted by a specialist)	4	PSG scored by 2 experts according to AASM	72,942	Machine learning (Random Forest)	ACC	0.80	0.92	0.96	0.94	0.84	0.96	0.91
													*κ*	0.73	0.65	0.84	0.15	0.68	0.85	0.70
													SE	0.80	0.70	0.91	0.10	0.87	0.85	0.75
													SP	0.95	0.94	0.97	1.00	0.82	0.98	0.95
													PPV	0.80	0.76	0.82	0.54	0.78	0.92	0.76
													NPV	0.95	0.95	0.98	0.95	0.90	0.96	0.94
													F1	0.80	0.70	0.87	0.16	0.82	0.88	0.76
													MCC	0.73	0.66	0.84	0.21	0.68	0.86	0.70
Jørgensen et al. (2020)^50^	Prototype developed in Zibrandtsen et al. (2016)^84^	8	4 per ear	Wet	13 (M: 5, F: 8)	41.5 (18–60)	Epilepsy	Controlled	1–4	PSG scored by an expert according to AASM	27,593	Manual scoring	ACC	0.81	0.92	0.96	0.89	0.85	0.94	0.98
													*κ*	0.74	0.74	0.85	0.47	0.70	0.79	0.90
													SE	0.81	0.80	0.91	0.63	0.80	0.80	0.87
													SP	0.95	0.95	0.97	0.92	0.89	0.97	0.99
													PPV	0.81	0.79	0.84	0.46	0.86	0.86	0.96
													NPV	0.95	0.95	0.98	0.96	0.85	0.96	0.98
													F1	0.81	0.79	0.87	0.53	0.83	0.83	0.91
													MCC	0.74	0.74	0.85	0.48	0.70	0.80	0.90
Nakamura et al. (2020)^51^	Prototype developed in Goverdovsky et al. (2016)^74^, (2017)^75^	4	2 per ear	Wet	22 (N/R)	23.8 ± 4.8	Healthy	Home (devices fitted by specialist)	1	PSG scored by an expert according to AASM	11,610	Machine learning (SVM)	ACC	0.74	0.90	0.89	0.99	0.77	0.93	0.90
													*κ*	0.61	0.51	0.69	0.09	0.55	0.75	0.49
													SE	0.74	0.57	0.74	0.05	0.84	0.75	0.46
													SP	0.94	0.92	0.94	1.00	0.71	0.97	0.97
													PPV	0.74	0.73	0.77	0.64	0.71	0.84	0.67
													NPV	0.94	0.93	0.93	0.99	0.84	0.95	0.92
													F1	0.74	0.59	0.76	0.09	0.77	0.79	0.54
													MCC	0.62	0.53	0.69	0.18	0.56	0.75	0.50
Mikkelsen et al. (2019)^52^	Prototype developed in Kappel et al. (2018)^72^	12	6 per ear	Dry	20 (M:7, F:13)	25.9 (22–36)	Healthy	Home (devices fitted by a specialist)	4	Partial PSG scored by an expert according to AASM	72,942	Machine learning (Random Forest)	ACC	0.81	0.92	0.96	0.93	0.85	0.96	0.91
													*κ*	0.73	0.68	0.85	0.29	0.69	0.86	0.70
													SE	0.81	0.77	0.84	0.52	0.79	0.93	0.77
													SP	0.95	0.95	0.98	0.94	0.90	0.97	0.94
													PPV	0.81	0.72	0.91	0.23	0.88	0.85	0.74
													NPV	0.95	0.94	0.97	0.98	0.82	0.99	0.95
													F1	0.81	0.73	0.87	0.32	0.83	0.89	0.76
													MCC	0.73	0.68	0.85	0.31	0.69	0.86	0.70
Mikkelsen et al. (2017)^53^	Prototype developed in the study^53^	12	6 per ear	Wet	9 (M: 6, F: 3)	26–44	Healthy	Home (devices fitted by a specialist)	1	Partial PSG scored by experts according to AASM	7411	Machine learning (Random Forest)	ACC	0.60	0.84	0.84	0.92	0.74	0.91	0.80
													*κ*	0.45	0.40	0.52	0.04	0.47	0.61	0.34
													SE	0.60	0.52	0.53	0.16	0.69	0.74	0.46
													SP	0.90	0.89	0.94	0.93	0.78	0.94	0.88
													PPV	0.60	0.51	0.74	0.04	0.71	0.59	0.47
													NPV	0.90	0.89	0.86	0.98	0.77	0.97	0.87
													F1	0.60	0.50	0.61	0.07	0.70	0.66	0.47
													MCC	0.45	0.40	0.53	0.05	0.47	0.61	0.34
*Around-the-ear*																				
da Silva Suoto et al. (2022)^55^	Prototype developed in the study^55^	7	2 EMG, 1 EOG, 2 forehead, 2 around-the-ear	Wet	12 (M: 9, F: 3)	28.9 (18–45)	Healthy	Home (devices fitted by a specialist)	1	PSG scored by an expert according to AASM	10,632	Manual scoring	ACC	0.78	0.93	0.95	0.91	0.85	0.91	0.93
													*κ*	0.70	0.57	0.75	0.46	0.66	0.79	0.76
													SE	0.78	0.62	0.84	0.56	0.79	0.83	0.72
													SP	0.96	0.95	0.96	0.94	0.87	0.95	0.98
													PPV	0.78	0.62	0.72	0.46	0.76	0.87	0.90
													NPV	0.96	0.95	0.98	0.96	0.89	0.93	0.94
													F1	0.78	N/R	0.77	0.51	0.77	0.85	0.80
													MCC	0.70	0.57	0.75	0.46	0.66	0.79	0.77
da Silva Suoto et al. (2021)^54^	cEEGrid^85^	16	8 per ear	Wet	10 (M: 2, F: 8)	28.4 ± 4.3	Healthy	Home (devices fitted by a specialist)	1	EEG of Fpz, EOG_L and EOG_R scored by expert according to AASM	9341	Manual scoring	ACC	0.75	0.92	0.94	0.92	0.82	0.95	0.91
													*κ*	0.67	0.60	0.71	0.37	0.62	0.85	0.69
													SE	0.75	0.70	0.69	0.35	0.82	0.90	0.67
													SP	0.95	0.94	0.98	0.97	0.82	0.97	0.97
													PPV	0.75	0.67	0.80	0.50	0.74	0.87	0.83
													NPV	0.95	0.95	0.96	0.95	0.88	0.97	0.93
													F1	0.75	0.65	0.74	0.41	0.78	0.88	0.74
													MCC	0.67	0.61	0.71	0.38	0.62	0.85	0.70
Mikkelsen et al. (2019)^56^	cEEGrid^85^	16	8 per ear	Wet	15 (M: 6, F: 9)	35.3 ± 14.3	Healthy	Controlled	1	PSG scored by 2 experts according to AASM	18,920	Machine learning (Random Forest)	ACC	0.700^f^	N/R	N/R	N/R	N/R	N/R	N/R
													*κ*	0.600^f^	N/R	N/R	N/R	N/R	N/R	N/R
Sterr et al. (2018)^57^	cEEGrid^85^	16	8 per ear	Wet	15 (M: 6, F: 9)	35.3 ± 14.3	Healthy	Controlled	1	PSG scored by 2 experts according to AASM	18,920	Manual scoring	ACC	0.59^d^	N/R	N/R	N/R	N/R	N/R	N/R
													*κ*	0.42^d^	N/R	N/R	N/R	N/R	N/R	N/R

All evaluation metric values were calculated from confusion matrices provided in the original publications unless indicated otherwise.

*AASM* American Association of Sleep Medicine, *ACC* accuracy, *AD* Alzheimer’s disease, *ALS* amyotrophic lateral sclerosis, *BiLSTM* bidirectional long short-term memory, *CNN* convolutional neural network, *DSN* deep stacking networks, *EEG* electroencephalogram, *F* females, *F1* F1 score; *κ* Cohen’s kappa, *LDA* linear discriminant analysis; LightGBM: light gradient boosting machine; LSTM: long short-term memory, *M* males, *MCC* Matthews correlation coefficient, *NPV* negative predictive value, *OA* (*ma*) overall macro-averaged metrics, *OA* (*mi*) overall micro-averaged metrics; OSA: obstructive sleep apnea, *PD* Parkinson’s disease, *PPV* positive predictive value, *REM* rapid eye movement, *RNN* recurrent neural network, *RVM* relevance vector machine, *SE* sensitivity, *SP* specificity, *SVM* support vector machine, *WPSG-I* wearable polysomnogram, *N/R* not reported.

^a^The number of recording electrodes.

^b^For controlled environment studies, devices were fitted by researchers or technicians. For home studies, self-application vs. expert fitting is indicated in parentheses.

^c^In studies reporting manual scoring of wEEG data, scoring was conducted on raw signals from the device. As most wEEG devices lacked EOG and EMG channels, REM sleep was identified based on EEG characteristics alone, such as low-amplitude mixed-frequency activity.

^d^Reported in the original study.

^e^Calculated from reported metrics.

^f^Estimated from the graph.

**Table 2:** Overview of validation studies classifying 4 stages

Study	Device	# of el.^a^	El. position	El. Type	# of part.	Age	Part.	Env.^b^	Nights	Ref.	Epochs	Device scoring^c^		OA (mi)	OA (ma)	Wake	Light	Deep	REM
*Forehead*																			
*Headband*																			
González et al. (2024)^58^	Dreem^69^	5	F7, F8, O1, O2, Fpz	Dry	10 (M: 7, F: 3)	69.5 ± 7.6	PD	Controlled	1	PSG scored by an expert according to AASM	N/R	Dreem proprietary algorithm	ACC	N/R	N/R	0.78^d^	0.70^d^	0.95^d^	0.80^d^
													*κ*	N/R	N/R	0.75^d^	0.52^d^	0.93^d^	0.72^d^
													SE	N/R	N/R	0.54^d^	0.67^d^	0.87^d^	0.52^d^
													SP	N/R	N/R	0.92^d^	0.50^d^	0.95^d^	0.83^d^
Chen et al. (2023)^59^	Umind-Sleep^86^	1	N/R	Wet	197 (M: 148, F: 49)	37.05 ± 8.73	Mostly OSA (*n* = 171)	Controlled	1	PSG scored by experts according to AASM	195,349	Umindsleep proprietary algorithm	ACC	0.84	0.92	0.93	0.85	0.95	0.95
													*κ*	0.73	0.74	0.72	0.69	0.76	0.79
													SE	0.84	0.82	0.80	0.86	0.79	0.83
													SP	0.95	0.93	0.95	0.83	0.97	0.97
													PPV	0.84	0.80	0.73	0.88	0.79	0.82
													NPV	0.95	0.93	0.97	0.80	0.97	0.97
													F1	0.84	0.81	0.76	0.87	0.79	0.82
													MCC	0.73	0.74	0.72	0.69	0.76	0.79
Markwald et al. (2016)^60^	Zeo^71^	2	Fp1, Fp2 (Fpz as ref)	Dry	29 (M: 21, F: 8)	24.0 ± 5.3	Healthy	Controlled	1	PSG scored by an expert according to AASM	29,435	ZEO proprietary algorithm	ACC	0.67	0.87	0.91	0.73	0.86	0.86
													*κ*	0.50	0.41	0.49	0.46	0.53	0.58
													SE	0.67	NaN	0.40	0.71	0.67	0.75
													SP	0.92	0.90	0.98	0.76	0.90	0.89
													PPV	0.67	0.54	0.78	0.76	0.56	0.60
													NPV	0.92	0.90	0.92	0.71	0.94	0.94
													F1	0.67	NaN	0.53	0.73	0.61	0.67
													MCC	0.51	0.42	0.52	0.46	0.53	0.58
Cellini et al. (2015)^61^	Zeo^71^	2	Fp1, Fp2 (Fpz as ref)	Dry	30 (M: 13, F: 17)	20.3 ± 2.76	Healthy	Controlled	1 nap	PSG scored by an expert according to AASM	4938	ZEO proprietary algorithm	ACC	N/R	0.83^d^	0.87^d^	0.75^d^	0.91^d^	0.81^d^
													*κ*	N/R	0.53^d^	0.62^d^	0.49^d^	0.72^d^	0.28^d^
													SE	N/R	0.73^d^	0.59^d^	0.67^d^	0.84^d^	0.82^d^
													PPV	N/R	0.64^d^	0.87^d^	0.76^d^	0.72^d^	0.22^d^
													F1	N/R	0.63^e^	0.70^e^	0.71^e^	0.77^e^	0.35^e^
*Sleepmask*																			
Rostaminia et al. (2022)^62^	PhyMask prototype developed in the study^62^	2	Near Fp1, Fp2 (Fpz as ref and ground)	Wet	1 (M: 1, F: 0)	N/R	Healthy	Home (devices fitted by a specialist)	5	PSG scored by an expert according to AASM	4235	Manual scoring	ACC	0.82	0.91	0.96	0.84	0.96	0.89
													*κ*	0.73	0.70	0.54	0.68	0.89	0.69
													SE	0.82	0.81	0.63	0.77	0.87	0.95
													SP	0.94	0.94	0.97	0.91	0.99	0.88
													PPV	0.82	0.75	0.50	0.90	0.97	0.63
													NPV	0.94	0.93	0.98	0.79	0.95	0.99
													F1	0.82	0.77	0.56	0.83	0.92	0.76
													MCC	0.74	0.71	0.54	0.69	0.89	0.71
Hsieh et al. (2021)^63^	Prototype developed in the study^63^	2	Fp1, EOG R	Dry	25 (M: 12, F:13)	23.2 ± 1.8	Healthy	Controlled	1	PSG scored by an expert according to AASM	7482	Deep learning (MobileNetV2 based on CNN)	ACC	0.87	0.93	0.97	0.87	0.95	0.95
													*κ*	0.79	0.81	0.83	0.74	0.82	0.83
													SE	0.87	0.86	0.85	0.87	0.83	0.89
													SP	0.96	0.95	0.98	0.87	0.97	0.96
													PPV	0.87	0.86	0.85	0.88	0.88	0.84
													NPV	0.96	0.95	0.98	0.86	0.96	0.97
													F1	0.87	0.86	0.85	0.87	0.85	0.87
													MCC	0.79	0.81	0.83	0.74	0.82	0.83
*Neck*																			
Kaplan et al. (2015)^64^	Zmachine Insight+^87^	2	A1, A2, reference located on back of the neck	Wet	99 (M:47, F: 52)	18–60 (median 32.7)	Healthy, Insomnia, Apnea, PLM/RLS, SSRI/SNRI	Controlled	1	PSG scored by experts according to Rechtschaffen and Kales^88^	85,206	Z-PLUS Proprietary algorithm	ACC	0.82	0.91	0.95	0.83	0.96	0.90
													*κ*	0.72	0.73	0.84	0.66	0.74	0.67
													SE	0.82	0.80	0.91	0.84	0.74	0.72
													SP	0.94	0.93	0.96	0.82	0.98	0.94
													PPV	0.82	0.80	0.84	0.85	0.78	0.73
													NPV	0.94	0.93	0.98	0.81	0.98	0.94
													F1	0.82	0.80	0.87	0.84	0.76	0.73
													MCC	0.72	0.73	0.84	0.66	0.74	0.67

All evaluation metric values were calculated from confusion matrices provided in the original publications unless indicated otherwise.

*AASM* American Association of Sleep Medicine, *ACC* accuracy, *CNN* convolutional neural network, *F* females, *F1* F1 score, *κ* Cohen’s kappa, *M* males, *MCC* Matthews correlation coefficient, *NPV* negative predictive value, *OA (ma)* overall macro-averaged metrics, *OA (mi)* overall micro-averaged metrics, *OSA* obstructive sleep apnea, *PD* Parkinson’s disease, *PLM/RLS* periodic limb movements/restless legs syndrome, *PPV* positive predictive value, *PSG* polysomnograph, *REM* rapid eye movement, *SE* sensitivity, *SNRI* serotonin-norepinephrine reuptake inhibitor, *SP* specificity, *SSRI* selective serotonin reuptake inhibitor, *N/R* not reported.

^a^The number of recording electrodes.

^b^For controlled environment studies, devices were fitted by researchers or technicians. For home studies, self-application vs. expert fitting is indicated in parentheses.

^c^In studies reporting manual scoring of wEEG data, scoring was conducted on raw signals from the device. As most wEEG devices lacked EOG and EMG channels, REM sleep was identified based on EEG characteristics alone, such as low-amplitude mixed-frequency activity.

^d^Reported in original study.

^e^Calculated from reported metrics.

**Table 3:** Overview of validation studies using other than five- or four-stage systems

Study	Device	# of el.^a^	El. Position	El. Type	# of part.	Age [years]	Part.	Env.	Nights	Ref.	Epochs	Device scoring^b^		ACC	*κ*	SE	SP	PPV	NPV	F1	MCC
*Forehead*																					
*Headband*																					
Zhang et al. (2024)^65^	LANMAO sleep recorder (prototype)^65^	2	Fp1, Fp2	Wet	34 (M: 16, F: 18)	4.28 ± 1.61 (1-7) days	Healthy	Controlled	1	PSG scored by 2 experts according to AASM	N/R	LANMAO proprietary algorithm	OA (mi.)	0.80^c^	0.65^c^	N/R	N/R	N/R	N/R	0.77^c^	N/R
													Wake	0.87^c^	0.64^c^	N/R	N/R	N/R	N/R	0.74^c^	N/R
													NREM	0.86^c^	0.74^c^	N/R	N/R	N/R	N/R	0.80^c^	N/R
													REM	0.81^c^	0.63^c^	N/R	N/R	N/R	N/R	0.74^c^	N/R
Lucey et al. (2016)^67^	X4 Sleep Profiler^70^	2	AF7, AF8 (Fpz as ref)	Dry	29 (M: 17, F: 12)	54 ± 15.7 (25–80)	Sleep disorders	Controlled	1	PSG scored by 2 experts according to AASM	19326	Manual scoring	OA (mi.)	N/R	0.67^c^	N/R	N/R	N/R	N/R	N/R	N/R
													OA (ma.)	0.90^d^	N/R	0.68^d^	0.93^d^	0.66^d^	0.92^d^	0.67^d^	N/R
													Wake	0.89^c^	N/R	0.21^c^	0.96^c^	0.29^c^	0.93^c^	0.72^d^	N/R
													N1	0.90^c^	N/R	0.21^c^	0.96^c^	0.29^c^	0.93^c^	0.24^d^	N/R
													N2&N3	0.88^c^	N/R	0.87^c^	0.89^c^	0.91^c^	0.84^c^	0.89^d^	N/R
													REM	0.94^c^	N/R	0.86^c^	0.95^c^	0.76^c^	0.98^c^	0.81^d^	N/R
*Ear*																					
*In-ear*																					
Nakamura et al. (2017)^68^	Prototype developed in Goverdovsky et al. (2016)^74^, (2017)^75^	4	2 per ear	Wet	4 (M:4, F:0)	25–36	Healthy	Controlled	1 nap	Scalp-EEG scored by an expert according to AASM	293	Machine learning (SVM)	OA (mi.)	0.77	0.65	0.77	0.92	0.77	0.92	0.77	0.65
													OA (ma.)	0.88	0.63	0.70	0.91	0.74	0.92	0.72	0.63
													Wake	0.90	0.72	0.79	0.93	0.78	0.94	0.79	0.72
													N1	0.88	0.50	0.50	0.95	0.66	0.91	0.57	0.51
													N2	0.84	0.67	0.89	0.78	0.79	0.89	0.84	0.68
													N3	0.92	0.62	0.60	0.97	0.75	0.94	0.67	0.63
Palo et al. (2024)^66^	IDUN Guardian Development Kit^89^	2	1 per ear	Dry	10	18–60	Healthy	Controlled	1	PSG scored by 3 experts according to AASM	4568	Manual scoring	Wake	N/R	N/R	0.80^c^	N/R	0.84^c^	N/R	0.79^c^	N/R
													NREM	N/R	N/R	0.95^c^	N/R	0.88^c^	N/R	0.91^c^	N/R
													REM	N/R	N/R	0.47^c^	N/R	0.65^c^	N/R	0.53^c^	N/R

All evaluation metric values were calculated from confusion matrices provided in the original publications unless indicated otherwise.

*ACC* accuracy, *AASM* American Academy of Sleep Medicine, *EEG* electroencephalogram, *El.* electrode, *Env.* environment, *F* females, *F1* F1 score, *M* males, *MCC* Matthews correlation coefficient, *NPV* negative predictive value, *NREM* non-rapid eye movement, *OA (mi.)* overall micro-averaged metrics, *OA (ma.)* overall macro-averaged metrics, *Part.* participants, *PPV* positive predictive value, *PSG* polysomnography, *REM* rapid eye movement, *SE* sensitivity, *SP* specificity, *SVM* support vector machine, *κ* Cohen’s kappa, *N*/*R* not reported.

^a^The number of recording electrodes.

^b^In studies reporting manual scoring of wEEG data, scoring was conducted on raw signals from the device. As most wEEG devices lacked EOG and EMG channels, REM sleep was identified based on EEG characteristics alone, such as low-amplitude mixed-frequency activity.

^c^Reported in original study.

^d^Calculated from reported metrics.

### Overview of study characteristics

**Fig. 1:**
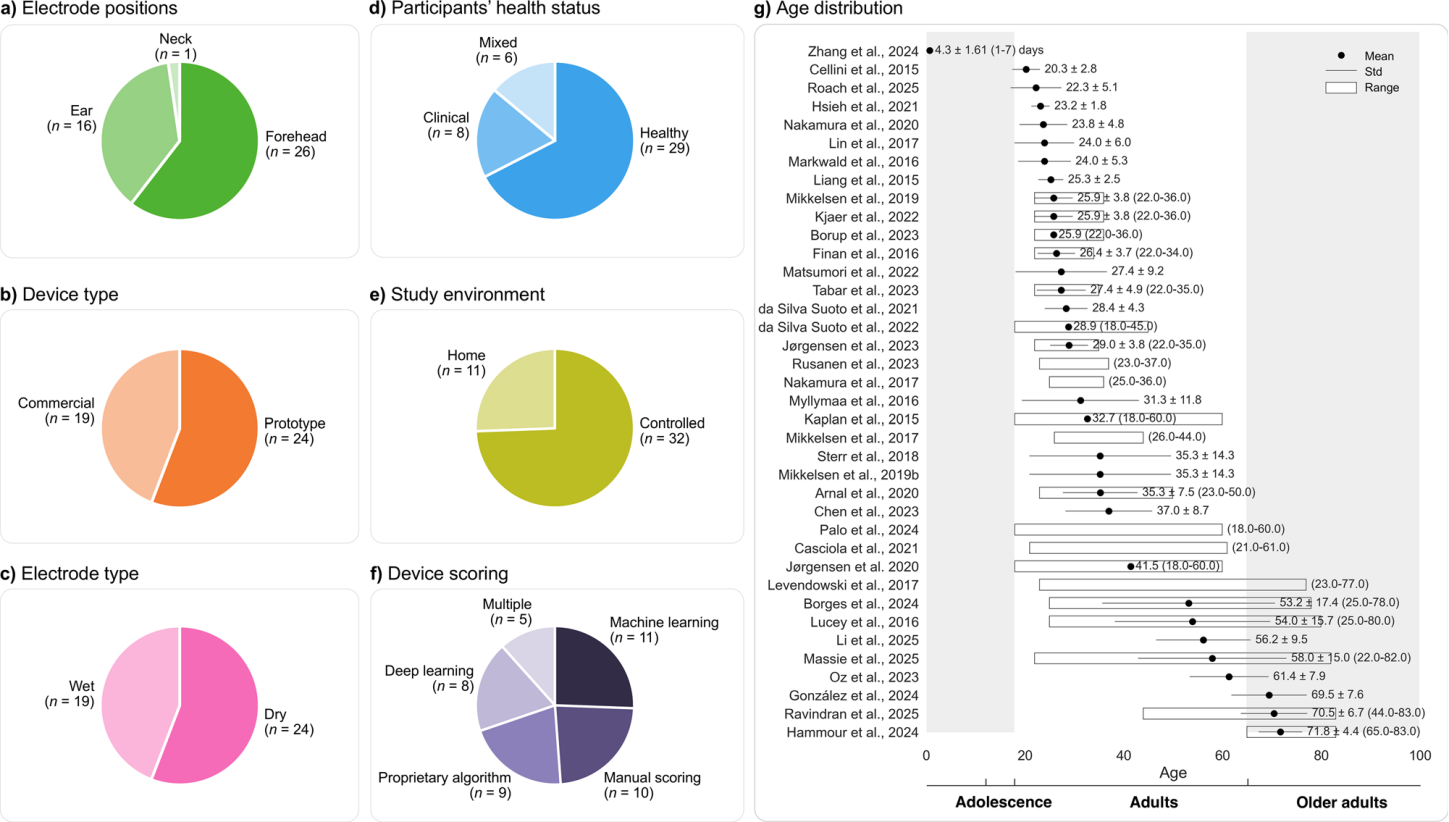
Characteristics of the 43 included validation studies. **a** *Electrode positions*: Most studies used forehead EEG (*n* = 26), followed by ear-based devices (*n* = 16); one study used a neck-worn sensor. **b** *Device type***:** Prototype devices (*n* = 24) were more common than commercially available systems (*n* = 19). **c** *Electrode type*: Dry electrodes were used in 24 studies, while 19 employed wet electrodes. **d** *Participants’ health status*: Most studies involved healthy participants (*n* = 29), with fewer including clinical populations (*n* = 8) or mixed cohorts (*n* = 6). **e** *Study environment*: The majority of studies were conducted in controlled environments (*n* = 32), while 11 were home-based. **f** *Device scoring method*: Machine learning (*n* = 11), manual scoring (*n* = 10), and proprietary algorithms (*n* = 9) were the most common, with some studies using deep learning (*n* = 8) or multiple methods (*n* = 5). (**g**) *Age distribution*: Most studies involved adults in their mid-20s to mid-30s. Mean age, standard deviation (black lines), and range (white bars) are shown. Older adults and adolescents were underrepresented.

**Fig. 2:**
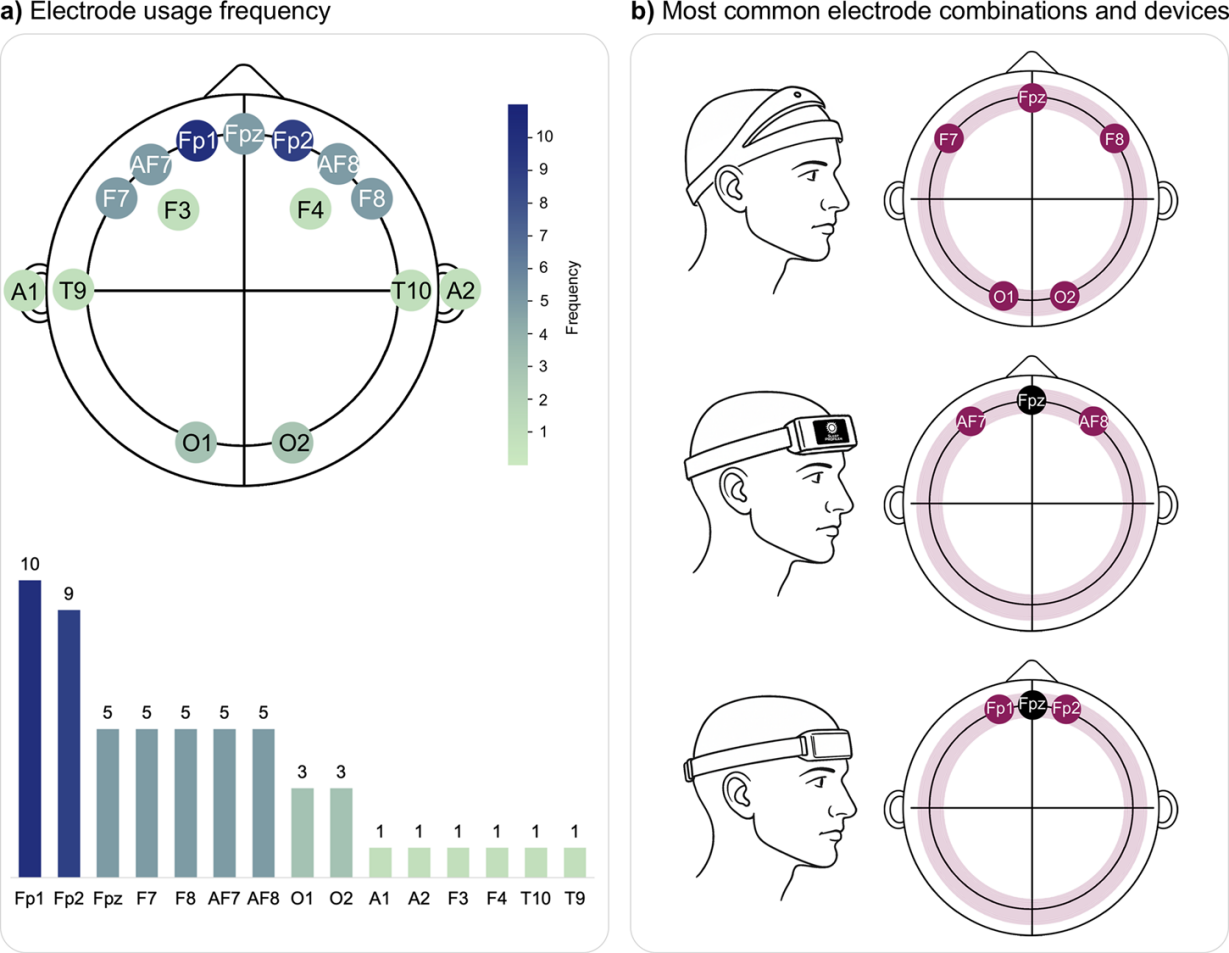
Overview of electrode use across forehead-EEG studies. **a** *Electrode usage frequency*: Scalp heatmap and bar chart showing how often each electrode was used in included studies. Scalp heatmap and bar chart showing the frequency of electrode positions used in studies with forehead-placed EEG, based on the international 10–20 system. The most frequently used electrodes were Fp1 and Fp2, followed by Fpz, F7, F8, AF7, and AF8. **b** *Most common electrode combinations and corresponding devices*: The three most common electrode combinations and their associated device types. Top: Dreem headband (Fpz, F7, F8, O1, O2); Middle: Sleep Profiler (Fpz, AF7, AF8); Bottom: Zeo headband (Fp1, Fpz, Fp2). While Dreem and Sleep Profiler were each used in three studies, the Fp1–Fp2–Fpz combination appeared in five studies, including two using the Zeo device.

**Fig. 3:**
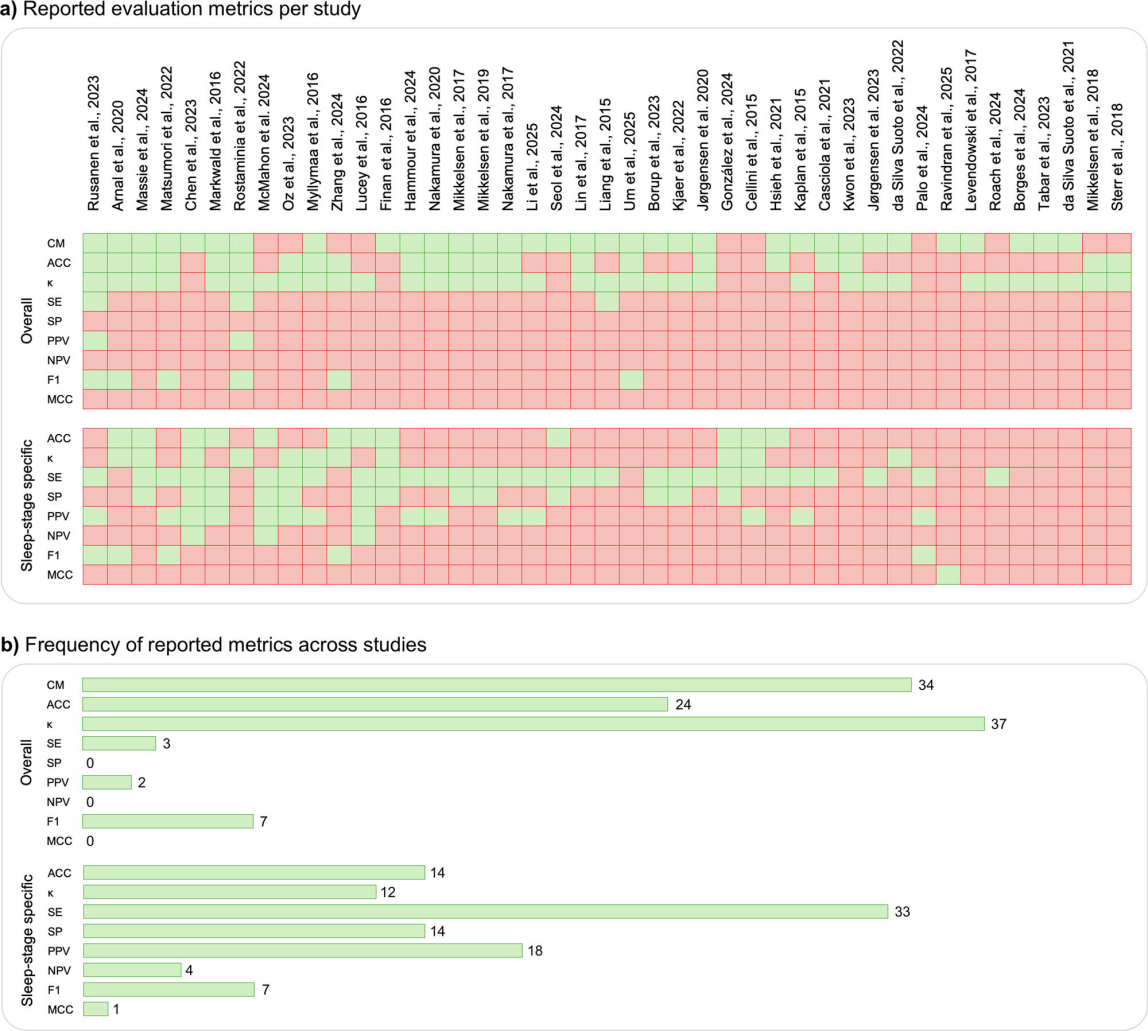
Evaluation metrics reported across included studies. **a** *Reported evaluation metrics per study*: Presence (green) or absence (red) of overall and sleep-stage-specific evaluation metrics reported in each study. **b** *Frequency of reported metrics across studies*. Total number of studies reporting each evaluation metric, grouped by overall vs. stage-specific metrics. For devices’ overall performance, *κ* (*n* = 37) and ACC (*n* = 24) were the most frequently reported metrics. For sleep-stage specific performance, SE (*n* = 33) and PPV (*n* = 18) were the most frequently reported metrics. Confusion matrices were provided in 34 studies. ACC: accuracy, CM: confusion matrix, *κ*: Cohen’s Kappa, MCC: Matthews correlation coefficient, NPV: negative predictive value, PPV: positive predictive value, SE: sensitivity, SP: specificity.

Figure 1 summarizes key characteristics of the 43 validation studies included in this review. Most validation studies involved single-night sleep recordings (*n* = 34), were conducted in controlled environments such as sleep laboratories (*n* = 32), and primarily included healthy participants (*n* = 29). Home-based studies (*n* = 11) were generally set up by professionals, with the exception of one study^47^, where participants fitted the device themselves. Only a few studies included clinical populations (*n* = 8; e.g., obstructive sleep apnea^29,36,39,44,59^, epilepsy^50^, Parkinson’s disease^58^, sleep disorders^67^ and six studies involved mixed cohorts, including both healthy and clinical participants—for example, one study^58^ primarily enrolled healthy individuals (*n* = 50) but also included a subgroup of patients with Alzheimer’s disease (*n* = 12). The number of participants per study ranged from 1 to 197 (mean = 30 ± 37). The volume of data analyzed ranged widely, from 293 to 195,349 epochs (mean = 29,969 ± 37,072), although epoch counts were not available for four studies. Gender representation was approximately balanced among those reporting it (57% male, 43% female), though five studies did not provide gender breakdowns. Participant age was skewed toward young to middle-aged adults, with limited inclusion of adolescents and older adults. wEEGs used across studies differed in electrode placement, form factor, and electrode type. Of the 43 included studies, 19 evaluated commercially available wEEG devices—most frequently Dreem^28,32,58^ and Sleep Profiler^22,34,67^, and Somfit^37,38^ and Zeo^60,61^, while 24 assessed prototypes. Electrode placement was most commonly on the forehead (*n* = 26), followed by the ear (*n* = 16), with one study^64^ using a neck-worn device. Most devices with forehead electrodes were headbands (*n* = 15), but other designs included sheet-like or patch-based systems (*n* = 8) and sleep masks (*n* = 3). Electrode usage patterns in forehead devices also revealed strong clustering around a few standard positions. As shown in Fig. 2, Fp1 and Fp2 were the most commonly used electrodes, followed by Fpz, F7, F8, AF7, and AF8. The three most frequent electrode combinations corresponded to well-known devices: the Dreem headband^69^ (Fpz, F7, F8, O1, O2), the Sleep Profiler^70^ (Fpz, AF7, AF8), and the Zeo headband^71^ (Fp1, Fpz, Fp2). Notably, the Fp1–Fp2–Fpz configuration appeared in five studies, including two that used the Zeo device and three others^30,62,65^. Among ear-based devices, the majority were in-ear designs (*n* = 12), with a smaller number of around-the-ear configurations (*n* = 4). The number of electrodes ranged from 1 to 16 (mean = 6 ± 4). Dry electrodes were used in 24 studies, while 19 employed wet electrodes. Sleep staging methods applied to the wEEG data varied between machine learning (*n* = 11), manual scoring (*n* = 10), proprietary algorithms (*n* = 9), and deep learning (*n* = 8). Five studies^22,27,31,34,41^ used a combination of methods, meaning they reported results from more than one approach (e.g., deep learning alongside manual scoring by an expert). To evaluate sleep staging accuracy, most studies used expert-scored PSG as the reference standard, adhering to AASM guidelines (*n* = 40). Ground truth data were typically scored by a single rater (*n* = 25). Reported evaluation metrics varied across studies: overall device performance was most commonly assessed using Cohen’s Kappa (*n* = 37) and accuracy (*n* = 24), while stage-specific metrics such as sensitivity (*n* = 33) and positive predictive value (*n* = 18) were frequently used. Confusion matrices were provided in 34 studies (Fig. 3).

### Analysis of validation studies

**Fig. 4:**
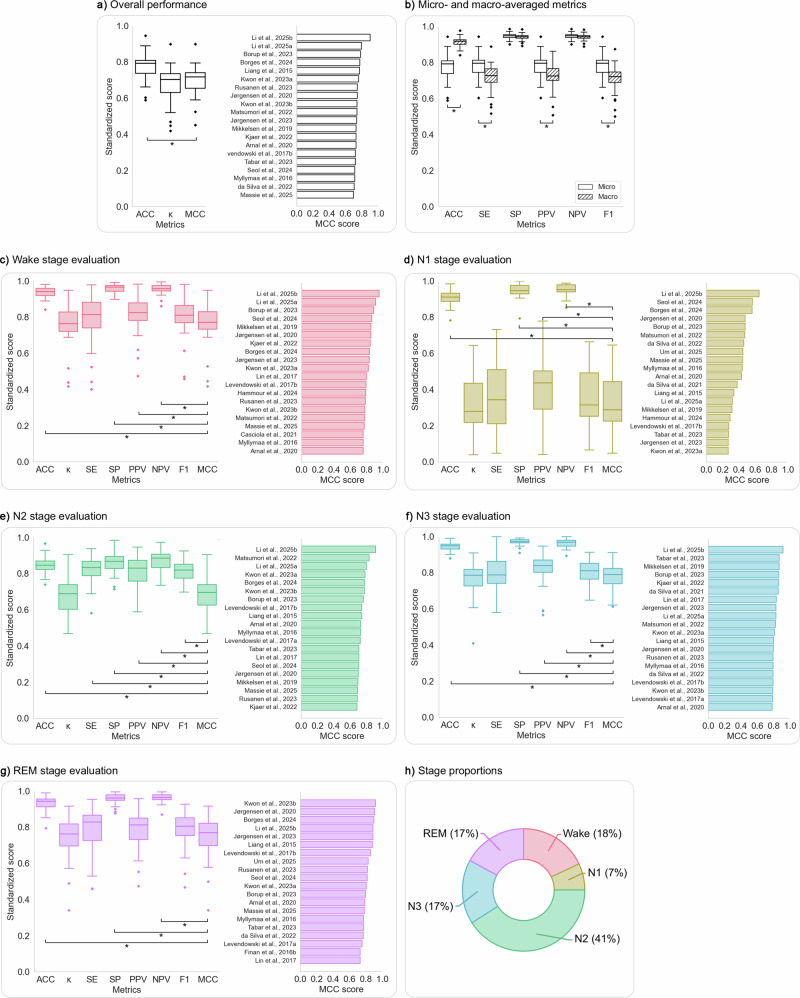
Evaluation metrics for studies classifying five sleep stages using wEEGs. Each panel (**a**, **c**–**g**) includes a boxplot of metric distributions and a bar chart showing the top 20 studies by MCC score for that specific stage. Asterisks (*) indicate statistically significant differences (*p* < 0.05) from MCC (**a**, **c**–**g**) or between micro- and macro-averaged versions of the same metric in (**b**). **a** *Overall metrics***:** Results demonstrated that overall accuracy (ACC) was significantly higher than Cohen’s kappa (*κ*) and MCC. **b** *Comparison of micro- and macro-averaged metrics***:** Macro-averaged ACC was significantly higher than micro-averaged ACC, while micro-averaged SE, PPV, and F1 were significantly higher than their macro-averaged counterparts, highlighting class imbalance effects. **c** *Wake stage*: Wake showed the highest SP and NPV but the lowest SE among stages, indicating reliable exclusion of wake but variable detection. **d** *N1 stage:* N1 was the most challenging stage, with the lowest scores across *κ*, SE, PPV, F1, and MCC. **e** *N2 stage*: Showed relatively high metrics overall. Being the most prevalent class, the differences between metrics emphasizing TPs (e.g., SE) and TNs (e.g., SP) were smaller compared to other stages. Nevertheless, the MCC and *κ* scores were noticeably lower, suggesting potential misclassifications. **f** *N3 stage*: N3 showed the highest *κ* and MCC, with strong SE and excellent agreement across studies. **g** *REM stage***:** REM had the second-highest SE and MCC but the lowest PPV, indicating a tendency for false positives. **h** *Mean proportional distribution of sleep stages across studies*: Wake (18%), N1 (7%), N2 (41%), N3 (17%), REM (17%). ACC: accuracy; *κ*: Cohen’s kappa; MCC: Matthews correlation coefficient; SE: sensitivity; SP: specificity; PPV: positive predictive value; NPV: negative predictive value.

**Fig. 5:**
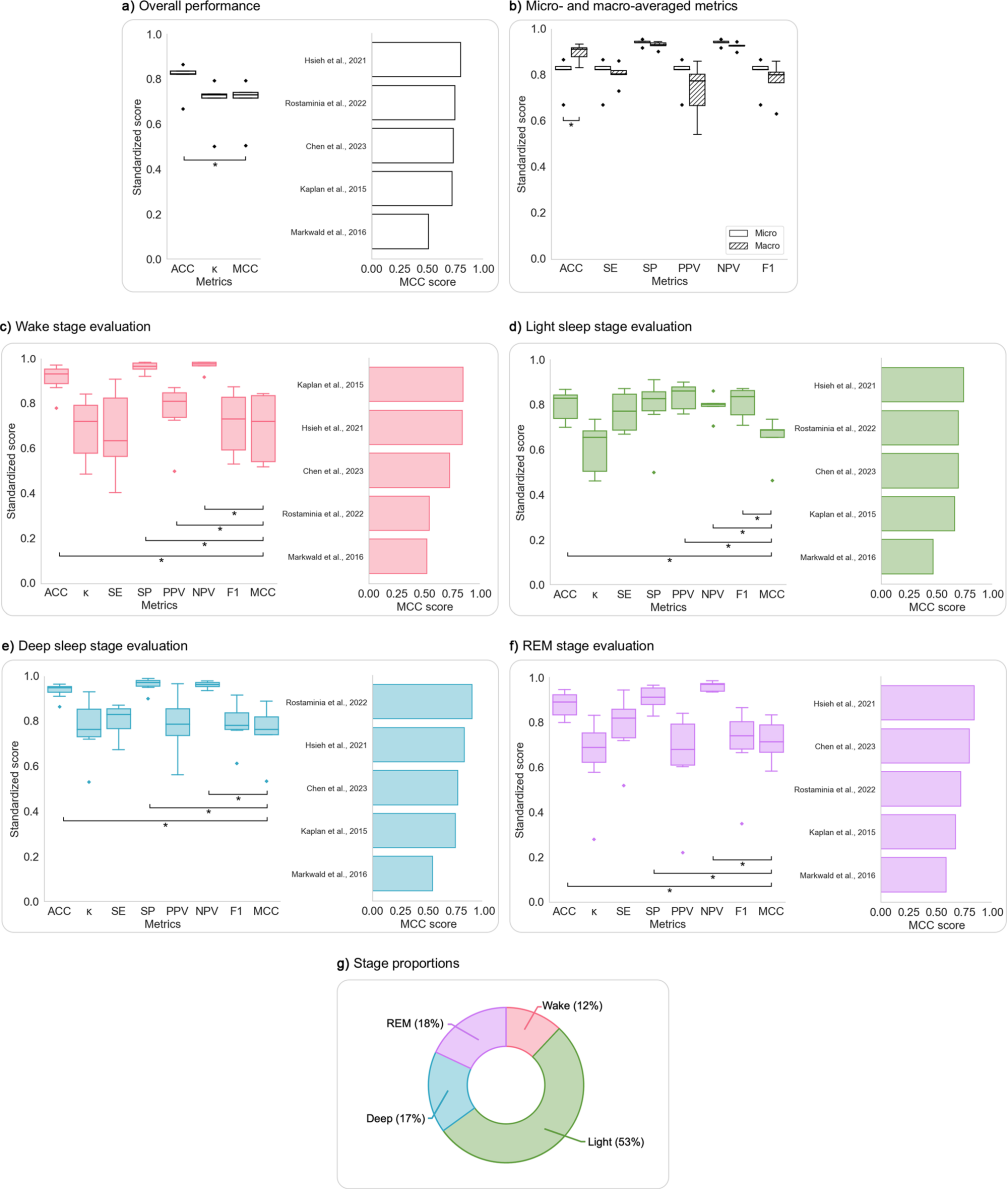
Evaluation metrics for studies classifying four sleep stages using wEEGs. Each panel (**a**, **c**–**g**) includes a boxplot of metric distributions and a bar chart showing the top studies by MCC score for that specific stage. Asterisks (*) indicate statistically significant differences (*p* < 0.05) from MCC (**a**, **c**–**g**) or between micro- and macro-averaged versions of the same metric in (**b**). **a** *Overall metrics*: Studies showed an average ACC of 0.80 ± 0.08, significantly higher than *κ* (0.70 ± 0.11) and MCC (0.70 ± 0.11). **b** *Comparison of micro- and macro-averaged metrics*: Macro-averaged ACC was significantly higher than micro-averaged ACC. **c** *Wake*: Demonstrated *κ* = 0.68 ± 0.14, MCC = 0.69 ± 0.16, highest NPV and lowest SE (0.68 ± 0.18), suggesting strong ability to exclude Wake epochs but weaker sensitivity. **d** *Light sleep***:** The most prevalent stage (53% of epochs) had the lowest *κ* (0.61 ± 0.11) and MCC (0.65 ± 0.11), along with the lowest ACC, SP, and NPV, but highest PPV*.* **e** *Deep sleep*: Showed the strongest performance overall, with the highest *κ* (0.77 ± 0.13), MCC (0.75 ± 0.13), ACC, and SE (0.80 ± 0.07), indicating robust detection. **f** REM: Achieved high SE (0.78 ± 0.14), *κ* (0.65 ± 0.18), and MCC (0.72 ± 0.10), but had the lowest PPV, suggesting higher false positives in REM classification. **g** *Mean stage distribution across studies*: Light sleep (53%), Deep sleep (17%), REM (18%), and Wake (12%). ACC: accuracy; *κ*: Cohen’s kappa; MCC: Matthews correlation coefficient; SE: sensitivity; SP: specificity; PPV: positive predictive value; NPV: negative predictive value.

The overview of overall and sleep-stage-specific evaluation metrics, along with the proportions of each sleep stage for both five- and four-stage studies, are presented in Figs. 4 and 5, respectively. The data for these figures can be found in Supplementary Tables S1–S4. Additionally, the Bland–Altman plots, which offer insights into variations in sleep stage duration and proportion estimations by wEEGs compared to PSG, are detailed in Supplementary Figs. S1 and S2.

### Studies classifying Wake, N1, N2, N3, REM

On average, the studies showed an overall ACC of 0.78 ± 0.07 and an overall *κ* of 0.68 ± 0.10, suggesting substantial agreement between wEEGs and PSG. MCC (0.70 ± 0.08) and *κ* were significantly lower compared to ACC. Significant differences were also observed between micro- and macro-averaged ACC, SE, PPV and F1, highlighting the effect of class imbalance on evaluation outcomes. The top three overall MCC results were reported by Li et al.^27^ (WPSG-I headband; MCC = 0.899 for manual scoring and 0.795 for automatic scoring) and Borup et al.^46^ (in-ear prototype^72^; MCC = 0.784) (Fig. 4a, b).

The Wake stage, representing 18% of epochs, showed strong agreement between wEEGs and PSG with a mean *κ* of 0.75 ± 0.12, MCC of 0.76 ± 0.12, and SE of 0.79 ± 0.13. It had the highest accuracy among all stages. Bland–Altman analysis revealed a slight underestimation by wearable devices in duration (–3.20 min; LoA: –21.13 to +14.73 min) and in proportion (–0.69%; LoA: –4.67 to +3.30%). High ICCs (1.00 for duration, 0.99 for proportion) further supported the robustness of these measurements. The best Wake-stage MCCs were reported by Li et al.^27^ (WPSG-I headband; MCC = 0.950 and 0.906) and Borup et al.^46^ (in-ear prototype^72^; MCC = 0.883) (Fig. 4c).

The N1 stage, comprising just 7% of all epochs, showed the weakest agreement between wEEGs and PSG. It had the lowest mean *κ* (0.31 ± 0.15), MCC (0.33 ± 0.15), and SE (0.36 ± 0.18) of all stages, and exhibited high variability across studies, reflecting inconsistent detection. It also had the lowest average PPV. Duration was marginally overestimated (+0.64 min; LoA: –33.83 to +35.12 min), and proportion slightly overestimated (+0.32%; LoA: –7.83% to +8.47%). ICCs were 0.75 and 0.72 for duration and proportion, respectively, indicating moderate-to-good agreement. Top-performing results for N1-stage MCC were reported by Li et al.^27^ (WPSG-I headband; MCC = 0.646), Seol et al.^29^ (Insomnograf K2 headband^73^; MCC = 0.562), and Borges et al.^44^ (in-ear prototype^74,75^; MCC = 0.558) (Fig. 4d).

The N2 stage, the most prevalent (41% of epochs), demonstrated strong classification performance with a mean SE of 0.82 ± 0.08, while *κ* and MCC were slightly lower (both 0.69 ± 0.09). It showed the highest SE and F1 across stages, but the lowest ACC, SP, and NPV. wEEGs slightly overestimated N2 duration (+1.51 min; LoA: –46.75 to +49.78 min) and proportion (+0.23%; LoA: –11.09% to +11.55%). ICCs were 0.79 and 0.73 for duration and proportion, respectively, indicating good agreement. Top MCC results were reported by Li et al.^27^ (WPSG-I headband; MCC = 0.906 and 0.794 for manual and automatic scoring, respectively) and Matsumori et al.^42^ (sheet-like prototype device; MCC = 0.829) (Fig. 4e).

The N3 stage, also known as Deep Sleep, representing 17% of epochs, had the highest *κ* (0.76 ± 0.10), MCC (0.78 ± 0.07), and SP, PPV, and NPV among all stages, with a high SE of 0.79 ± 0.10. wEEGs slightly overestimated N3 duration (+1.93 min; LoA: –21.37 to +25.70 min) and proportion (+0.38%; LoA: –4.95 to +4.20%). ICCs of 0.93 for both duration and proportion reflect excellent agreement. Leading results came from Li et al.^27^ (WPSG-I headband; MCC = 0.912), Tabar et al.^47^ (in-ear prototype; MCC = 0.864), and Mikkelsen et al.^52^ (in-ear prototype^72^; MCC = 0.863) (Fig. 4f).

The REM stage, also accounting for 17% of epochs, showed strong performance with SE = 0.79 ± 0.13, *κ* = 0.74 ± 0.13, and MCC = 0.75 ± 0.13. Duration and proportion were slightly overestimated (+1.90 min; LoA: –15.56 to +19.36 min; +0.36%; LoA: –4.95% to +4.20%). ICCs were 0.95 and 0.93, indicating excellent reliability. The highest REM MCCs were observed in Kwon et al.^41^ (sheet-like patch prototype; MCC = 0.917), Jørgensen et al.^50^ (in-ear prototype^72^; MCC = 0.903), and Borges et al.^44^ (in-ear prototype^74,75^; MCC = 0.894) (Fig. 4g).

In the Wake, N1, N3, and REM stages, significant differences were observed between ACC, SP, PPV, NPV and the balanced metric MCC, indicating that high values of these metrics may mask poor classification performance in minority classes.

### Studies classifying Wake, Light Sleep, Deep Sleep and REM

Studies classifying four stages showed an average overall ACC of 0.80 ± 0.08, and *κ* and MCC of 0.70 ± 0.11, indicating substantial agreement between wEEGs and PSG. Significant difference was observed between micro- and macro-averaged ACC (Fig. 5a, b).

The Wake stage, comprising 12% of all epochs, showed notable variability across studies. It had a mean *κ* of 0.68 ± 0.14, MCC of 0.69 ± 0.16, and the lowest SE among all stages (0.68 ± 0.18), but achieved the highest NPV. According to Bland–Altman analysis, Wake duration was slightly overestimated (+2.75 min; LoA: –31.56 to +26.07 min), with near-zero bias in proportion (+0.01%; LoA: –0.07% to +0.07%). ICCs were 0.90 for duration and 0.86 for proportion, indicating strong agreement (Fig. 5c).

The Light Sleep stage, constituting 53% of all epochs, but showed the lowest mean *κ* (0.61 ± 0.11), MCC (0.65 ± 0.11), ACC, SP, and NPV. In contrast, it had the highest PPV and a solid SE (0.77 ± 0.09). Bland-Altman analysis showed a small underestimation in duration (–1.22 min; LoA: –33.39 to +30.95 min) and proportion (–0.03%; LoA: –0.14 to +0.09%). Duration agreement was excellent (ICC = 0.98), but proportion agreement was poor (ICC = –0.07), suggesting inconsistency in proportional estimates (Fig. 5 d).

The Deep Sleep stage (N3), making up 17% of epochs, showed the highest *κ* (0.77 ± 0.13), MCC (0.75 ± 0.13), ACC and SE (0.80 ± 0.07). Duration was slightly overestimated (+0.28 min; LoA: –17.03 to +17.56 min), with minimal bias in proportion (–0.01%; LoA: –0.07% to +0.07%). ICCs were strong for both for duration (ICC = 0.97) and proportion (ICC = 0.86) (Fig. 5e).

The REM stage, accounting for 18% of epochs, had high SE (0.78 ± 0.14), MCC (0.72 ± 0.10), and *κ* (0.65 ± 0.18), but the lowest PPV, suggesting more frequent false positives. Duration was slightly overestimated (+1.28 min; LoA: –17.45 to +20.02 min), with minimal bias in proportion (+0.03%; LoA: –0.04% to +0.13%). Agreement on duration was good (ICC = 0.80), but poor for proportion (ICC = –0.31) (Fig. 5f).

In the Wake, Deep Sleep and REM stages, significant differences between ACC, SP, NPV and MCC were observed.

### Influence of methodological variabilities on wEEG performance

The impact of methodological variabilities on the wEEG’s performance in detecting sleep stages were analyzed, focusing on five-stage classification studies due to the limited sample size of four-stage studies. Several significant associations between study design features and evaluation metrics suggest that methodological choices can affect reported performance.

**Fig. 6:**
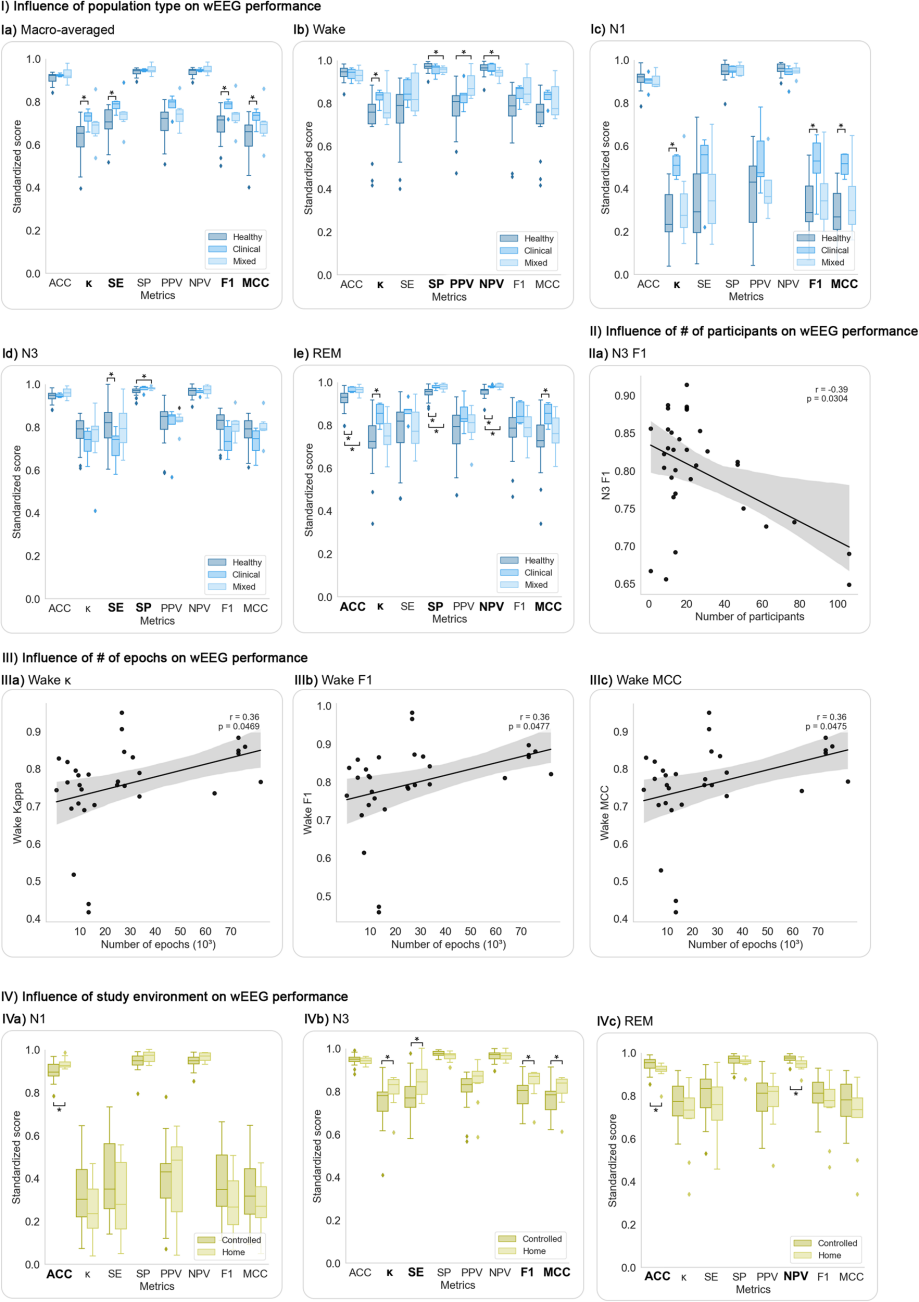
Study-level factors significantly associated with variations in wEEG performance. This figure presents only those comparisons where significant differences (*p* < 0.05) in evaluation metrics were observed. Full analyses across all stages and variables—including non-significant results—are available in the Supplementary figures. Asterisks (*) indicate statistically significant group differences based on Mann–Whitney *U* tests. **I** *Influence of participants’ health status on wEEG performance*: Boxplots show statistically significant differences between healthy, clinical, and mixed populations in macro-averaged metrics (**Ia**), Wake (**Ib**), N1 (**Ic**), N3 (**Id**), and REM (**Ie**) stages. Clinical and mixed-population studies generally reported higher performance, especially for *κ*, SE, F1, and MCC. **II** *Influence of number of participants*: Pearson correlation analysis revealed a significant negative correlation between the number of participants and N3 F1 score, suggesting reduced consistency in larger, more heterogeneous cohorts. **III** *Influence of number of epochs*: Significant positive correlations were found between the number of epochs and Wake-stage *κ*, F1 and MCC, indicating improved Wake classification performance with greater data volume. **IV** *Influence of study environment*: Boxplots comparing controlled (e.g., sleep lab or hospital) and home-based settings show significantly better performance in home studies for N1 ACC (**IVa**) and N3-stage *κ*, SE, F1, and MCC (**IVb**). Controlled settings yielded higher REM-stage ACC and NPV (**IVc**). ACC: accuracy; *κ*: Cohen’s kappa; MCC: Matthews correlation coefficient; SE: sensitivity; SP: specificity; PPV: positive predictive value; NPV: negative predictive value; F1: F1 score.

wEEG performance varied by participant health status: studies involving clinical or mixed populations showed significantly higher performance across macro-averaged and Wake, N1, N3, and REM metrics compared to studies only including healthy participants (Fig. 6I). N3 F1 performance was negatively associated with the number of participants, possibly reflecting greater heterogeneity in larger samples (Fig. 6II). In contrast, Wake-stage *κ*, F1, and MCC were positively associated with the number of epochs, indicating more robust classification with larger datasets (Fig. 6III). Study environment also influenced performance: home-based studies showed significantly higher N1 ACC and N3 *κ*, SE, F1, and MCC, while controlled environments yielded higher REM-stage ACC and NPV (Fig. 6IV). However, limited results for clinical, mixed, and home-based subgroups may restrict the generalizability of these findings.

**Fig. 7:**
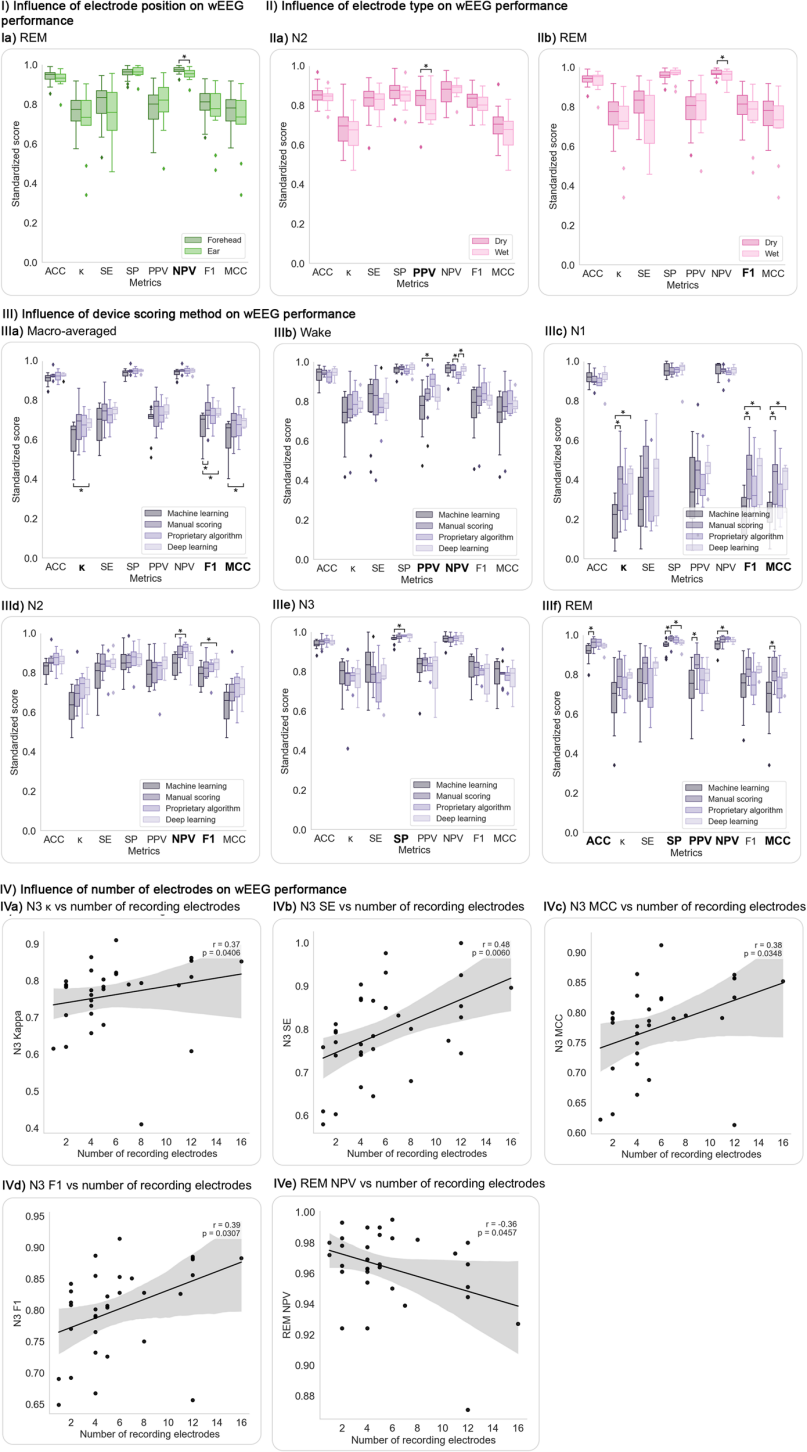
Device- and electrode-related factors associated with significant differences in wEEG performance. This figure presents only those comparisons where significant differences (*p* < 0.05) in evaluation metrics were observed. Full analyses across all stages and variables—including non-significant results—are available in the Supplementary figures. Asterisks (*) indicate statistically significant group differences based on Mann–Whitney *U* tests. **I** *Electrode position*: Panel **Ia** shows a significant difference in REM-stage NPV, which was higher in studies using forehead electrodes. **II** *Electrode type*: Panels **IIa** and **IIb** show significantly higher N2 PPV and REM NPV in studies using dry electrodes. **III** *Scoring method*: Deep learning outperformed machine learning in macro-averaged *κ*, F1, and MCC, as well as N1-stage *κ*, F1, and MCC and N2 F1. Manual scoring showed significantly better performance than machine learning in macro-averaged F1 and several Wake- and REM-stage metrics. Proprietary algorithms yielded significantly higher performance than machine learning in Wake-stage PPV, N2-stage NPV, N3-stage SP, and REM-stage NPV. **IV** *Number of electrodes***:** Pearson correlation analysis revealed significant positive associations between the number of recording electrodes and N3-stage performance metrics, including *κ*, SE, MCC, and F1. A significant negative correlation was observed for REM-stage NPV. ACC: accuracy; *κ*: Cohen’s kappa; MCC: Matthews correlation coefficient; SE: sensitivity; SP: specificity; PPV: positive predictive value; NPV: negative predictive value; F1: F1 score.

Device characteristics had mixed effects on wEEG performance, with few statistically significant group differences. No significant differences were found between commercial and prototype devices (Supplementary Fig. S5, Table S7). The only significant effect of electrode placement was higher REM-stage NPV for forehead-based systems (Fig. 7I). Dry electrodes showed significantly higher N2 PPV and N3 NPV than wet electrodes (Fig. 7II). The scoring method had the most consistent impact: machine learning was significantly outperformed by deep learning (macro *κ*, F1, MCC; N1 *κ*, F1, MCC; N2 F1) and by manual scoring (macro F1; N1 *κ*, F1, MCC; REM ACC, SP). Proprietary algorithms also outperformed machine learning in Wake PPV, N2 NPV, N3 SP, and REM NPV (Fig. 7III), though the small number of studies (*n* = 5) limits interpretation. Importantly, in the five studies^22,27,31,34,41^ that evaluated multiple scoring methods on the same data, manual scoring consistently outperformed automatic scoring, and deep learning outperformed classical machine learning (Table 1). Electrode count showed significant positive correlations with N3 *κ*, SE, MCC, and F1, supporting the value of increased spatial coverage for slow-wave sleep detection, while REM NPV showed a weaker but significant negative correlation (Fig. 7IV). In summary, while most device features had limited impact, electrode count and scoring method significantly influenced N3 and REM-stage performance (Supplementary Figs. S6–S8; Tables S8–S10).

### Sensitivity analysis

To assess robustness, a leave-one-out sensitivity analysis was conducted across all 36 results from 32 studies, classifying five stages. No individual study exceeded the pre-defined ±0.03 threshold for altering pooled values; the largest absolute shifts were ≤0.012 and relative changes stayed under 3.5%. The most sensitive metrics were in the N1 stage (*κ*, SE, F1, and MCC), where omitting the most high-performing studies (e.g., Li et al.^27^) slightly raised scores. Notably, Mikkelsen et al.^53^ appeared most frequently as the study with the highest influence across multiple metrics, particularly in overall and REM-stage results. Thus, the meta-analytic results can be considered robust when aggregating across all five-stage studies (Supplementary Fig. S9, Supplementary Table S11).

Subgroup-specific analyses revealed less robustness, with some comparisons highly sensitive to individual studies. The N1 stage was most affected, reflecting the inherent difficulty of classifying this stage and highlighting its susceptibility to methodological and sample-related variation. Metrics emphasizing class imbalance and agreement (*κ*, F1, MCC, PPV) fluctuated more than overall accuracy. Instability was concentrated in subgroups with few studies—especially clinical (*n* = 5), mixed-population (*n* = 7), home-based (*n* = 10), and proprietary-algorithm (*n* = 5) groups. In contrast, estimates from healthy cohorts, controlled lab settings, and devices using forehead electrode placement or dry electrodes were robust. A few studies—particularly Ravindran et al.^28^, Li et al.^27^, and Borges et al.^44^—consistently drove subgroup results: excluding Ravindran et al.^28^ raised averages, while omitting Li et al.^27^ or Borges et al.^44^ lowered them (Supplementary Table S12). In summary, while aggregated findings are reliable, differences between methodological groups should be interpreted with caution, as they are often driven by just one or two influential studies.

## Discussion

The rising popularity of wEEGs underscores the need for a standardized assessment of their ability to classify individual stages. Misclassifications can compromise sleep architecture analysis, and, consequently, affect therapeutic decisions and sleep quality evaluations. Both five- and four-stage studies revealed variability in wEEGs’ performance across studies. Our analysis identified the sleep stages where wEEGs’ classifications were most and least aligned with PSG.

Overall performance results from 32 studies classifying five sleep stages showed clear discrepancies between overall accuracy and more balanced metrics like MCC and *κ*, highlighting limitations in wEEGs’ ability to classify individual stages. These discrepancies were amplified by differences between micro- and macro-averaged metrics. Micro-averaging, which weighs each epoch equally, inflated SE, PPV, and F1 due to the dominance of the N2 stage, but masked underperformance in less frequent stages such as N1. In contrast, macro-averaging, which gives equal weight to each class, yielded a higher ACC by amplifying true negative counts in less frequent stages like N1. These findings underscore the need to prioritize balanced evaluation metrics when validating wEEG performance to account for class imbalances inherent in sleep stage distributions. The Wake stage showed the highest classification accuracy, with excellent agreement to PSG despite slight underestimation in duration. This confirms that wake-sleep differentiation remains a key strength of wEEGs. N1, the least frequent stage, emerged as the most challenging to classify, reflecting its transitional nature and less distinct EEG characteristics. Significant variability in its detection reliability across studies, shown by the largest standard deviations in various metrics and the greatest estimation variability in the Bland-Altman analysis, highlights the need for improved automatic detection of this stage by wEEGs. The most dominant N2, N2 stage yielded high SE, but significantly lower scores in balanced metrics MCC and *κ*, indicating frequent inclusion of N2 epochs, but not necessarily correct exclusion of others. These observations highlight concerns regarding over-reliance on SE, the most frequently reported metric for stage-specific performance, which potentially overlooks N2 classification challenges. N3 emerged as the most reliably detected stage. It consistently yielded high balanced scores with minimal inter-study variability and strong agreement in both duration and proportion, supporting its robust detection by wEEGs. REM sleep was also generally well classified but displayed higher variability across studies. This, along with slight overestimation in duration, indicates a need for further refinement, especially given the clinical importance of REM-related sleep disorders. Importantly, the sensitivity analysis confirmed that no single study significantly altered the pooled results, confirming the robustness of the overall meta-analytic findings.

When examining the top studies for overall and stage-specific MCC in the five-stage classification, several consistently stood out. Li et al. reported strong results using the WPSG-I headband with 6 dry electrodes in a controlled setting with 20 mixed (healthy and clinical) participants. Both manual and proprietary algorithmic scoring were evaluated, with manual scoring performing particularly well, though automatic results were also among the best. Borup et al.^46^ achieved high MCCs using a dry in-ear prototype^72^ in a home setting with 20 healthy participants over four nights, applying a personalized deep learning model. Borges et al.^44^ achieved comparable results using a wet in-ear prototype^74,75^ on mostly OSA participants in a lab setting, with automatic scoring. While the results suggest strong device performance, they likely reflect not only device quality but also study design factors.

Several methodological variables significantly influenced wEEG performance across studies. While electrode placements (forehead or ear) and types (dry or wet) showed limited impact, the scoring method and electrode count consistently influenced stage-specific outcomes. Manual and deep learning-based scoring generally outperformed machine learning, highlighting the importance of algorithm complexity and human oversight, especially for difficult stages like N1. Electrode count was positively associated with N3 performance, reinforcing the importance of spatial resolution for detecting slow-wave sleep. Participant health status and recording environment also affected performance, though these findings should be interpreted cautiously given the limited sample sizes in some subgroups. While these results underscore the importance of methodological context when evaluating wEEG systems, many subgroup-level differences were highly sensitive to the inclusion or exclusion of just one or two studies. This was particularly true for underrepresented methodological groups, such as those involving clinical or mixed populations, home-based recordings, or deep learning and proprietary algorithms, where few studies were available. As a result, differences between these subgroups should be interpreted with caution, as they may reflect study-specific effects rather than consistent, generalizable patterns.

A smaller subset of studies reported results using a four-stage classification system, and although limited in number (*n* = 7), they offered additional insights that in part mirrored the five-stage findings. As with five-stage studies, ACC tended to overestimate performance relative to balanced metrics such as MCC and *κ*, again pointing to inconsistencies in individual stage classification. Differences between micro- and macro-averaged ACC were also present, though performance across SE, PPV, and F1 was more uniform, suggesting less skew from dominant stages. Wake stage showed substantial variability across studies, with the lowest SE but high NPV, and relatively strong agreement in duration and proportion, indicating wEEGs effectively detected its absence but often missed its presence. Light Sleep mirrored challenges seen with N2, with low MCC and *κ*, signaling misclassification issues. Deep Sleep was the most reliably detected, echoing five-stage results, with high MCC and minimal bias in proportion estimates. REM was generally well classified, though lower MCC and overestimation in duration again highlighted areas for refinement.

The significant variability in stage-specific performance across studies highlights the need for a consistent and robust approach to evaluating wEEG-based sleep staging. Commonly used metrics such as overall ACC, SP, and NPV tend to overstate performance, largely due to the disproportionate influence of TNs in class-imbalanced data. This was evident in our analysis, where these metrics consistently outperformed more balanced alternatives like MCC across both five- and four-stage classification systems. To ensure comparability and reliability in future research, we propose a standardized framework for validating wEEG systems against PSG:

1. *Use of balanced metrics (MCC or κ)*: For both overall and stage-specific evaluation, we recommend MCC and *κ*. Unlike overall accuracy, which can be misleading in imbalanced datasets, these metrics consider all elements of the confusion matrix and remain informative even when some stages (e.g., N1 or Wake) are underrepresented. MCC, in particular, offers a single, interpretable value that reflects the overall quality of classification and is increasingly recommended for imbalanced settings^76^. Stage-specific evaluation should rely on per-class MCC or *κ*, which reflect true classification quality even for minority stages like N1, and be complemented by sensitivity (SE) to highlight detection ability.
2. *Reporting confusion matrices* detailing labels from wEEGs and manually scored PSG should be included for detailed analysis.
3. *Adopting standardized staging systems*, classifying sleep into Wake, N1, N2, N3 and REM or Wake, Light Sleep, Deep Sleep and REM. Three studies in this meta-analysis did not employ five- or four-stage classification systems, making them incomparable to other studies.

This meta-analysis, while comprehensive, faced certain limitations. First, potential language and publication bias may have influenced the included studies, as only English-language, peer-reviewed publications were considered. This may have excluded relevant non-English data, potentially skewing results. Additionally, four analyzed studies did not employ five- or four-stage classification systems, making them incomparable to others. Moreover, most studies included in this meta-analysis focused on participants in their mid-20s to mid-30s, leading to a notable age bias. This overrepresentation limits the generalizability of findings, particularly for older adults who experience age-related changes in sleep architecture, such as reduced N3 and REM sleep, increased fragmentation, and altered EEG signal patterns, that may affect wEEG accuracy. Clinical populations were also underrepresented. Most studies were conducted on healthy participants, despite the increasing interest in applying wEEG systems to monitor or diagnose sleep disorders. Altered sleep architecture, comorbidities, and medication use and atypical sleep patterns may alter EEG signals and algorithm performance in ways not captured by current validation studies.

Future research directions should focus on expanding the scope of wEEG validation studies. Dedicated studies involving both older adults and clinical cohorts are critical to establishing the real-world reliability and clinical applicability of wEEG systems. Longitudinal and ambulatory studies capturing night-to-night variability and real-life usage conditions would improve ecological validity. Studies should also report on device wearability and user adherence, especially for home-based applications, to assess long-term feasibility. In addition to staging accuracy, future work should also assess wEEG reliability in estimating other sleep parameters—such as sleep onset latency and wake after sleep onset. Comparative analyses examining the impact of specific electrode placement in different device types on sleep stage classification can yield critical insights into optimizing wEEGs. Incorporating alternative technologies like Photoplethysmography (PPG) or actigraphy can also enhance the breadth of comparisons across different sleep staging technologies. Ethical considerations, including data privacy and responsible handling of sleep health data, must also be addressed as wEEG use expands beyond research settings. Crucially, adherence to the standardized framework proposed in this analysis is fundamental for future research. Embracing this framework will aid in systematically assessing and refining wEEGs’ capabilities and limitations in classifying sleep stages.

The increased use of wEEGs in sleep monitoring presents significant potential in both clinical and consumer health. The analysis of 43 validation studies revealed a substantial overall agreement between wEEGs and PSG. However, their performance in accurately classifying specific sleep stages varied considerably. The N1 stage presented significant classification challenges, while the N3 or Deep Sleep stage was the most reliably identified stage. These discrepancies are critical, as misclassifications can lead to incorrect sleep architecture analysis, directly impacting clinical decisions and patient care. Furthermore, manual scoring generally outperformed automatic methods, particularly for the N1 stage, highlighting the need for improved algorithms. Additionally, electrode count was positively associated with N3 detection, suggesting that spatial resolution remains an important factor for optimizing wEEG performance. To address the variabilities in stage-specific performance, we propose a standardized evaluation framework emphasizing balanced metrics like MCC and *κ*, and consistency in reporting validation results. This approach aims to facilitate advancements in wEEG technology by enhancing comparisons across studies and devices. As wEEGs become more integrated into home-based sleep monitoring, ensuring their reliability becomes crucial for clinical adoption. This meta-analysis paves the way for future research to refine wEEGs, particularly for stages with less distinct features like N1, ensuring their reliability across diverse populations and settings. This is essential to fully realize their potential in enhancing sleep monitoring and analysis in both clinical and consumer applications.

## Methods

### Search strategy

A systematic literature search was conducted across four databases: PubMed, Scopus, IEEE Xplore, and Web of Science. The objective was to identify studies published within the past 10 years that investigated the use of wearable EEG devices for sleep staging in human subjects and were validated against PSG. The search strategy included the following keywords and Boolean operators: “sleep AND (wearable OR mobile OR headband) AND (EEG OR electroencephalogram OR electroencephalography)”. The search was limited to English-language publications. Additionally, the reference lists of all included articles were manually screened to identify any further relevant studies.

### Study selection

**Fig. 8:**
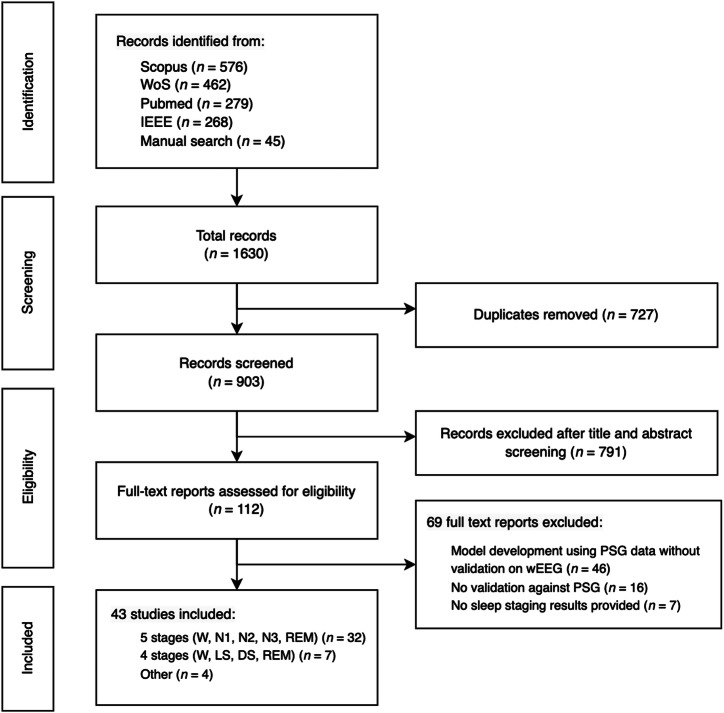
PRISMA diagram. Diagram illustrating the systematic search and selection process in this meta-analysis. Initially, 1630 records were identified (1585 from databases, 45 from other sources). After removing duplicates, 903 records were screened based on title and abstract, leading to the exclusion of 791 records. Full-text assessment of 112 articles resulted in the exclusion of 69 for the following reasons: 44 studies developed models using PSG data but did not validate them on a wearable EEG device, 16 did not include validation against PSG, and 7 did not report any sleep staging results. Ultimately, 43 studies were included, categorized into three groups: 32 studies classifying Wake, N1, N2, N3, REM; 7 studies classifying Wake, Light, Deep, REM; and 4 studies using other classification systems.

A total of 1585 records were identified through database searching, and 45 additional records through manual searches of reference lists and websites of wEEGs. After removing duplicates, 903 records remained for the title and abstract screening. Studies were included if they met the following inclusion criteria: conducted within the last 10 years, conducted on human subjects, published in English, used wEEG for sleep staging, and compared the results to a gold standard such as PSG. Studies were excluded if they were reviews or conducted on non-human subjects. After the application of these criteria, 791 records were excluded. The full texts of the remaining 112 studies were assessed for eligibility. Of these, 69 studies were excluded because they either lacked validation on a wearable EEG device, did not include validation against PSG, or did not report sleep staging results. This resulted in the inclusion of 43 validation studies in the final analysis (Fig. 8).

### Data extraction

Extracted information covered study design and device characteristics, including the device name, number of recording electrodes, their placements, and types (wet or dry). Participant-related data included the number of participants, age, and population type (e.g., healthy individuals or clinical populations). Additional details such as the study environment (e.g., home or controlled setting), number of recorded nights per participant, and total number of analyzed epochs were also recorded. For each study, the sleep staging method used by the wEEG (e.g., manual scoring, machine learning, or deep learning), the validation approach (e.g., comparison against PSG scored by an expert), and the sleep stage classification scheme (e.g., five-stage: Wake, N1, N2, N3, REM; or four-stage: Wake, Light, Deep, REM) were documented. Confusion matrices and reported evaluation metrics were collected as the primary data for quantitative analysis. When confusion matrices were not explicitly provided, they were reconstructed based on available information. All confusion matrices and details on their derivation are provided in the Supplementary Materials.

### Data analysis

1 $${\kappa }_{{\rm{m}}{\rm{u}}{\rm{l}}{\rm{t}}{\rm{i}}{\rm{c}}{\rm{l}}{\rm{a}}{\rm{s}}{\rm{s}}}=\frac{{p}_{j}-{p}_{e}}{1-{p}_{e}}$$

where

2 $${p}_{j\,({\rm{m}}{\rm{u}}{\rm{l}}{\rm{t}}{\rm{i}}{\rm{c}}{\rm{l}}{\rm{a}}{\rm{s}}{\rm{s}})}=\frac{{\sum }_{i=1}^{n}{C}_{ii}}{N}$$

and

3 $${P}_{e\,(\mathrm{multiclass})}=\mathop{\sum }\limits_{i=1}^{n}\left(\frac{{C}_{i+}{\times C}_{+i}}{{N}^{2}}\right)$$

with

- *C:* confusion matrix.
- *C*_*ii*_: the diagonal elements (true positives for each class).
- *n:* the number of classes.
- *N:* total number of epochs (sum of all elements in the confusion matrix).
- *C*_*i+*_: sum of elements in row *i* (true instances of class *i*).
- *C*_*+i*_: *:* sum of elements in column *i* (predicted instances of class *i*).

(1) The confusion matrices were used to derive true positives (TP), true negatives (TN), false positives (FP), and false negatives (FN). Using these values, the overall agreement between the wEEG and the reference standard was evaluated with Cohen’s Kappa (*κ*), which is calculated as shown in Eq. (1). *Κ* values are interpreted as follows: <0.0 poor, 0.00–0.20 slight, 0.21–0.40 fair, 0.41–0.60 moderate, 0.61–0.80 substantial, and over 0.80 excellent agreement^77^.

To provide a balanced performance measure that accommodates variations in class sizes, the multiclass Matthews correlation coefficient (MCC) was computed using Eq. (4); MCC scores range from 1 (indicating total disagreement), through 0 (random prediction), to +1 (perfect prediction).

4 $${{{MCC}}}_{multiclass}=\frac{{\sum }_{k}{\sum }_{l}{\sum }_{m}{V}_{klm}-{\sum }_{i}{\sum }_{j}({X}_{ij}^{2})}{\root{2}\of{{\chi }^{2}+{\sum }_{i}({X}_{i+}^{2})\times {\sum }_{i}({X}_{j+}^{2})}}$$

where

5 $${V}_{klm}={C}_{ik}{C}_{il}{C}_{+j}{C}_{jm}\,for\,k\ne 1\,{\rm{a}}{\rm{n}}{\rm{d}}1\ne m$$

and

6 $${Xij}={C}_{{ij}}{C}_{+j}-{C}_{j+}{C}_{i+}$$

with

- *C:* confusion matrix.
- *C*_*ii*_: the diagonal elements (true positives for each class).
- *n:* the number of classes.
- *N:* total number of epochs (sum of all elements in the confusion matrix).
- *C*_*i+*_: sum of elements in row *i* (true instances of class *i*).
- *C*_*+i*_: *:* sum of elements in column *i* (predicted instances of class *i*).
- *k, l, m, i, j* are classes.
- *C*_*ij*_: number of observations known to be in group i but predicted to be in group *j*.
- *χ*^2^*:* is the chi-squared statistic relative to the confusion matrix.

For sleep-stage-specific evaluation, the following metrics were computed: ACC (Eq. (7)), *κ* (Eq. (8)), sensitivity (SE) (Eq. (11)), specificity (SP) (Eq. (12)), positive predictive value (PPV) (Eq. (13)), negative predictive value (NPV) (Eq. (14)), F1 score (Eq. (15)), and MCC (Eq. (16)).

7 $${\rm{ACC}}=\frac{{\rm{TN}}+{\rm{TP}}}{{\rm{TN}}+{\rm{TP}}+{\rm{FN}}+{\rm{FP}}}$$

8 $${\kappa }=\frac{{p}_{j}-{p}_{e}}{1-{p}_{e}}$$

where

9 $${p}_{j}=\frac{TN+TP}{N}$$

and

10 $${p}_{e}=\frac{({\rm{t}}{\rm{r}}{\rm{u}}{\rm{e}}\,{\rm{Y}}{\rm{E}}{\rm{S}}\times {\rm{p}}{\rm{r}}{\rm{e}}{\rm{d}}{\rm{i}}{\rm{c}}{\rm{t}}{\rm{e}}{\rm{d}}\,{\rm{Y}}{\rm{E}}{\rm{S}})+({\rm{t}}{\rm{r}}{\rm{u}}{\rm{e}}\,{\rm{N}}{\rm{O}}\times {\rm{p}}{\rm{r}}{\rm{e}}{\rm{d}}{\rm{i}}{\rm{c}}{\rm{t}}{\rm{e}}{\rm{d}}\,{\rm{N}}{\rm{O}})}{{N}^{2}}$$

with

- true YES**:** number of times the ground truth identifies a particular class (row total in the confusion matrix).
- predicted YES**:** number of times the device identifies a particular class (column total in confusion matrix).
- *N*: total number of epochs (sum of all elements in the confusion matrix)
  11 $${\rm{SE}}=\frac{{\rm{TP}}}{{\rm{TP}}+{\rm{FN}}}$$
  12 $${\rm{SP}}=\frac{{\rm{TN}}}{{\rm{TN}}+{\rm{FP}}}$$
  13 $${\rm{PPV}}=\frac{{\rm{TP}}}{{\rm{TP}}+{\rm{FP}}}$$
  14 $${\rm{NPV}}=\frac{{\rm{TN}}}{{\rm{TN}}+{\rm{FN}}}$$
  15 $${\rm{F}}1=\frac{2\times {\rm{SE}}\times {\rm{PPV}}}{{\rm{SE}}+{\rm{PPV}}}$$
  16 $${\rm{MCC}}=\frac{{\rm{TP}}\times {\rm{TN}}-{\rm{FP}}\times {\rm{FN}}}{\sqrt{({\rm{TP}}+{\rm{FP}})({\rm{TP}}+{\rm{FN}})({\rm{TN}}+{\rm{FP}})({\rm{TN}}+{\rm{FN}})}}$$

Studies were categorized based on their use of either a five-stage classification system (i.e., N1, N2, N3, REM, Wake) or a four-stage system (e.g., Wake, Light, Deep, REM). In multiclass classifications, the above evaluation metrics are often averaged to reflect overall device performance. Micro-averaged metrics, which give equal weight to each epoch, were calculated by aggregating the counts of TPs, FPs, TNs, and FNs (Eqs. (17)–(22)).

17 $${\rm{A}}{\rm{C}}{\rm{C}}=\frac{{\sum }_{i=1}^{n}{C}_{ii}}{N}$$

where

- *C:* confusion matrix.
- *C*_*ii*_: the diagonal elements (true positives for each class).
- *n:* the number of classes.
- *N:* total number of epochs (sum of all elements in the confusion matrix).
  18 $${SE}_{micro}=\frac{\sum {\rm{T}}{\rm{P}}}{\sum {\rm{T}}{\rm{P}}+\sum {\rm{F}}{\rm{N}}}$$
  19 $${SP}_{micro}=\frac{\sum {\rm{T}}{\rm{N}}}{\sum {\rm{T}}{\rm{N}}+\sum {\rm{F}}{\rm{P}}}$$
  20 $${PPV}_{micro}=\frac{\sum {\rm{T}}{\rm{P}}}{\sum {\rm{T}}{\rm{N}}+\sum {\rm{F}}{\rm{P}}}$$
  21 $${NPV}_{micro}=\frac{\sum {\rm{T}}{\rm{N}}}{\sum {\rm{T}}{\rm{N}}+\sum {\rm{F}}{\rm{N}}}$$
  22 $${{F}{1}}_{micro}=\frac{2\times {{\rm{S}}{\rm{E}}}_{{\rm{m}}{\rm{i}}{\rm{c}}{\rm{r}}{\rm{o}}}\times {{\rm{P}}{\rm{P}}{\rm{V}}}_{{\rm{m}}{\rm{i}}{\rm{c}}{\rm{r}}{\rm{o}}}}{{{\rm{S}}{\rm{E}}}_{{\rm{m}}{\rm{i}}{\rm{c}}{\rm{r}}{\rm{o}}}+{{\rm{P}}{\rm{P}}{\rm{V}}}_{{\rm{m}}{\rm{i}}{\rm{c}}{\rm{r}}{\rm{o}}}}$$

Macro-averaged metrics, which assign equal importance to each class, were calculated by averaging the metrics computed for each stage (Eqs. (29)–(29)).

23 $${{A}{C}{C}}_{macro}=\frac{{ACC}_{1}+{ACC}_{2}+\ldots +{{A}{C}{C}}_{n}}{n}$$

24 $${\kappa }_{macro}=\frac{{\kappa }_{1}+{\kappa }_{2}+\ldots +{\kappa }_{n}}{n}$$

25 $${{S}{E}}_{macro}=\frac{{{\rm{S}}{\rm{E}}}_{1}+{{\rm{S}}{\rm{E}}}_{2}+\ldots +{{\rm{S}}{\rm{E}}}_{n}}{n}$$

26 $${{S}{P}}_{macro}=\frac{{{\rm{S}}{\rm{P}}}_{1}+{{\rm{S}}{\rm{P}}}_{2}+\ldots +{{\rm{S}}{\rm{P}}}_{n}}{n}$$

27 $${{N}{P}{V}}_{macro}=\frac{{{\rm{N}}{\rm{P}}{\rm{V}}}_{1}+{{\rm{N}}{\rm{P}}{\rm{V}}}_{2}+\ldots +{{\rm{N}}{\rm{P}}{\rm{V}}}_{n}}{n}$$

28 $${{F}{1}}_{macro}=\frac{{{\rm{F}}1}_{1}+{{\rm{F}}1}_{2}+\ldots +{{\rm{F}}1}_{n}}{n}$$

29 $${{M}{C}{C}}_{macro}=\frac{{{\rm{M}}{\rm{C}}{\rm{C}}}_{1}+{{\rm{M}}{\rm{C}}{\rm{C}}}_{2}+\ldots +{{\rm{M}}{\rm{C}}{\rm{C}}}_{n}}{n}$$

For each sleep stage and overall performance, the evaluation metrics were used to compute the mean and standard deviation across studies. When confusion matrices were available, the metrics were computed directly from these; otherwise, reported metrics from the original publications were used. The results of studies classifying five stages and four stages were analyzed separately, as each device’s scoring model was developed and validated for a specific staging structure; reclassification would not reflect intended device behavior or validation protocols.

(2) Significant differences between MCC and all other metrics for both, overall and sleep-stage-specific metrics were analyzed using the Mann–Whitney *U*-test, which is suitable for small sample sizes and non-normally distributed data.

(3) Bland–Altman plots were analyzed for differences in stage durations and proportions between device and ground truth scorings.

(4) Studies classifying five sleep stages were stratified by device characteristics like electrode placement (e.g. forehead or ear), electrode type, whether wEEG was a prototype or commercially available, participants’ health status, study environment, and device sleep staging method. The Mann-Whitney U-test was employed to highlight significant differences in evaluation metrics between the groups.

(5) Relationships between evaluation metrics and the number of epochs and participants were explored using the Pearson correlation in studies classifying five sleep stages.

(6) To evaluate the robustness of pooled performance estimates, a leave-one-out (LOO) sensitivity analysis was conducted on all studies using five-stage sleep classification. For each evaluation metric and sleep stage, pooled values were recalculated iteratively by omitting one study at a time, and the maximum absolute (|Δ|) and relative (Δ %) changes were recorded. Because all performance metrics are scaled 0–1, we considered an absolute shift of |Δ| > 0.03 (i.e., ≥3 percentage points) to indicate a potentially influential study. This analysis was also repeated within each subgroup defined by participant health status, study environment, electrode placement, electrode type, device type (prototype vs. commercial), and scoring method, to assess the stability of results within stratified comparisons.

All data analyses were conducted in Python.

## Supplementary information

Supplementary Information

## Data Availability

No datasets were generated or analysed during the current study.
