# Supplementary material for: Evaluating the performance of wearable EEG sleep monitoring devices: a meta-analysis approach
Source: NPJ Biomed Innov. 2025 Oct 9;2:33. doi: 10.1038/s44385-025-00034-w (PMC13055050; doi:10.1038/s44385-025-00034-w)
Supplement: Supplementary file 1 — Supplementary Information [file 44385_2025_34_MOESM1_ESM.pdf]

Supplementary figures

a) Wake

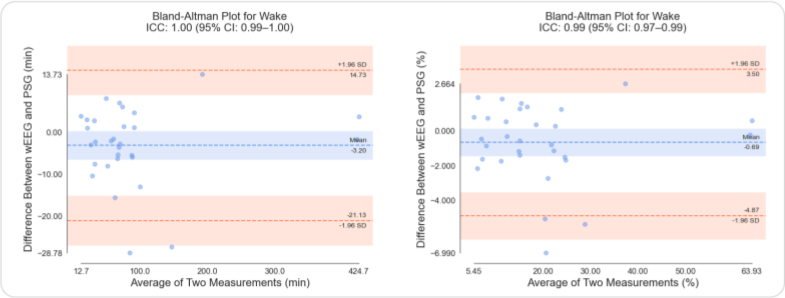

b) N1

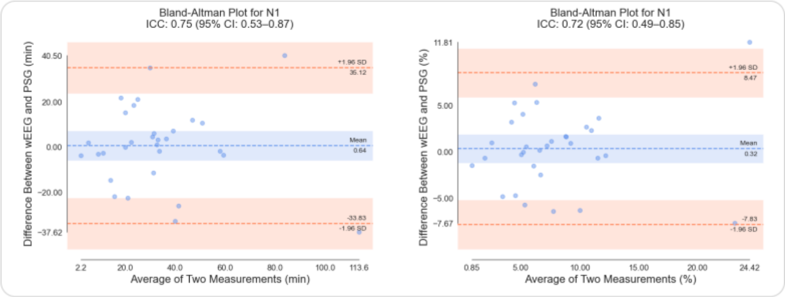

b) N2

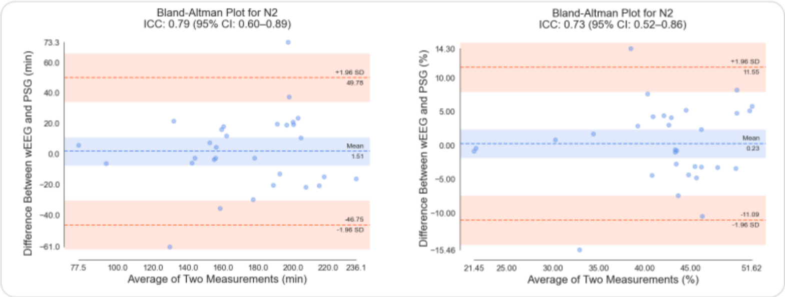

c) N3

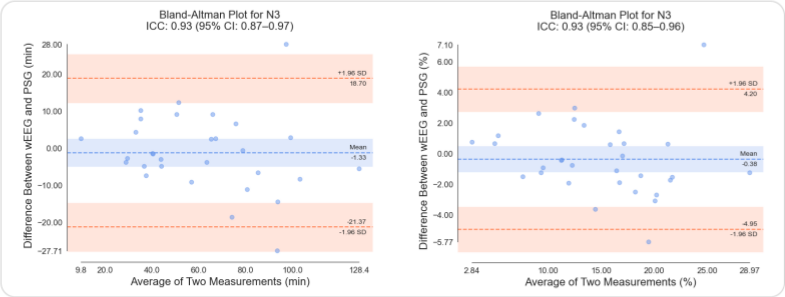

d) REM

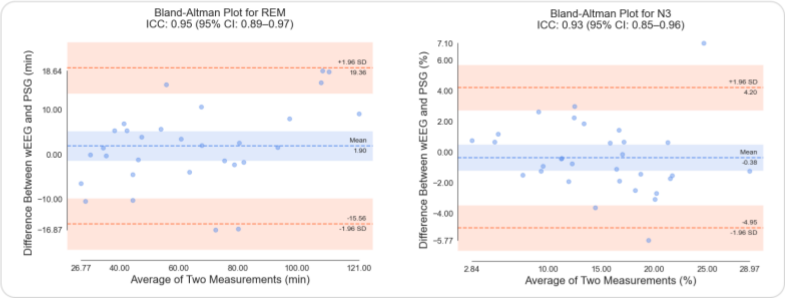

Supplementary figure S1. Comparative Bland-Altman analysis of sleep stage durations and proportions as measured by wearable EEG (wEEG) versus polysomnography (PSG) in studies classifying 5 stages. Each panel shows the difference between wEEG and PSG measurements against their mean, with the mean bias (blue

dashed line) and limits of agreement (mean ±1.96 SD, red dashed lines) shaded in color. Intraclass correlation coefficients (ICCs) with 95% confidence intervals are reported for each stage. **a) Wake:** wEEGs slightly underestimated Wake duration (mean: –3.20 min; LoA: –21.13 to +14.73 min) and proportion (–0.69%; LoA: –4.67 to +3.30%). Agreement was excellent, with ICCs of 1.00 (minutes) and 0.99 (proportion), indicating near-perfect reliability. **b) N1:** Duration was marginally overestimated on average (+0.64 min; LoA: –33.83 to +35.12 min), and proportion slightly overestimated (+0.32%; LoA: –7.83 to +8.47%). ICCs were 0.75 and 0.72, indicating moderate-to-good agreement. **c) N2:** wEEGs slightly overestimated N2 duration (+1.51 min; LoA: –46.75 to +49.78 min) and proportion (+0.23%; LoA: –11.09 to +11.55%). ICCs were 0.79 and 0.73, reflecting good agreement. **d) N3:** Mean difference in N3 duration was +1.93 min (LoA: –21.37 to +25.70 min), and proportion +0.38% (LoA: –4.95 to +4.20%). ICCs were both 0.93, reflecting excellent agreement. **e) REM:** REM sleep was slightly overestimated in duration (+1.90 min; LoA: –15.56 to +19.36 min) and proportion (+0.36%; LoA: –4.95 to +4.20%). ICCs were 0.95 and 0.93, again indicating excellent

a) Wake

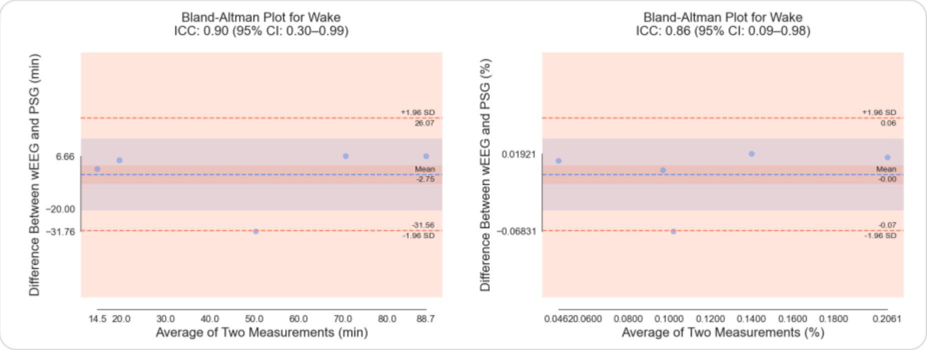

b) Light Sleep

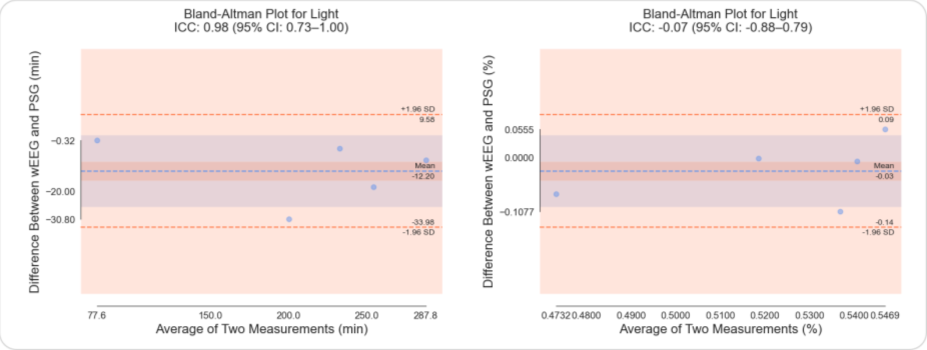

b) Deep Sleep

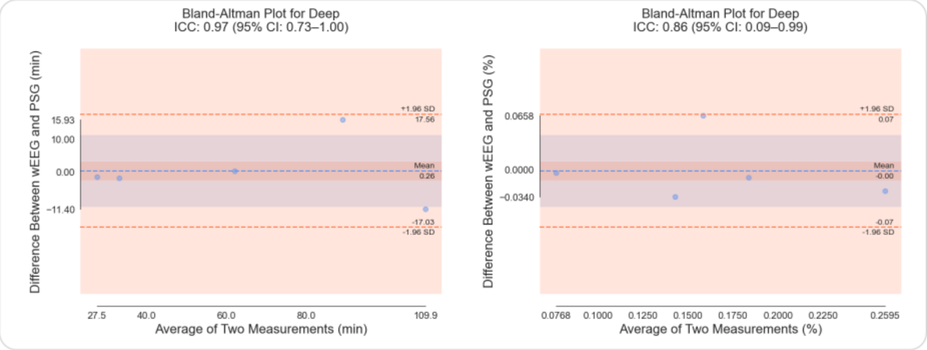

c) REM

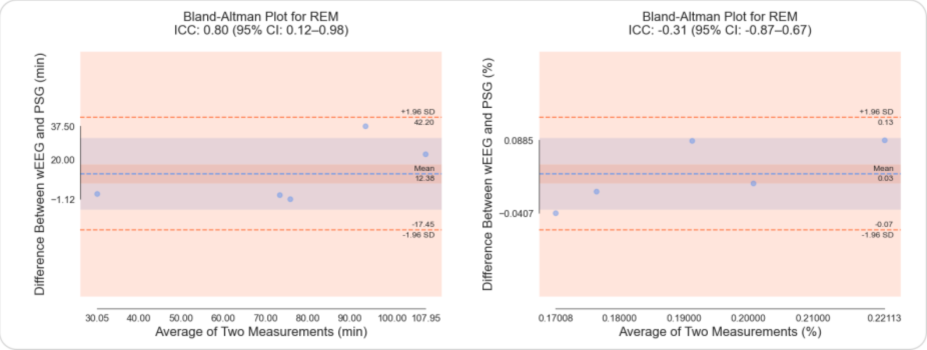

Supplementary figure S2. **Bland–Altman plots comparing sleep stage durations and proportions between wearable EEG (wEEG) and polysomnography (PSG) in studies using 4-stage classification.** Each panel shows the difference between wEEG and PSG measurements against their mean, with the mean bias (blue dashed line) and limits of agreement (mean ±1.96 SD, red dashed lines) shaded in color. Intraclass correlation coefficients (ICCs) with 95% confidence intervals are reported for each stage. **a) Wake:** wEEGs slightly overestimated Wake duration (mean: +2.75 min; LoA: –31.56 to +26.07 min) and proportion (mean: +0.01%; LoA: –0.07 to +0.07%). ICCs were 0.90 (minutes) and 0.86 (proportion), indicating excellent agreement on duration and good agreement on proportion. **b) Light Sleep:** There was a small underestimation of Light sleep duration (mean: –1.22 min; LoA: –33.39 to +30.95 min), and a near-zero mean difference in proportion (–0.03%; LoA: –0.14 to +0.09%). The ICC

for duration was excellent (0.98), while proportion agreement was poor (ICC = –0.07), suggesting inconsistency in proportional estimates. **c) Deep Sleep:** wEEGs slightly overestimated Deep Sleep (mean: +0.28 min; LoA: –17.03 to +17.56 min) and showed minimal bias in proportion (–0.01%; LoA: –0.07 to +0.07%). ICCs for both duration and proportion were strong (0.97 and 0.86), indicating excellent-to-good agreement. **d) REM:** WDs overestimated REM duration (mean: +1.28 min; LoA: –17.45 to +20.02 min) and proportion (+0.03%; LoA: –0.04 to +0.13%). Agreement on duration was good (ICC = 0.80), but poor on proportion (ICC = –0.31), suggesting unreliable percentage estimates.

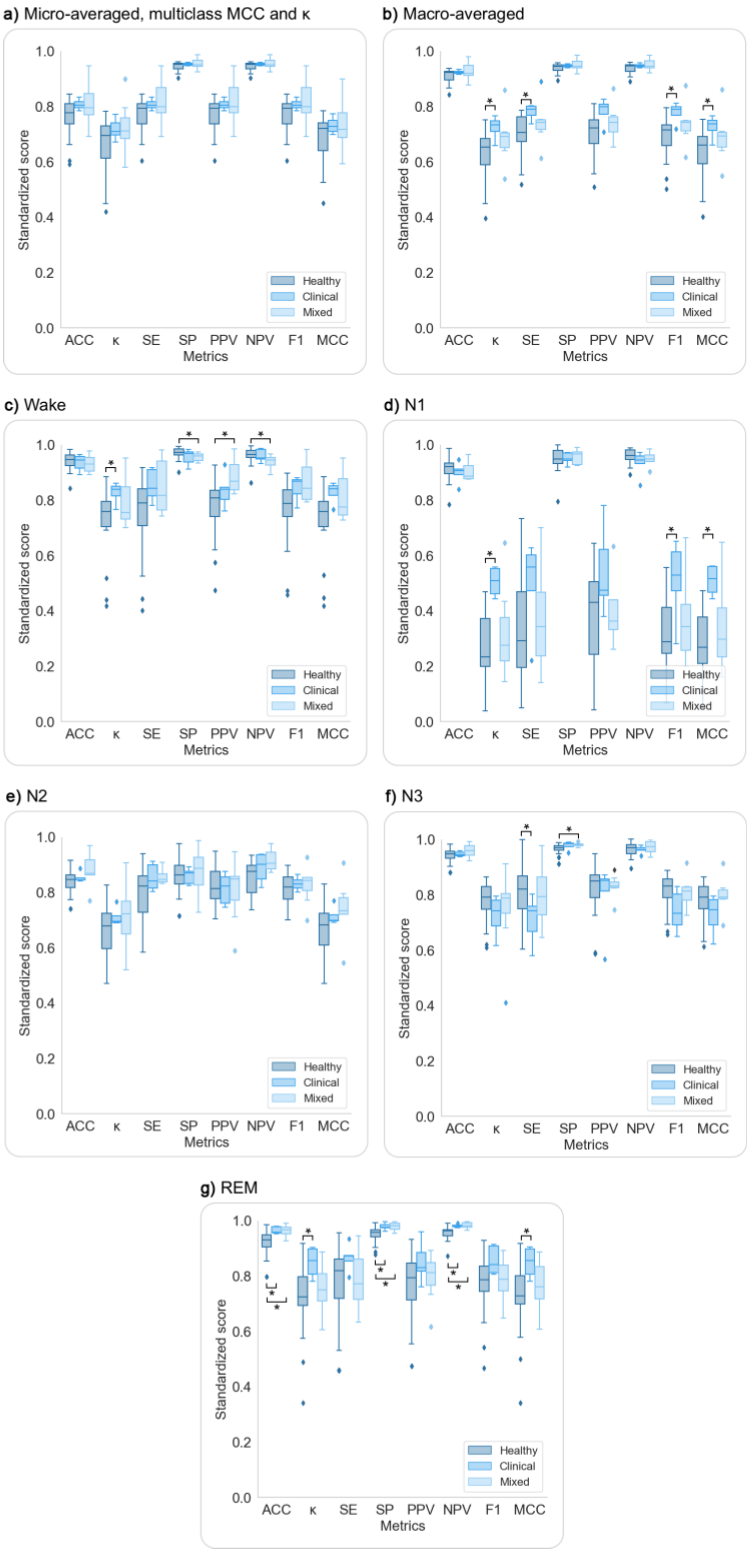

Supplementary figure S3. Influence of population type on wEEG evaluation metrics. This figure compares performance metrics across studies including healthy (light blue), clinical (medium blue), and mixed (dark blue) participant populations. Panels show results for a) micro-averaged metrics and multiclass κ and MCC, b) macro-averaged metrics, and stage-specific metrics for c) Wake, d) N1, e) N2, f) N3, and g) REM. Significant group differences (Mann–Whitney U test, $p < 0.05$) are indicated with asterisks. Clinical and mixed-population studies generally reported higher performance than those including only healthy participants, especially for macro-averaged κ, SE, F1, and MCC, as well as several stage-specific metrics in Wake, N1, N3, and REM. ACC: accuracy; κ: Cohen's kappa; MCC: Matthews correlation coefficient; SE: sensitivity; SP: specificity; PPV: positive predictive value; NPV: negative predictive value.

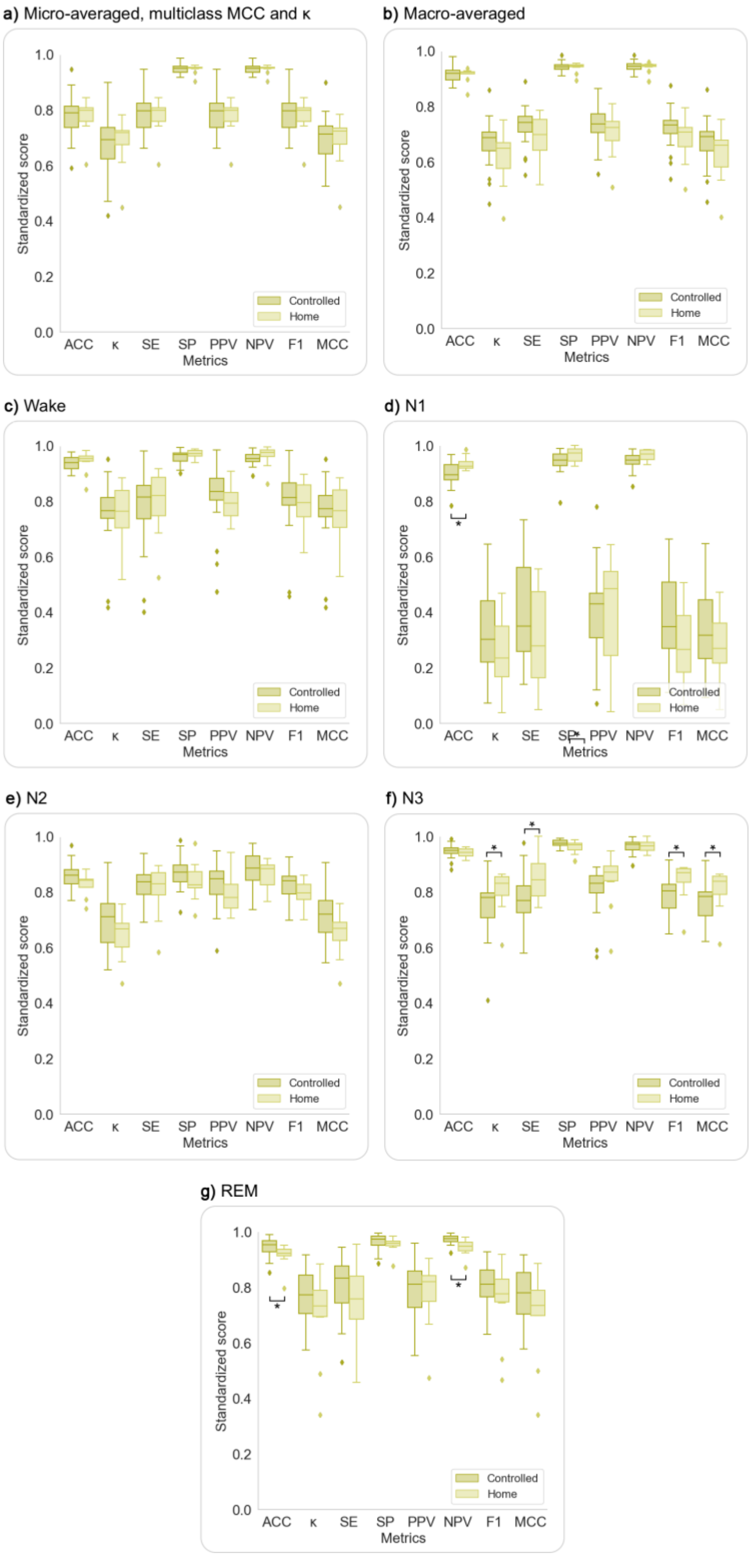

Supplementary figure S4. Influence of study environment on wEEG evaluation metrics. Boxplots compare standardized scores for studies conducted in controlled environments (e.g., sleep lab/hospital, light yellow) versus home settings (dark yellow) across a) micro-averaged and multiclass metrics, b) macro-averaged metrics, and stage-specific metrics for c) Wake, d) N1, e) N2, f) N3, and g) REM. Significant group differences (Mann–Whitney U test, $p < 0.05$) are indicated with asterisks. Home-based studies showed significantly better performance in N3-stage κ, SE, F1, and MCC, as well as N1-stage ACC. Conversely, REM-stage ACC and NPV were significantly higher in controlled settings. ACC: accuracy; κ: Cohen's kappa; MCC: Matthews correlation coefficient; SE: sensitivity; SP: specificity; PPV: positive predictive value; NPV: negative predictive value.

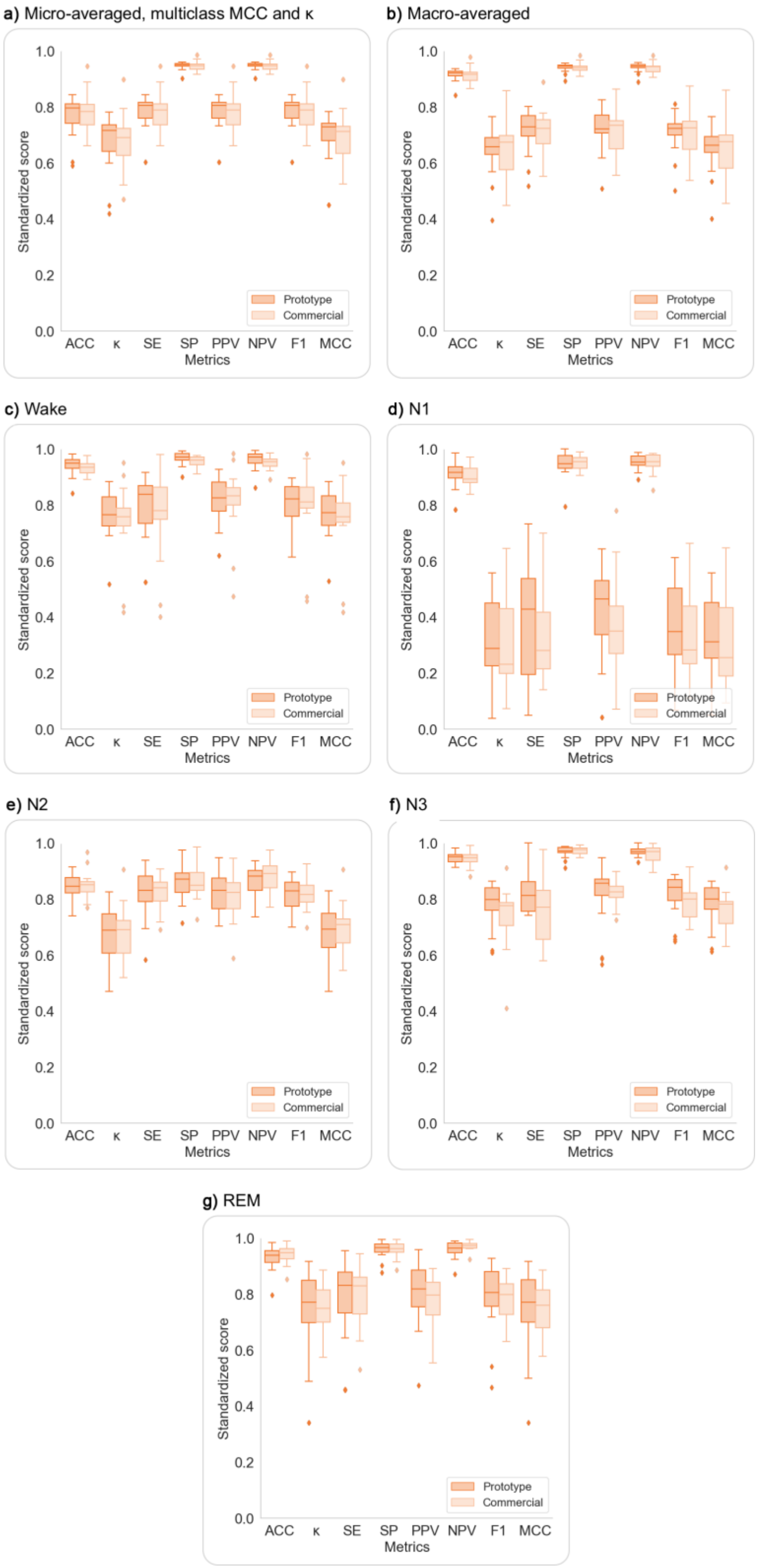

Supplementary figure S5. Influence of device type (prototype vs commercial) on wEEG evaluation metrics. This figure presents standardized scores for prototype-based (light orange) and commercial (dark orange) systems across a) micro-averaged and multiclass κ and MCC, b) macro-averaged metrics, and stage-specific metrics for c) Wake, d) N1, e) N2, f) N3, and g) REM. No statistically significant differences were found between the two groups. However, prototype devices showed slightly higher median sensitivity (SE) for Wake, N1, and N3 stages, while commercial systems performed marginally better on N2 and REM SE. ACC: accuracy; κ: Cohen's kappa; MCC: Matthews correlation coefficient; SE: sensitivity; SP: specificity; PPV: positive predictive value; NPV: negative predictive value.

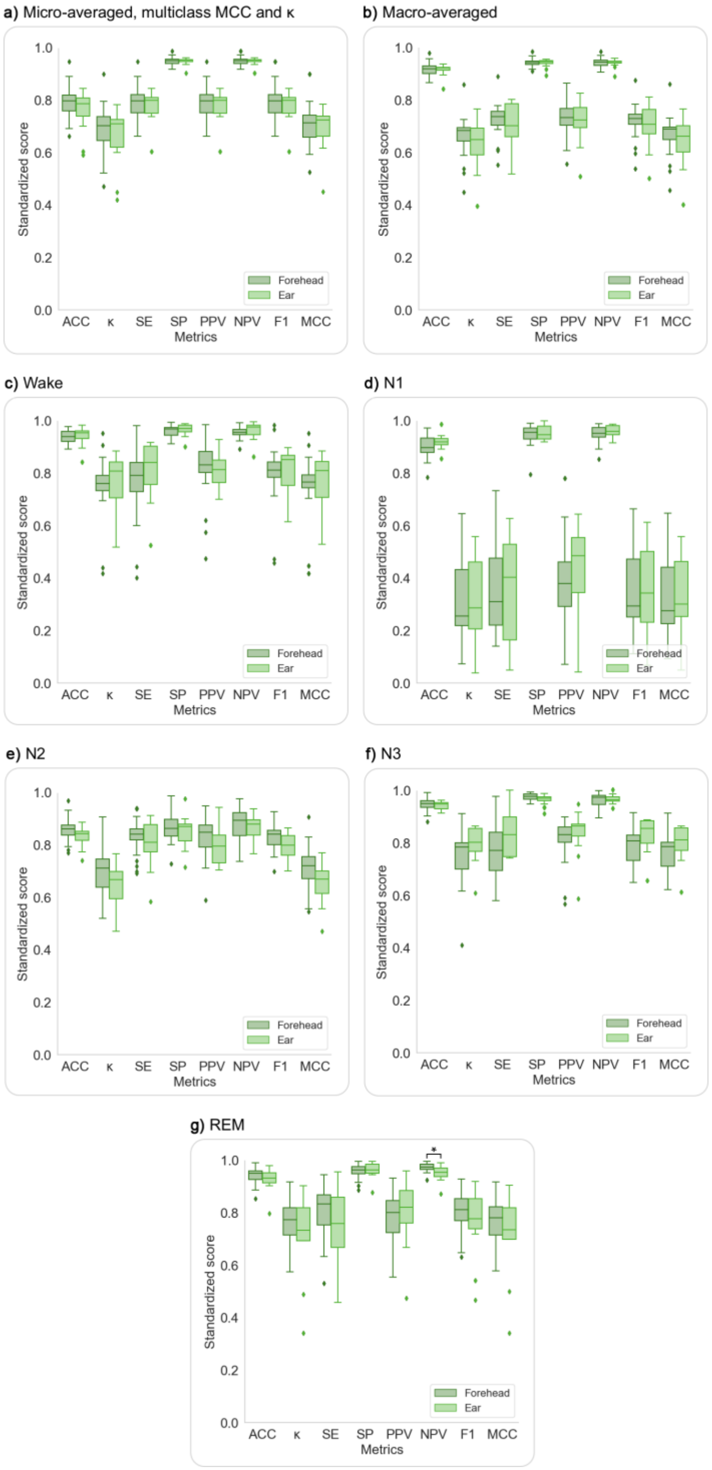

Supplementary figure S6. Influence of electrode placement (forehead vs. ear) on wEEG evaluation metrics. This figure compares standardized performance metrics between studies using forehead-placed electrodes (light green) and ear-placed electrodes (dark green). Panels display results for a) micro-averaged and multiclass κ and MCC, b) macro-averaged metrics, and stage-specific metrics for c) Wake, d) N1, e) N2, f) N3, and g) REM. Significant group differences (Mann–Whitney U test, $p < 0.05$) are indicated with asterisks. A significant difference was observed only for REM NPV, which was higher in studies using forehead electrodes. Overall, performance patterns were largely similar between the two configurations. Ear-placed systems showed slightly better average SE for Wake, N1, N2, and N3, while forehead-based devices performed marginally better on REM SE, though these trends did not reach statistical significance. ACC: accuracy; κ: Cohen's kappa; MCC: Matthews correlation coefficient; SE: sensitivity; SP: specificity; PPV: positive predictive value; NPV: negative predictive value.

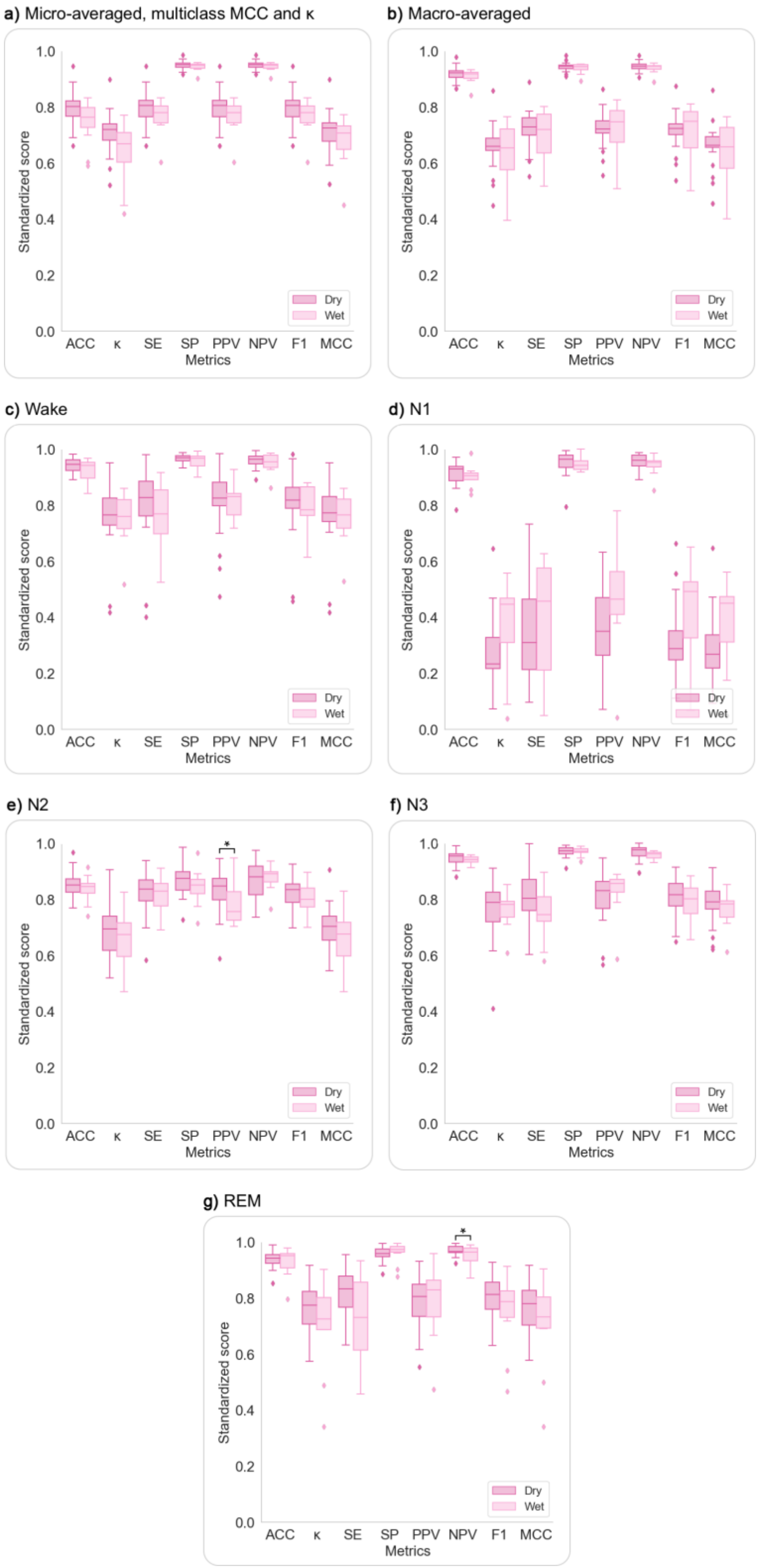

Supplementary figure S7. Influence of electrode type (dry vs. wet) on wEEG evaluation metrics. This figure illustrates standardized scores across studies that used dry (light pink) versus wet (dark pink) electrodes, comparing a) micro-averaged metrics, κ and MCC, b) macro-averaged metrics, and stage-specific metrics for c) Wake, d) N1, e) N2, f) N3, and g) REM. Significant group differences (Mann–Whitney U test, $p < 0.05$) are indicated with asterisks. While most metrics showed similar performance across groups, two statistically significant differences were found: N2 PPV and REM NPV were higher in studies using dry electrodes. Minor trends also suggest that wet electrodes may offer slightly better SE in N3, while dry systems had marginal advantages in REM-stage SP and PPV values. ACC: accuracy; κ: Cohen's kappa; MCC: Matthews correlation coefficient; SE: sensitivity; SP: specificity; PPV: positive predictive value; NPV: negative predictive value.

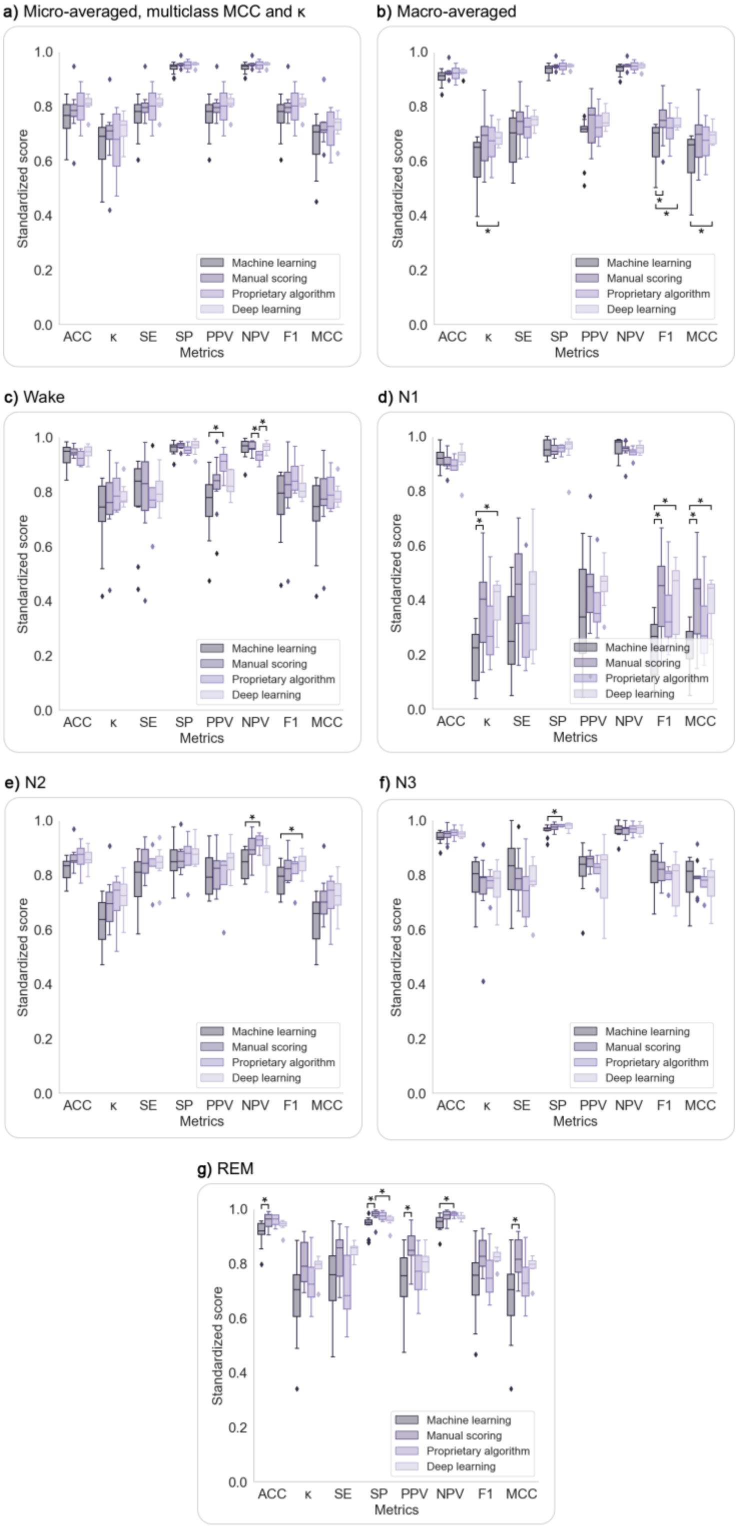

Supplementary figure S8. Influence of scoring method on wEEG evaluation metrics. Boxplots compare standardized scores across four scoring approaches: machine learning (dark grey), manual scoring (grey), proprietary algorithms (light purple), and deep learning (lavender) for a) micro-averaged and multiclass metrics, b) macro-averaged metrics, and stage-specific metrics for c) Wake, d) N1, e) N2, f) N3, and g) REM. Significant group differences (Mann–Whitney U test, $p < 0.05$) are indicated with asterisks. Deep learning outperformed machine learning in macro-averaged κ, F1, and MCC, as well as N1 κ, F1, and MCC. Manual scoring showed higher performance than machine learning in macro F1, N1 metrics, and several REM metrics. Proprietary algorithms showed significantly better results for Wake PPV, N2 NPV, N3 SP, and REM NPV compared to machine learning, and also outperformed other methods for Wake and REM PPV/NPV. ACC: accuracy; κ: Cohen's kappa; MCC: Matthews correlation coefficient; SE: sensitivity; SP: specificity; PPV: positive predictive value; NPV: negative predictive value.

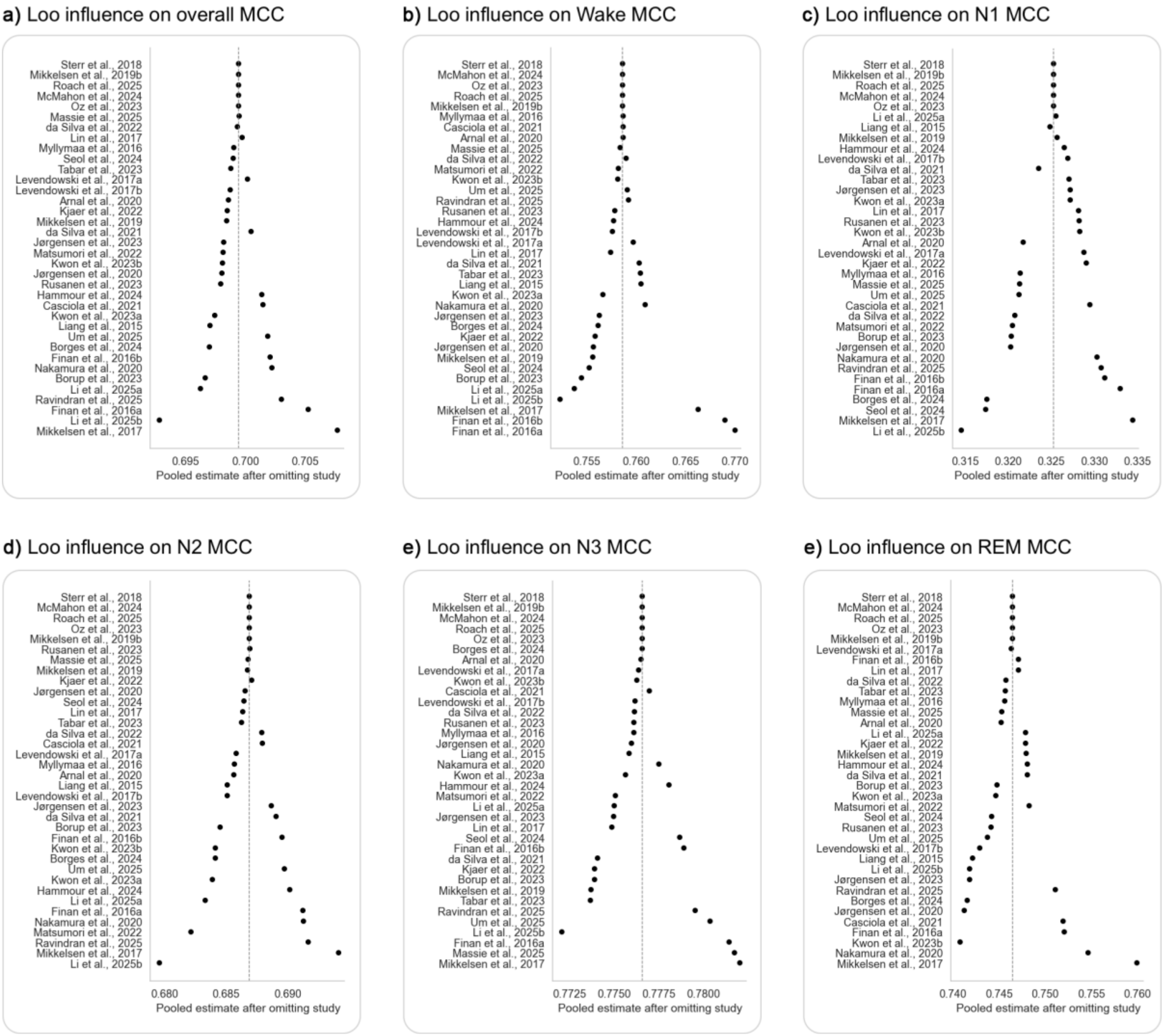

Supplementary figure S9. Leave-one-out (LOO) sensitivity analysis for pooled Matthews correlation coefficient (MCC) estimates from studies classifying five sleep stages. This analysis includes 36 results from 32 studies; four studies reported outcomes for two device scoring methods and were treated as separate results. Each panel shows how the pooled MCC changes when omitting one result at a time (black dots), compared to the baseline estimate using all 36 (dashed vertical line). The threshold for influential change was defined as an absolute shift > 0.03. No result exceeded this cutoff, indicating robustness of the pooled MCCs across all stages. **a) Overall MCC:** Omitting any one result shifted the overall MCC by no more than ±0.008 (1.2%). The largest upward shift occurred when *Mikkelsen et al.*, 2017 was excluded. **b) Wake-stage MCC.** Shifts were ≤ ±0.011 (1.5%); *Finan et al.*, 2016a yielded the greatest increase. **c) N1-stage MCC:** variability reached ±0.011 (3.3%); removal of Li et al., 2025 produced the largest decrease.**d) N2-stage MCC:** deviations were ≤ ±0.007 (1.1%), driven chiefly by *Li et al.*, 2025b. **e) N3-stage MCC:** the estimate varied by ≤ ±0.005 (0.7%), with *Mikkelsen et al.*, 2017 providing the strongest positive influence. **f) REM-stage MCC.** the largest shift (+0.014, 1.8%) appeared when *Mikkelsen et al.*, 2017 was omitted. Across all stages, the maximum absolute change in pooled MCC was below 0.03, the predefined sensitivity threshold. This confirms that the meta-analytic results for studies using five-stage classification are robust and not disproportionately influenced by any single result.

Confusion matrices

A

GT \ WD	W	N1	N2	N3	REM	Sum
**W**	**16413**	246	211	17	29	16916
**N1**	341	**473**	462	19	87	1382
**N2**	239	323	**4832**	363	63	5820
**N3**	17	7	63	**1167**	0	1254
**REM**	51	259	131	0	**761**	1202
**Sum**	17061	1308	5699	1566	940	**26574**

B

GT \ WD	W	N1	N2	N3	REM	Sum
**W**	**16576**	259	61	6	14	16916
**N1**	135	**966**	195	8	77	1381
**N2**	118	201	**5275**	183	39	5816
**N3**	0	2	28	**1213**	0	1243
**REM**	17	99	17	0	**1067**	1200
**Sum**	16846	1527	5576	1410	1197	**26556**

Supplementary figure 9**. Confusion matrix from Li et al., 2025**[1]. The number of epochs in each cell was calculated from the contingency table and the total number of epochs per each stage as staged by the gold standard. **A:** Results from automatic scoring. **B:** Results from manual scoring. GT: ground truth, W: Wake, WD: wearable EEG device.

GT \ WD	W	N1	N2	N3	REM	Sum
**W**	**14821**	1382	2714	194	880	19991
**N1**	956	**984**	3979	51	1005	6975
**N2**	495	430	**17065**	1116	793	19899
**N3**	177	60	3408	**6666**	31	10342
**REM**	139	58	1820	2	**4320**	6339
**Sum**	16588	2914	28986	8029	7029	**63546**

Supplementary figure S10. Confusion matrix from Ravindran et al., 2025[2]. GT: ground truth, W: Wake, WD: wearable EEG device.

GT \ WD	W	N1	N2	N3	REM	Sum
**W**	**10032**	668	155	1	83	10939
**N1**	1623	**11380**	5749	121	1511	20384
**N2**	114	1590	**24821**	849	229	27603
**N3**	4	46	2075	**4230**	0	6355
**REM**	91	906	527	0	**8772**	10296
**Sum**	11864	14590	33327	5201	10595	**75577**

Supplementary figure S11. Confusion matrix from Seol et al., 2025[3]. GT: ground truth, W: Wake, WD: wearable EEG device.

GT \ WD	W	N1	N2	N3	REM	Sum
**W**	**1147**	29	186	0	72	1434
**N1**	41	**45**	99	2	28	215
**N2**	114	76	**3280**	153	191	3814
**N3**	20	0	445	**1555**	0	2020
**REM**	56	0	185	19	**1594**	1854
**Sum**	1378	150	4195	1729	1885	**9337**

Supplementary figure S12. Confusion matrix from Rusanen et al., 2023[4]. The number of epochs in each cell was calculated from the contingency table and the total number of epochs per each stage as staged by the gold standard. GT: ground truth, W: Wake, WD: wearable EEG device.

GT \ WD	W	N1	N2	N3	REM	Sum
**W**	**1787**	156	74	9	163	2189
**N1**	109	**192**	201	2	182	686
**N2**	206	326	**3404**	350	329	4615
**N3**	7	1	132	**961**	6	1107
**REM**	105	120	79	2	**844**	1150
**Sum**	2214	795	3890	1324	1524	**9747**

Supplementary figure S13. Confusion matrix from Casciola et al., 2021[5]. For each participant (*n* = 12), number of epochs in each cell was calculated from the contingency table and total number of epochs per each stage as staged by the gold standard. The total confusion matrix was calculated as the sum of all confusion matrices. GT: ground truth, W: Wake, WD: wearable EEG device.

GT \ WD	W	N1	N2	N3	REM	Sum
W	**2400**	341	215	26	109	3091
N1	278	**764**	344	17	203	1606
N2	175	404	**10100**	822	715	12216
N3	5	2	377	**2660**	27	3071
REM	150	144	363	0	**4021**	4678
Sum	3008	1655	11399	3525	5075	**24662**

Supplementary figure S14. Confusion matrix from Arnal et al., 2020i et a[6]. GT: ground truth, W: Wake, WD: wearable EEG device.

GT \ WD	W	N1	N2	N3	REM	Sum
W	**1276**	103	83	6	50	1518
N1	188	**243**	391	7	255	1084
N2	49	118	**2651**	196	105	3119
N3	4	0	154	**1117**	8	1283
REM	28	100	77	4	**1038**	1247
Sum	1545	564	3356	1330	1456	**8251**

Supplementary figure S15. Confusion matrix from Lin et al., 2017[7]. The number of epochs in each cell was calculated from the contingency table and the total number of epochs per each stage as staged by the gold standard. GT: ground truth, W: Wake, WD: wearable EEG device.

A

GT \ WD	W	N1	N2	N3	REM	Sum
W	**6619**	1162	576	19	245	8621
N1	773	**845**	677	15	367	2677
N2	306	1073	**12243**	595	519	14736
N3	16	6	793	**3062**	0	3877
REM	338	152	115	30	**3089**	3724
Sum	8052	3238	14404	3721	4220	**33635**

B

GT \ WD	W	N1	N2	N3	REM	Sum
W	**6960**	1050	433	11	80	8534
N1	738	**1063**	765	22	370	2958
N2	216	1086	**12506**	603	391	14802
N3	8	3	779	**3086**	0	3876
REM	96	59	43	0	**3361**	3559
Sum	8018	3261	14526	3722	4202	**33729**

Supplementary figure S16. Confusion matrix from Levendowski et al., 2017[8]. The number of epochs in each cell was calculated from the contingency table and the total number of epochs per each stage as staged by the gold standard. **A:** Results from automatic scoring. **B:** Results from automatic scoring corrected by reviewer. GT: ground truth, W: Wake, WD: wearable EEG device.

A

GT \ WD	W	N1	N2	N3	REM	Sum
W	**432**	309	74	16	145	976
N1	54	**94**	124	7	68	347
N2	215	398	**4533**	386	779	6311
N3	92	52	819	**1811**	230	3004
REM	118	474	174	8	**2033**	2807
Sum	911	1327	5724	2228	3255	**13445**

B

GT \ WD	W	N1	N2	N3	REM	Sum
W	**392**	269	86	23	207	977
N1	46	**94**	132	11	65	348
N2	141	249	**4789**	503	628	6310
N3	55	23	691	**2222**	14	3005
REM	49	141	191	11	**2389**	2781
Sum	683	776	5889	2770	3303	**13421**

Supplementary figure S17. Confusion matrix from Finan et al., 2016[9]**.** The number of epochs in each cell was calculated from the dichotomized % agreement table and the total number of epochs per each stage as staged by PSG. The number of wake epochs was calculated as 14 (participants) x 2 (two epochs per minute) x 34.9 (mean WASO minutes + mean SOL minutes recorded by PSG). Similarly, the N1 epochs as 14 x 2 x 12.43, the N2 epochs as 14 x 2 x 225.36, the N3 epochs as 14 x 2 x 107.32 and the REM epochs as 14 x 2 x 100.25. The calculated evaluation metrics matched the reported ones (κ, SE and SP). **A:** Results from automatic scoring. **B:** Results from manual scoring. GT: ground truth, W: Wake, WD: wearable EEG device.

GT \ WD	W	N1	N2	N3	REM	Sum
W	**290**	34	6	0	16	346
N1	30	**57**	25	1	20	133
N2	115	69	**2568**	169	172	3093
N3	24	11	237	**1183**	1	1456
REM	9	24	50	2	**1367**	1452
Sum	468	195	2886	1355	1576	**6480**

Supplementary figure S18. Confusion matrix from Liang et al., 2015[10]. GT: ground truth, W: Wake, WD: wearable EEG device.

GT WD	W	N1	N2	N3	REM	Sum
**W**	**16017**	1240	1331	57	370	19015
**N1**	964	**2010**	1084	18	186	4262
**N2**	2329	785	**34417**	3529	1157	42217
**N3**	93	5	1395	**4836**	51	6380
**REM**	587	207	1154	84	**7880**	9912
**Sum**	19990	4247	39381	8524	9644	**81786**

Supplementary figure S19. Confusion matrix from Massie et al., 2025[11]. GT: ground truth, W: Wake, WD: wearable EEG device.

GT WD	W	N1	N2	N3	REM	Sum
**W**	**42**	10	3	2	1	58
**N1**	4	**93**	21	0	9	127
**N2**	4	92	**246**	7	4	353
**N3**	0	0	4	**13**	0	17
**REM**	2	13	7	0	**109**	131
**Sum**	52	208	281	22	123	**686**

Supplementary figure S20. Confusion matrix from Um et al., 2025l.,[12]. The number of epochs in each cell was calculated from the contingency table and the total number of epochs per each stage as staged by the gold standard. GT: ground truth, W: Wake, WD: wearable EEG device.

A

GT WD	W	N1	N2	N3	REM	Sum
**W**	**947**	39	101	1	44	1132
**N1**	69	**57**	155	0	62	343
**N2**	39	11	**2268**	60	45	2423
**N3**	3	0	104	**388**	0	495
**REM**	16	2	48	0	**502**	568
**Sum**	1074	109	2676	449	653	**4961**

B

GT WD	W	N1	N2	N3	REM	Sum
**W**	**824**	201	108	1	7	1141
**N1**	39	**104**	176	0	24	343
**N2**	18	66	**2272**	59	8	2423
**N3**	0	0	122	**373**	0	495
**REM**	9	5	30	0	**524**	568
**Sum**	890	376	2708	433	563	**4970**

Supplementary figure S21. Confusion matrices from Kwon et al., 2023[13]. **A:** Results from automatic scoring. **B:** Results from manual scoring. GT: ground truth, W: Wake, WD: wearable EEG device.

GT WD	W	N1	N2	N3	REM	Sum
**W**	**1419**	20	0	20	559	2018
**N1**	24	**1436**	195	316	487	2458
**N2**	0	645	**9136**	215	752	10748
**N3**	0	431	86	**3623**	129	4269
**REM**	165	494	219	55	**4553**	5486
**Sum**	1608	3026	9636	4229	6480	**24979**

Supplementary figure S22. Confusion matrix from Matsumori et al., 2022[14]. The number of epochs in each cell was calculated from the contingency table and the total number of epochs per each stage as staged by the gold standard. GT: ground truth, W: Wake, WD: wearable EEG device.

A

GT WD	W	N1	N2	N3	REM	A	Sum
**W**	**3094**	684	212	10	27	33	4060
**N1**	656	**1472**	629	9	149	13	2928
**N2**	159	769	**11342**	469	133	65	12937
**N3**	22	30	1422	**3809**	0	24	5307
**REM**	24	257	674	12	**1492**	1	2460
**A**	0	0	0	0	0	**0**	0
**Sum**	3955	3212	14279	4309	1801	136	**27692**

B

GT WD	W	N1	N2	N3	REM	A	Sum
**W**	**3486**	1022	121	19	17	53	4718
**N1**	330	**1390**	295	1	151	45	2212
**N2**	117	743	**10213**	503	277	118	11971
**N3**	20	36	867	**4464**	27	38	5452
**REM**	3	148	221	10	**2936**	21	3339
**A**	0	0	0	0	**0**	**0**	0
**Sum**	3956	3339	11717	4997	3408	275	**27692**

Supplementary figure S23. Confusion matrices from Myllymaa et al., 2016[15]. **A:** Scorer 1. **B:** Scorer 2. The evaluation metrics were calculated for both scorers and the average of the two scorers was used for analysis. A: artifact, GT: ground truth, W: Wake, WD: wearable EEG device.

GT \ WD	W	N1	N2	N3	REM	Sum
W	**2432**	336	181	2	38	2989
N1	158	**993**	265	1	133	1550
N2	17	234	**4839**	186	33	5309
N3	7	6	433	**1274**	0	1720
REM	10	28	72	0	**1541**	1651
Sum	2624	1597	5790	1463	1745	**13219**

Supplementary figure S24. Confusion matrix from Borges et al., 2025[16]. GT: ground truth, W: Wake, WD: wearable EEG device.

GT \ WD	W	N1	N2	N3	REM	Sum
W	**4325**	312	138	12	35	4822
N1	403	**573**	519	1	95	1591
N2	319	407	**2894**	323	61	4004
N3	38	1	400	**1259**	0	1698
REM	94	205	161	0	**828**	1288
Sum	5179	1498	4112	1595	1019	**13403**

Supplementary figure S25. Confusion matrix from Hammour et al., 2024[17]. GT: ground truth, W: Wake, WD: wearable EEG device.

GT \ WD	W	N1	N2	N3	REM	Sum
W	**3200**	171	66	0	57	3494
N1	225	**888**	523	8	356	2000
N2	100	312	**10671**	284	606	11973
N3	5	18	1058	**5193**	0	6274
REM	108	163	525	0	**4580**	5376
Sum	3638	1552	12843	5485	5599	**29117**

Supplementary figure S26. Confusion matrix from Borup et al., 2023 20[18]. GT: ground truth, W: Wake, WD: wearable EEG device.

GT \ WD	W	N1	N2	N3	REM	Sum
W	**953**	72	156	2	71	1254
N1	233	**213**	666	12	165	1289
N2	99	58	**6687**	341	297	7482
N3	7	1	245	**2385**	0	2638
REM	72	44	474	0	**2456**	3046
Sum	1364	388	8228	2740	2989	**15709**

Supplementary figure S27. Confusion matrix from Tabar et al., 2023[19]. GT: ground truth, W: Wake, WD: wearable EEG device.

GT \ WD	W	N1	N2	N3	REM	Sum
W	**72**	7	0	0	1	80
N1	15	**28**	9	1	5	58
N2	4	101	**374**	111	51	641
N3	0	0	0	**333**	0	333
REM	1	6	14	0	**445**	466
Sum	92	142	397	445	502	**1578**

Supplementary figure S28. Confusion matrix from Jørgensen et al., 2023[20]. GT: ground truth, W: Wake, WD: wearable EEG device.

A

GT \ WD	W	N1	N2	N3	REM	Sum
W	**9697**	111	551	17	220	10596
N1	1170	**433**	2536	35	1054	5228
N2	739	104	**27076**	1604	1882	31405
N3	16	0	1216	**11415**	1	12648
REM	150	96	2954	44	**9821**	13065
Sum	11772	744	34333	13115	12978	**72942**

B

GT \ WD	W	N1	N2	N3	REM	Sum
W	**9715**	122	684	41	157	10719
N1	878	**369**	1339	3	717	3306
N2	954	149	**26185**	379	2085	29752
N3	29	1	3041	**12641**	0	15712
REM	196	103	3084	51	**10019**	13453
Sum	11772	744	34333	13115	12978	**72942**

Supplementary figure S29. Confusion matrix from Kjaer et al., 2022[21]. **A:** Scorer 1. **B:** Scorer 2. The evaluation metrics were calculated for both scorers and the average of the two scorers was used for analysis. GT: ground truth, W: Wake, WD: wearable EEG device.

GT \ WD	W	N1	N2	N3	REM	Sum
W	**4010**	294	86	3	20	4413
N1	505	**1690**	456	10	38	2699
N2	219	1543	**9905**	611	75	12353
N3	23	16	896	**3757**	0	4692
REM	29	162	243	3	**2999**	3436
Sum	4786	3705	11586	4384	3132	**27593**

Supplementary figure S30. Confusion matrix from Jørgensen et al. 2020[22]. GT: ground truth, W: Wake, WD: wearable EEG device.

GT \ WD	W	N1	N2	N3	REM	Sum
W	**1908**	3	555	45	57	2568
N1	46	**9**	105	6	17	183
N2	379	1	**4483**	219	250	5332
N3	47	0	455	**1530**	16	2048
REM	93	1	679	29	**677**	1479
Sum	2473	14	6277	1829	1017	**11610**

Supplementary figure S31. Confusion matrix from Nakamura et al., 2020[23]. GT: ground truth, W: Wake, WD: wearable EEG device.

GT \ WD	W	N1	N2	N3	REM	Sum
W	**9458**	985	706	11	126	11286
N1	332	**1188**	446	7	313	2286
N2	449	2147	**27537**	1894	2901	34928
N3	11	19	828	**10734**	15	11607
REM	172	886	1895	2	**9716**	12671
Sum	10422	5225	31412	12648	13071	**72778**

Supplementary figure S32. Confusion matrix from Mikkelsen et al., 2019[24]. GT: ground truth, W: Wake, WD: wearable EEG device.

GT \ WD	W	N1	N2	N3	REM	Sum
W	**939**	122	340	92	296	1789
N1	11	**22**	73	8	20	134
N2	145	234	**2244**	198	412	3233
N3	3	10	190	**608**	6	817
REM	173	137	336	130	**662**	1438
Sum	1271	525	3183	1036	1396	**7411**

Supplementary figure S33. Confusion matrix from Mikkelsen et al., 2017[25]. GT: ground truth, W: Wake, WD: wearable EEG device.

GT \ WD	W	N1	N2	N3	REM	A	Sum
W	**934**	110	40	10	15	2	1111
N1	175	**476**	159	14	32	1	857
N2	90	204	**2832**	345	92	8	3571
N3	15	14	495	**2620**	4	0	3148
REM	88	222	219	24	**1387**	0	1940
A	0	0	0	0	5	**0**	5
Sum	1302	1026	3745	3013	1535	11	**10632**

Supplementary figure S34. Confusion matrix from da Silva Suoto et al., 2022[26]. GT: ground truth, W: Wake, WD: wearable EEG device

GT \ WD	W	N1	N2	N3	REM	A	Sum
**W**	**769**	47	135	10	98	63	1122
**N1**	86	**257**	306	3	60	27	739
**N2**	71	133	**2958**	244	82	135	3623
**N3**	2	1	198	**1753**	1	2	1957
**REM**	23	70	399	2	**1191**	83	1768
**A**	8	2	19	1	2	**100**	132
**Sum**	959	510	4015	2013	1434	410	**9341**

Supplementary figure S35. Confusion matrix from da Silva Suoto et al., 202125a[127]. GT: ground truth, W: Wake, WD: wearable EEG device.

GT \ WD	W	Light	Deep	REM	Sum
**W**	**21138**	4452	102	815	26507
**Light**	6805	**98618**	4991	4537	114951
**Deep**	237	4737	**19402**	47	24423
**REM**	950	4010	5	**23740**	28705
**Sum**	29130	111817	24500	29139	**194586**

Supplementary figure S36. Confusion matrix from Chen et al., 2023[28]. GT: ground truth, W: Wake, WD: wearable EEG device.

A

GT \ WD	W	Light	Deep	REM	Error	Sum
**W**	**414**	426	79	224	74	1217
**Light**	187	**6622**	806	940	155	8710
**Deep**	2	815	**1931**	1	6	2755
**REM**	45	728	54	**2387**	79	3293
**Error**	0	0	0	0	**0**	0
**Sum**	648	8591	2870	3552	314	**15661**

B

GT \ WD	W	Light	Deep	REM	Error	Sum
**W**	**1137**	544	245	609	89	2624
**Light**	186	**4168**	1037	965	216	6572
**Deep**	2	683	**1237**	0	30	1952
**REM**	26	232	242	**1791**	21	2312
**Error**	0	0	0	0	**0**	0
**Sum**	1351	5627	2761	3365	356	**13104**

C

GT \ WD	W	Light	Deep	REM	Error	Sum
**W**	**1551**	970	324	833	163	3841
**Light**	373	**10790**	1843	1905	371	15282
**Deep**	4	1498	**3168**	1	36	4707
**REM**	71	960	296	**4178**	100	5605
**Error**	0	0	0	0	**0**	0
**Sum**	1999	14218	5631	6917	670	**28765**

Supplementary figure S37. Confusion matrices from Markwald et al., 2016[29]**.A :** 97% sleep efficiency group. B: 72% sleep efficiency group. **C:** Combined groups. GT: ground truth, W: Wake, WD: wearable EEG device.

GT \ WD	W	Light	Deep	REM	Sum
**W**	**109**	12	3	48	172
**Light**	91	**1666**	33	368	2158
**Deep**	6	144	**1006**	0	1156
**REM**	13	28	0	**708**	749
**Sum**	219	1850	1042	1124	**4235**

Supplementary figure S38. Confusion matrix from Rostaminia et al., 2022[30]. GT: ground truth, W: Wake, WD: wearable EEG device.

GT \ WD	W	Light	Deep	REM	Sum
**W**	**616**	99	2	6	723
**Light**	97	**3389**	164	238	3888
**Deep**	5	236	**1171**	1	1413
**REM**	8	148	0	**1302**	1458
**Sum**	726	3872	1337	1547	**7482**

Supplementary figure S39. Confusion matrix from Hsieh et al., 2021[31]. GT: ground truth, W: Wake, WD: wearable EEG device.

GT \ WD	W	Light	Deep	REM	Sum
**W**	**15353**	1109	0	446	16908
**Light**	2721	**38769**	1385	3548	46423
**Deep**	59	1700	**4974**	3	6736
**REM**	81	4136	1	**10921**	15139
**Sum**	18214	45714	6360	14918	**85206**

Supplementary figure S40. Confusion matrix from Kaplan et al., 2015[32]. GT: ground truth, W: Wake, WD: wearable EEG device.

GT \ WD	W	N1	N2	N3	Sum
**W**	**53**	5	8	1	67
**N1**	12	**23**	11	0	46
**N2**	3	5	**125**	7	140
**N3**	0	2	14	**24**	40
**Sum**	68	35	158	32	**293**

Supplementary figure S41. Confusion matrix from Nakamura et al., 2017[33]. GT: ground truth, W: Wake, WD: wearable EEG device.

Supplementary tables

Supplementary Table S1. Overall and sleep-stage specific evaluation metrics for studies classifying 5 stages. The table supports Figure 5. Significant *p*-values are highlighted in bold.

			Stats					Evaluation metric vs MCC	
		Metric	Mean	SD	Min	Max	Count	U-stat	*p*-value
Overall	Micro-averaged	κ	0.68	0.10	0.42	0.90	36	639.50	0.30836
		MCC	0.70	0.08	0.45	0.90	31		
		ACC	0.78	0.07	0.59	0.95	34	215.50	**0.00004**
		SE	0.79	0.06	0.60	0.95	31	4.00	0.09524
		SP	0.95	0.02	0.90	0.99	31	0.00	**0.01193**
		PPV	0.79	0.06	0.60	0.95	31	4.00	0.09524
		NPV	0.95	0.02	0.90	0.99	31	0.00	**0.01193**
		F1	0.79	0.06	0.60	0.95	31	4.00	0.09524
	Macro-averaged	ACC	0.92	0.03	0.84	0.98	31	0.00	**0.00433**
		κ	0.65	0.09	0.40	0.86	31	18.00	0.64658
		SE	0.71	0.08	0.52	0.89	31	4.00	0.09524
		SP	0.94	0.02	0.89	0.98	31	0.00	**0.00794**
		PPV	0.72	0.08	0.51	0.86	31	11.00	0.53680
		NPV	0.94	0.02	0.89	0.98	31	0.00	**0.00794**
		F1	0.71	0.08	0.50	0.88	30	6.00	0.22222
		MCC	0.65	0.09	0.40	0.86	31		
Wake		ACC	0.94	0.03	0.84	0.98	32	31.00	**0.00000**
		κ	0.75	0.12	0.42	0.95	32	522.50	0.72066
		SE	0.79	0.13	0.40	0.98	34	434.00	0.22436
		SP	0.96	0.02	0.90	0.99	33	10.00	**0.00000**
		PPV	0.81	0.11	0.47	0.98	33	342.00	**0.02318**
		NPV	0.96	0.03	0.86	1.00	32	14.00	**0.00000**
		F1	0.80	0.11	0.46	0.98	33	367.00	0.05303
		MCC	0.76	0.12	0.42	0.95	31		
N1		ACC	0.91	0.04	0.78	0.99	32	0.00	**0.00000**
		κ	0.31	0.15	0.04	0.65	32	542.50	0.52707
		SE	0.36	0.18	0.05	0.73	34	464.50	0.41544
		SP	0.95	0.04	0.79	1.00	33	0.00	**0.00000**
		PPV	0.40	0.17	0.04	0.78	33	365.00	**0.04983**
		NPV	0.95	0.03	0.85	0.99	32	0.00	**0.00000**
		F1	0.35	0.16	0.07	0.66	33	452.00	0.42799
		MCC	0.33	0.15	0.05	0.65	31		
N2		ACC	0.85	0.05	0.74	0.97	32	53.00	**0.00000**
		κ	0.68	0.09	0.47	0.91	32	530.00	0.64506
		SE	0.82	0.08	0.58	0.94	34	143.50	**0.00000**
		SP	0.86	0.06	0.71	0.99	33	56.00	**0.00000**
		PPV	0.81	0.08	0.59	0.95	33	138.50	**0.00000**
		NPV	0.87	0.06	0.74	0.97	32	48.00	**0.00000**
		F1	0.81	0.06	0.70	0.93	33	110.50	**0.00000**
		MCC	0.69	0.09	0.47	0.91	31		
N3		ACC	0.94	0.02	0.88	0.99	32	2.00	**0.00000**
		κ	0.76	0.10	0.41	0.91	32	533.50	0.61093
		SE	0.79	0.10	0.58	1.00	34	490.00	0.63164
		SP	0.97	0.02	0.91	0.99	33	1.00	**0.00000**
		PPV	0.82	0.09	0.57	0.95	33	306.00	**0.00588**
		NPV	0.96	0.02	0.89	1.00	32	1.00	**0.00000**
		F1	0.80	0.07	0.65	0.91	33	397.00	0.12560
		MCC	0.78	0.07	0.61	0.91	31		
REM		ACC	0.93	0.04	0.80	0.99	32	30.00	**0.00000**

κ	0.74	0.13	0.34	0.92	32	518.00	0.76750
SE	0.79	0.13	0.46	0.96	34	421.00	0.16583
SP	0.96	0.03	0.88	1.00	33	12.00	**0.00000**
PPV	0.79	0.11	0.47	0.96	33	407.00	0.16236
NPV	0.96	0.03	0.87	1.00	32	6.00	**0.00000**
F1	0.79	0.10	0.47	0.93	33	405.50	0.15638
MCC	0.75	0.13	0.34	0.92	31		

ACC: accuracy, κ: Cohen's Kappa, MCC: Matthew's Correlation Coefficient, NPV: negative predictive value, PPV: positive predictive value, SE: sensitivity, SP: specificity.

Supplementary Table S2. Significant differences between micro- and macro-averaged evaluation metrics in studies classifying 5 stages. The table supports Figure 5b. Significant *p*-values are highlighted in bold.

Metric	p-value
Micro- vs macro-averaged ACC	**0.00000**
Overall vs macro-averaged κ	0.07016
Micro- vs macro-averaged SE	**0.00030**
Micro- vs macro-averaged SP	0.15624
Micro- vs macro-averaged PPV	**0.00053**
Micro- vs macro-averaged NPV	0.19784
Micro- vs macro-averaged F1	**0.00012**
Multiclass MCC vs macro-averaged MCC	**0.04680**

ACC: accuracy, κ: Cohen's Kappa, MCC: Matthew's Correlation Coefficient, NPV: negative predictive value, PPV: positive predictive value, SE: sensitivity, SP: specificity.

Supplementary Table S3. Overall and sleep-stage specific evaluation metrics for studies classifying 4 stages. The table supports Figure 6. Significant *p*-values are highlighted in bold.

		Stats						Evaluation metric vs MCC	
		**Metric**	**Mean**	**SD**	**Min**	**Max**	**Count**	**U-stat**	***p*-value**
**Overall**	**Micro-averaged**	κ	0.70	0.11	0.50	0.79	5	639.50	0.30836
		MCC	0.70	0.11	0.51	0.79	5		
		ACC	0.80	0.08	0.67	0.87	5	215.50	**0.00004**
		SE	0.80	0.08	0.67	0.87	5	4.00	0.09524
		SP	0.94	0.01	0.92	0.96	5	0.00	**0.01193**
		PPV	0.80	0.08	0.67	0.87	5	4.00	0.09524
		NPV	0.94	0.01	0.92	0.96	5	0.00	**0.01193**
		F1	0.80	0.08	0.67	0.87	5	4.00	0.09524
	**Macro-averaged**	ACC	0.90	0.04	0.83	0.93	6	0.00	**0.00433**
		κ	0.65	0.15	0.41	0.81	6	18.00	0.64658
		SE	0.80	0.05	0.73	0.86	5	4.00	0.09524
		SP	0.93	0.02	0.90	0.95	5	0.00	**0.00794**
		PPV	0.73	0.12	0.54	0.86	6	11.00	0.53680
		NPV	0.93	0.02	0.90	0.95	5	0.00	**0.00794**
		F1	0.77	0.09	0.63	0.86	5	6.00	0.22222
		MCC	0.68	0.15	0.42	0.81	5		
**Wake**		ACC	0.91	0.07	0.78	0.97	7	31.00	**0.00000**
		κ	0.68	0.14	0.49	0.84	7	522.50	0.72066
		SE	0.68	0.18	0.40	0.91	7	434.00	0.22436
		SP	0.96	0.02	0.92	0.98	6	10.00	**0.00000**
		PPV	0.76	0.14	0.50	0.87	6	342.00	**0.02318**
		NPV	0.97	0.03	0.92	0.98	5	14.00	**0.00000**
		F1	0.71	0.14	0.53	0.87	6	367.00	0.05303
		MCC	0.69	0.16	0.52	0.84	5		
**Light Sleep**		ACC	0.80	0.07	0.70	0.87	7	2.00	**0.01010**
		κ	0.61	0.11	0.46	0.74	7	22.50	0.46255
		SE	0.77	0.09	0.67	0.87	7	7.00	0.10377
		SP	0.78	0.15	0.50	0.91	6	4.00	0.05195
		PPV	0.84	0.06	0.76	0.90	6	0.00	**0.00433**
		NPV	0.79	0.06	0.71	0.86	5	1.00	**0.01587**
		F1	0.81	0.07	0.71	0.87	6	2.00	**0.01732**
		MCC	0.65	0.11	0.46	0.74	5		
**Deep Sleep**		ACC	0.93	0.04	0.86	0.96	7	1.00	**0.00505**
		κ	0.77	0.13	0.53	0.93	7	18.00	1.00000
		SE	0.80	0.07	0.67	0.87	7	14.00	0.62551
		SP	0.96	0.03	0.90	0.99	6	0.00	**0.00433**
		PPV	0.78	0.14	0.56	0.97	6	13.00	0.79221
		NPV	0.96	0.02	0.94	0.98	5	0.00	**0.00794**
		F1	0.78	0.10	0.61	0.92	6	12.00	0.66234
		MCC	0.75	0.13	0.53	0.89	5		
**REM**		ACC	0.88	0.06	0.80	0.95	7	30.00	**0.00000**
		κ	0.65	0.18	0.28	0.83	7	518.00	0.76750
		SE	0.78	0.14	0.52	0.95	7	421.00	0.16583
		SP	0.91	0.05	0.83	0.97	6	12.00	**0.00000**
		PPV	0.64	0.23	0.22	0.84	6	407.00	0.16236
		NPV	0.96	0.02	0.94	0.99	5	6.00	**0.00000**
		F1	0.70	0.18	0.35	0.87	6	405.50	0.15638
		MCC	0.72	0.10	0.58	0.83	5		

ACC: accuracy, κ: Cohen's Kappa, MCC: Matthew's Correlation Coefficient, NPV: negative predictive value, PPV: positive predictive value, SE: sensitivity, SP: specificity.

Supplementary Table S4. Significant differences between micro- and macro-averaged evaluation metrics in studies classifying 4 stages. The table supports Figure 6b. Significant *p*-values are highlighted in bold.

Metric	p-value
Micro- vs macro-averaged ACC	**0.01732**
Overall vs macro-averaged κ	0.61041
Micro- vs macro-averaged SE	0.42064
Micro- vs macro-averaged SP	0.26320
Micro- vs macro-averaged PPV	0.17749
Micro- vs macro-averaged NPV	0.16667
Micro- vs macro-averaged F1	0.30952
Multiclass MCC vs macro-averaged MCC	0.84387

ACC: accuracy, κ: Cohen's Kappa, MCC: Matthew's Correlation Coefficient, NPV: negative predictive value, PPV: positive predictive value, SE: sensitivity, SP: specificity.

Supplementary Table S5. Influence of participant health status on evaluation metrics in studies classifying 5 stages. Significant *p*-values are highlighted in bold.

				Stats					Healthy vs Clinical		Healthy vs Mixed		Clinical vs Mixed	
		**Metric**	**Type**	**Mean**	**SD**	**Min**	**Max**	**Count**	**U-stat**	***p*-value**	**U-stat**	***p*-value**	**U-stat**	***p*-value**
**Overall**		**Multiclass MCC**	Healthy	0.69	0.07	0.45	0.78	21						
			Clinical	0.73	0.03	0.70	0.77	4						
			Mixed	0.75	0.10	0.59	0.90	6	28.00	0.33091	53.00	0.58884	13.00	0.91429
		**κ**	Healthy	0.67	0.09	0.42	0.78	24						
			Clinical	0.72	0.04	0.67	0.77	5						
			Mixed	0.74	0.09	0.58	0.90	7	41.00	0.28536	65.00	0.38200	17.00	1.00000
	**Micro-averaged**	**ACC**	Healthy	0.77	0.07	0.59	0.84	23						
			Clinical	0.81	0.02	0.78	0.83	4						
			Mixed	0.82	0.08	0.69	0.95	7	29.00	0.25973	64.00	0.43247	17.00	0.64848
		**SE**	Healthy	0.78	0.06	0.60	0.84	21						
			Clinical	0.81	0.02	0.78	0.83	4						
			Mixed	0.82	0.08	0.69	0.95	6	29.00	0.35369	51.00	0.50209	13.00	0.91429
		**SP**	Healthy	0.95	0.02	0.90	0.96	21						
			Clinical	0.95	0.01	0.95	0.96	4						
			Mixed	0.95	0.02	0.92	0.99	6	34.00	0.57752	56.00	0.70411	13.00	0.91429
		**PPV**	Healthy	0.78	0.06	0.60	0.84	21						
			Clinical	0.81	0.02	0.78	0.83	4						
			Mixed	0.82	0.08	0.69	0.95	6	29.00	0.35369	51.00	0.50209	13.00	0.91429
		**NPV**	Healthy	0.95	0.02	0.90	0.96	21						
			Clinical	0.95	0.01	0.95	0.96	4						
			Mixed	0.95	0.02	0.92	0.99	6	34.00	0.57752	56.00	0.70411	13.00	0.91429
		**F1**	Healthy	0.78	0.06	0.60	0.84	21						
			Clinical	0.81	0.02	0.78	0.83	4						
			Mixed	0.82	0.08	0.69	0.95	6	29.00	0.35369	51.00	0.50209	13.00	0.91429
	**Macro-averaged**	**ACC**	Healthy	0.91	0.02	0.84	0.94	21						
			Clinical	0.92	0.01	0.91	0.93	4						
			Mixed	0.93	0.03	0.88	0.98	6	33.00	0.52786	54.00	0.61955	13.00	0.91429
		**κ**	Healthy	0.64	0.08	0.40	0.75	21						
			Clinical	0.72	0.04	0.66	0.77	4						
			Mixed	0.70	0.10	0.54	0.86	6	11.00	**0.01913**	40.00	0.19491	17.00	0.35238
		**SE**	Healthy	0.70	0.07	0.52	0.79	21						
			Clinical	0.78	0.03	0.74	0.80	4	9.00	**0.01597**	47.00	0.36593	17.00	0.35238

		Mixed	0.75	0.08	0.61	0.89	6						
	**SP**	Healthy	0.94	0.02	0.89	0.96	21						
		Clinical	0.95	0.01	0.94	0.95	4						
		Mixed	0.95	0.02	0.92	0.98	6	33.00	0.52834	52.50	0.55939	12.50	1.00000
	**PPV**	Healthy	0.71	0.07	0.51	0.81	21						
		Clinical	0.78	0.04	0.71	0.83	4						
		Mixed	0.75	0.07	0.65	0.86	6	16.00	0.05868	47.50	0.38153	17.00	0.35238
	**NPV**	Healthy	0.94	0.02	0.89	0.96	21						
		Clinical	0.95	0.01	0.94	0.96	4						
		Mixed	0.95	0.02	0.92	0.98	6	37.50	0.76659	54.50	0.64065	12.50	1.00000
	**F1**	Healthy	0.70	0.07	0.50	0.80	20						
		Clinical	0.78	0.03	0.72	0.81	4						
		Mixed	0.75	0.08	0.62	0.88	6	11.00	**0.02277**	37.50	0.18054	17.00	0.35238
	**MCC**	Healthy	0.64	0.08	0.40	0.75	21						
		Clinical	0.73	0.04	0.66	0.77	4						
		Mixed	0.70	0.10	0.55	0.86	6	12.00	**0.02451**	40.00	0.19491	17.00	0.35238
**Wake**	**ACC**	Healthy	0.94	0.03	0.84	0.98	21						
		Clinical	0.94	0.03	0.89	0.96	5						
		Mixed	0.93	0.03	0.89	0.98	6	59.00	0.69612	77.00	0.43101	15.50	1.00000
	**κ**	Healthy	0.75	0.11	0.42	0.88	21						
		Clinical	0.83	0.04	0.77	0.86	4						
		Mixed	0.80	0.09	0.70	0.95	7	15.00	**0.04945**	62.00	0.55943	19.00	0.41212
	**SE**	Healthy	0.78	0.12	0.40	0.92	22						
		Clinical	0.86	0.05	0.78	0.92	5						
		Mixed	0.86	0.10	0.74	0.98	7	27.00	0.08597	50.50	0.18498	19.00	0.87626
	**SP**	Healthy	0.97	0.02	0.90	0.99	21						
		Clinical	0.95	0.03	0.91	0.98	5						
		Mixed	0.95	0.01	0.93	0.97	7	68.00	0.32812	111.00	**0.04926**	19.50	0.80721
	**PPV**	Healthy	0.80	0.10	0.47	0.93	21						
		Clinical	0.84	0.06	0.76	0.93	5						
		Mixed	0.89	0.06	0.82	0.98	7	37.00	0.32903	28.00	**0.01695**	10.00	0.26768
	**NPV**	Healthy	0.96	0.03	0.86	1.00	21						
		Clinical	0.96	0.02	0.93	0.99	5						
		Mixed	0.94	0.02	0.89	0.97	6	57.00	0.79464	98.00	**0.04411**	22.00	0.24675
	**F1**	Healthy	0.78	0.10	0.46	0.90	21						
		Clinical	0.84	0.04	0.77	0.88	5						
		Mixed	0.87	0.08	0.79	0.98	7	24.00	0.06744	37.00	0.05475	17.00	1.00000
	**MCC**	Healthy	0.75	0.10	0.42	0.88	21						
		Clinical	0.83	0.04	0.77	0.86	4	16.00	0.05868	45.00	0.30714	15.00	0.60952

		Mixed	0.82	0.09	0.73	0.95	6						
**N1**	**ACC**	Healthy	0.92	0.04	0.78	0.99	21						
		Clinical	0.90	0.03	0.84	0.95	5						
		Mixed	0.90	0.03	0.87	0.96	6	66.00	0.39759	78.00	0.39767	16.00	0.93074
	**κ**	Healthy	0.33	0.12	0.04	0.47	21						
		Clinical	0.51	0.05	0.44	0.56	4						
		Mixed	0.40	0.18	0.14	0.65	7	3.50	**0.00483**	66.00	0.71031	24.00	0.07273
	**SE**	Healthy	0.42	0.17	0.05	0.73	22						
		Clinical	0.54	0.11	0.22	0.63	5						
		Mixed	0.47	0.19	0.14	0.70	7	25.00	0.06429	72.00	0.82272	24.00	0.34343
	**SP**	Healthy	0.95	0.04	0.79	1.00	21						
		Clinical	0.95	0.02	0.92	0.97	5						
		Mixed	0.96	0.03	0.92	0.99	7	58.00	0.74483	75.00	0.95767	16.00	0.87626
	**PPV**	Healthy	0.45	0.13	0.04	0.64	21						
		Clinical	0.58	0.15	0.38	0.78	5						
		Mixed	0.43	0.12	0.26	0.63	7	29.00	0.13813	72.00	0.95868	29.00	0.07323
	**NPV**	Healthy	0.96	0.03	0.89	0.99	21						
		Clinical	0.93	0.04	0.85	0.97	5						
		Mixed	0.95	0.03	0.90	0.98	6	73.50	0.18192	77.00	0.43080	13.00	0.79221
	**F1**	Healthy	0.37	0.13	0.07	0.56	21						
		Clinical	0.54	0.11	0.28	0.65	5						
		Mixed	0.43	0.16	0.20	0.66	7	16.50	**0.02089**	62.00	0.56778	25.00	0.26768
	**MCC**	Healthy	0.34	0.11	0.05	0.47	21						
		Clinical	0.52	0.05	0.44	0.56	4						
		Mixed	0.42	0.17	0.16	0.65	6	4.00	**0.00543**	53.00	0.57949	20.00	0.11429
**N2**	**ACC**	Healthy	0.84	0.04	0.74	0.92	21						
		Clinical	0.86	0.02	0.84	0.89	5						
		Mixed	0.88	0.06	0.77	0.97	6	40.00	0.43434	35.50	0.11494	8.00	0.24675
	**κ**	Healthy	0.67	0.08	0.47	0.83	21						
		Clinical	0.71	0.03	0.69	0.77	4						
		Mixed	0.73	0.12	0.52	0.91	7	27.00	0.29518	55.00	0.34777	11.00	0.64848
	**SE**	Healthy	0.81	0.09	0.58	0.94	22						
		Clinical	0.86	0.04	0.80	0.91	5						
		Mixed	0.86	0.03	0.83	0.91	7	37.00	0.28445	47.50	0.13937	15.50	0.80721
	**SP**	Healthy	0.86	0.06	0.71	0.98	21						
		Clinical	0.86	0.03	0.82	0.89	5						
		Mixed	0.88	0.08	0.73	0.99	7	55.00	0.89979	66.00	0.71660	14.00	0.63889
	**PPV**	Healthy	0.83	0.07	0.70	0.95	21						
		Clinical	0.81	0.05	0.75	0.87	5	57.00	0.79461	71.50	0.93654	17.00	1.00000

		Mixed	0.82	0.10	0.59	0.95	7						
		Healthy	0.86	0.06	0.74	0.93	21						
	**NPV**	Clinical	0.89	0.05	0.82	0.94	5						
		Mixed	0.92	0.04	0.87	0.97	6	31.00	0.17759	33.00	0.08550	11.00	0.53680
		Healthy	0.81	0.06	0.70	0.90	21						
	**F1**	Clinical	0.83	0.02	0.80	0.86	5						
		Mixed	0.83	0.07	0.70	0.93	7	40.50	0.45427	64.50	0.65196	16.00	0.87626
		Healthy	0.68	0.08	0.47	0.83	21						
	**MCC**	Clinical	0.72	0.03	0.69	0.77	4						
		Mixed	0.75	0.10	0.54	0.91	6	27.50	0.29929	37.50	0.14477	7.00	0.35238
		Healthy	0.94	0.02	0.88	0.98	21						
	**ACC**	Clinical	0.95	0.01	0.94	0.96	5						
		Mixed	0.96	0.02	0.92	0.99	6	51.00	0.94811	40.00	0.18931	11.00	0.53680
		Healthy	0.78	0.07	0.61	0.86	21						
	**κ**	Clinical	0.73	0.07	0.62	0.79	4						
		Mixed	0.77	0.12	0.41	0.91	7	61.00	0.17708	81.00	0.71660	10.00	0.52727
		Healthy	0.82	0.09	0.60	1.00	22						
	**SE**	Clinical	0.72	0.08	0.58	0.80	5						
		Mixed	0.82	0.11	0.65	0.98	7	87.50	**0.04575**	79.00	0.94048	10.00	0.26768
		Healthy	0.97	0.02	0.91	0.99	21						
	**SP**	Clinical	0.98	0.01	0.95	0.99	5						
**N3**		Mixed	0.98	0.01	0.97	0.99	7	31.00	0.17090	34.00	**0.03824**	18.50	0.93517
		Healthy	0.83	0.08	0.59	0.95	21						
	**PPV**	Clinical	0.81	0.10	0.57	0.87	5						
		Mixed	0.83	0.04	0.75	0.89	7	58.50	0.72029	82.00	0.67116	17.00	1.00000
		Healthy	0.96	0.02	0.89	1.00	21						
	**NPV**	Clinical	0.96	0.01	0.94	0.98	5						
		Mixed	0.97	0.02	0.93	1.00	6	61.00	0.60260	51.50	0.52099	11.00	0.53680
		Healthy	0.82	0.07	0.66	0.89	21						
	**F1**	Clinical	0.75	0.07	0.65	0.83	5						
		Mixed	0.81	0.06	0.73	0.91	7	83.00	0.05092	82.00	0.67120	8.50	0.16672
		Healthy	0.79	0.07	0.61	0.86	21						
	**MCC**	Clinical	0.73	0.07	0.62	0.80	4						
		Mixed	0.80	0.07	0.69	0.91	6	61.00	0.17020	60.50	0.90712	6.00	0.25714
		Healthy	0.92	0.04	0.80	0.98	21						
	**ACC**	Clinical	0.96	0.01	0.95	0.98	5						
**REM**		Mixed	0.96	0.02	0.93	0.99	6	11.50	**0.00838**	20.00	**0.01316**	15.00	1.00000
		Healthy	0.75	0.12	0.34	0.92	21						
	**κ**	Clinical	0.85	0.05	0.78	0.90	4	15.00	**0.04941**	66.00	0.71027	24.00	0.07273

	Mixed	0.76	0.09	0.61	0.89	7						
	Healthy	0.79	0.13	0.46	0.96	22						
**SE**	Clinical	0.87	0.04	0.80	0.93	5						
	Mixed	0.80	0.10	0.63	0.94	7	29.50	0.11854	77.00	1.00000	25.00	0.26768
	Healthy	0.95	0.03	0.88	0.99	21						
**SP**	Clinical	0.98	0.01	0.96	0.99	5						
	Mixed	0.98	0.01	0.95	1.00	7	18.00	**0.02686**	26.50	**0.01358**	16.50	0.93517
	Healthy	0.79	0.11	0.47	0.93	21						
**PPV**	Clinical	0.85	0.07	0.76	0.96	5						
	Mixed	0.80	0.08	0.62	0.89	7	33.00	0.21633	66.00	0.71031	23.00	0.43182
	Healthy	0.96	0.03	0.87	0.99	21						
**NPV**	Clinical	0.98	0.01	0.97	0.99	5						
	Mixed	0.98	0.01	0.96	1.00	6	15.00	**0.01597**	18.50	**0.01023**	13.00	0.79221
	Healthy	0.79	0.10	0.47	0.93	21						
**F1**	Clinical	0.86	0.05	0.81	0.91	5						
	Mixed	0.79	0.08	0.65	0.89	7	27.00	0.10535	71.00	0.91747	27.50	0.12223
	Healthy	0.75	0.12	0.34	0.92	21						
**MCC**	Clinical	0.85	0.05	0.78	0.90	4						
	Mixed	0.77	0.09	0.61	0.89	6	15.00	**0.04945**	51.50	0.52105	20.00	0.11429

ACC: accuracy, κ: Cohen's Kappa, MCC: Matthew's Correlation Coefficient, NPV: negative predictive value, PPV: positive predictive value, SE: sensitivity, SP: specificity.

Supplementary Table S6. Influence of study environment (controlled vs home) on evaluation metrics in studies classifying 5 stages. Significant *p*-values are highlighted in bold.

		Metric	Type	Stats					Dry vs wet electrodes	
				**Mean**	**SD**	**Min**	**Max**	**Count**	**U-stat**	***p*-value**
Overall	Micro-averaged	Multiclass MCC	Controlled	0.71	0.08	0.53	0.90	19		
			Home	0.70	0.08	0.45	0.78	12	108.00	0.91586
		κ	Controlled	0.70	0.09	0.47	0.90	22		
			Home	0.68	0.10	0.42	0.78	14	118.50	0.69761
		ACC	Controlled	0.80	0.06	0.66	0.95	20		
			Home	0.76	0.07	0.59	0.84	14	114.50	0.85005
		SE	Controlled	0.80	0.06	0.66	0.95	19		
			Home	0.78	0.06	0.60	0.84	12	110.50	0.83258
		SP	Controlled	0.95	0.02	0.92	0.99	19		
			Home	0.95	0.02	0.90	0.96	12	98.00	0.78331
		PPV	Controlled	0.80	0.06	0.66	0.95	19		
			Home	0.78	0.06	0.60	0.84	12	110.50	0.83258
		NPV	Controlled	0.95	0.02	0.92	0.99	19		
			Home	0.95	0.02	0.90	0.96	12	98.00	0.78331
		F1	Controlled	0.80	0.06	0.66	0.95	19		
			Home	0.78	0.06	0.60	0.84	12	110.50	0.83258
	Macro-averaged	ACC	Controlled	0.92	0.03	0.87	0.98	19		
			Home	0.91	0.02	0.84	0.94	12	100.50	0.86564
		κ	Controlled	0.67	0.08	0.45	0.86	19		
			Home	0.65	0.09	0.40	0.77	12	143.00	0.11304
		SE	Controlled	0.73	0.07	0.55	0.89	19		
			Home	0.71	0.09	0.52	0.80	12	134.00	0.22841
		SP	Controlled	0.94	0.02	0.91	0.98	19		
			Home	0.94	0.02	0.89	0.96	12	102.00	0.91578
		PPV	Controlled	0.73	0.07	0.56	0.86	19		
			Home	0.73	0.08	0.51	0.83	12	121.50	0.49888
		NPV	Controlled	0.94	0.02	0.91	0.98	19		
			Home	0.94	0.02	0.89	0.96	12	93.00	0.62668
		F1	Controlled	0.72	0.07	0.54	0.88	19		
			Home	0.71	0.08	0.50	0.81	11	131.00	0.10323
		MCC	Controlled	0.67	0.08	0.46	0.86	19		
			Home	0.66	0.09	0.40	0.77	12	138.00	0.16964
Wake		ACC	Controlled	0.94	0.03	0.89	0.98	20		
			Home	0.94	0.04	0.84	0.98	12	83.50	0.29031
		κ	Controlled	0.77	0.11	0.42	0.95	20		
			Home	0.78	0.09	0.52	0.88	12	114.00	0.88684
		SE	Controlled	0.80	0.12	0.40	0.98	22		
			Home	0.83	0.10	0.53	0.92	12	116.00	0.89473
		SP	Controlled	0.96	0.02	0.91	0.99	21		
			Home	0.96	0.02	0.90	0.99	12	86.00	0.26340
		PPV	Controlled	0.83	0.11	0.47	0.98	21		
			Home	0.82	0.07	0.70	0.93	12	152.00	0.15274
		NPV	Controlled	0.95	0.02	0.89	0.99	20		
			Home	0.96	0.03	0.86	1.00	12	70.50	0.11274
		F1	Controlled	0.81	0.11	0.46	0.98	21		
			Home	0.82	0.08	0.61	0.90	12	131.00	0.54373
		MCC	Controlled	0.77	0.11	0.42	0.95	19		
			Home	0.78	0.09	0.53	0.88	12	112.00	0.78348
N1		ACC	Controlled	0.91	0.04	0.78	0.97	20		
			Home	0.92	0.03	0.86	0.99	12	49.00	**0.01390**
		κ	Controlled	0.38	0.15	0.07	0.65	20	134.00	0.33928

		Home	0.39	0.13	0.04	0.56	12		
		Controlled	0.45	0.18	0.14	0.73	22		
	**SE**	Home	0.47	0.15	0.05	0.63	12	154.00	0.20545
		Controlled	0.95	0.04	0.79	0.99	21		
	**SP**	Home	0.96	0.03	0.92	1.00	12	69.50	0.07781
		Controlled	0.45	0.15	0.07	0.78	21		
	**PPV**	Home	0.51	0.12	0.04	0.64	12	102.00	0.62437
		Controlled	0.95	0.03	0.85	0.99	20		
	**NPV**	Home	0.96	0.02	0.91	0.99	12	61.50	0.05089
		Controlled	0.42	0.16	0.11	0.66	21		
	**F1**	Home	0.43	0.14	0.07	0.61	12	155.50	0.11710
		Controlled	0.39	0.15	0.09	0.65	19		
	**MCC**	Home	0.39	0.12	0.05	0.56	12	126.50	0.37481
		Controlled	0.86	0.05	0.77	0.97	20		
	**ACC**	Home	0.83	0.04	0.74	0.89	12	155.00	0.07019
		Controlled	0.71	0.09	0.52	0.91	20		
	**κ**	Home	0.66	0.08	0.47	0.77	12	152.00	0.09156
		Controlled	0.83	0.07	0.69	0.94	22		
	**SE**	Home	0.81	0.09	0.58	0.91	12	131.00	0.69142
		Controlled	0.87	0.06	0.73	0.99	21		
**N2**	**SP**	Home	0.85	0.06	0.71	0.98	12	153.00	0.14184
		Controlled	0.83	0.08	0.59	0.95	21		
	**PPV**	Home	0.80	0.07	0.70	0.94	12	151.50	0.15837
		Controlled	0.88	0.06	0.74	0.97	20		
	**NPV**	Home	0.87	0.05	0.77	0.94	12	131.00	0.40459
		Controlled	0.83	0.05	0.70	0.93	21		
	**F1**	Home	0.80	0.06	0.70	0.86	12	151.50	0.15840
		Controlled	0.72	0.09	0.54	0.91	19		
	**MCC**	Home	0.66	0.08	0.47	0.77	12	149.00	0.06598
		Controlled	0.95	0.03	0.88	0.99	20		
	**ACC**	Home	0.94	0.02	0.91	0.96	12	133.50	0.34966
		Controlled	0.76	0.09	0.41	0.91	20		
	**κ**	Home	0.80	0.07	0.61	0.86	12	60.00	**0.04417**
		Controlled	0.78	0.10	0.58	0.98	22		
	**SE**	Home	0.84	0.08	0.74	1.00	12	65.00	**0.03939**
		Controlled	0.97	0.01	0.95	0.99	21		
**N3**	**SP**	Home	0.96	0.02	0.91	0.99	12	154.00	0.13106
		Controlled	0.82	0.08	0.57	0.90	21		
	**PPV**	Home	0.84	0.08	0.59	0.95	12	68.50	0.07143
		Controlled	0.96	0.03	0.89	1.00	20		
	**NPV**	Home	0.97	0.02	0.93	1.00	12	117.50	0.77590
		Controlled	0.79	0.07	0.65	0.91	21		
	**F1**	Home	0.84	0.06	0.66	0.89	12	49.00	**0.01028**
		Controlled	0.77	0.07	0.62	0.91	19		
	**MCC**	Home	0.80	0.07	0.61	0.86	12	57.50	**0.04698**
		Controlled	0.94	0.03	0.85	0.99	20		
	**ACC**	Home	0.93	0.04	0.80	0.98	12	167.50	**0.02045**
		Controlled	0.77	0.09	0.57	0.92	20		
	**κ**	Home	0.75	0.13	0.34	0.90	12	136.50	0.29040
		Controlled	0.82	0.09	0.53	0.94	22		
**REM**	**SE**	Home	0.77	0.14	0.46	0.96	12	157.00	0.16765
		Controlled	0.96	0.03	0.89	1.00	21		
	**SP**	Home	0.96	0.03	0.88	0.99	12	151.50	0.15826
		Controlled	0.79	0.09	0.55	0.93	21		
	**PPV**	Home	0.82	0.11	0.47	0.96	12	112.50	0.93755
	**NPV**	Controlled	0.97	0.02	0.92	1.00	20	184.50	**0.00261**

	Home	0.95	0.03	0.87	0.99	12		
	Controlled	0.81	0.08	0.63	0.93	21		
**F1**	Home	0.79	0.12	0.47	0.92	12	139.00	0.35724
	Controlled	0.77	0.09	0.58	0.92	19		
**MCC**	Home	0.76	0.13	0.34	0.90	12	134.00	0.22836

ACC: accuracy, κ: Cohen's Kappa, MCC: Matthew's Correlation Coefficient, NPV: negative predictive value, PPV: positive predictive value, SE: sensitivity, SP: specificity.

Supplementary Table S7. Influence of using a commercial or a prototype device on evaluation metrics in studies classifying 5 stages.

		Metric	Type	Stats					Commercial vs prototype	
				Mean	SD	Min	Max	Count	U-stat	*p*-value
Overall	Micro-averaged		Commercial	0.71	0.08	0.53	0.90	19		
		**MCC**	Prototype	0.70	0.08	0.45	0.78	12	129.00	0.55650
			Commercial	0.70	0.09	0.47	0.90	22		
		**κ**	Prototype	0.68	0.10	0.42	0.78	14	173.50	0.61889
			Commercial	0.80	0.06	0.66	0.95	20		
		**ACC**	Prototype	0.76	0.07	0.59	0.84	14	141.00	0.88723
			Commercial	0.80	0.06	0.66	0.95	19		
		**SE**	Prototype	0.78	0.06	0.60	0.84	12	126.00	0.64077
			Commercial	0.95	0.02	0.92	0.99	19		
		**SP**	Prototype	0.95	0.02	0.90	0.96	12	136.00	0.38266
			Commercial	0.80	0.06	0.66	0.95	19		
		**PPV**	Prototype	0.78	0.06	0.60	0.84	12	126.00	0.64077
			Commercial	0.95	0.02	0.92	0.99	19		
		**NPV**	Prototype	0.95	0.02	0.90	0.96	12	136.00	0.38266
			Commercial	0.80	0.06	0.66	0.95	19		
		**F1**	Prototype	0.78	0.06	0.60	0.84	12	126.00	0.64077
	Macro-averaged		Commercial	0.92	0.03	0.87	0.98	19		
		**ACC**	Prototype	0.91	0.02	0.84	0.94	12	133.00	0.45263
			Commercial	0.67	0.08	0.45	0.86	19		
		**κ**	Prototype	0.65	0.09	0.40	0.77	12	112.00	0.95149
			Commercial	0.73	0.07	0.55	0.89	19		
		**SE**	Prototype	0.71	0.09	0.52	0.80	12	126.00	0.64091
			Commercial	0.94	0.02	0.91	0.98	19		
		**SP**	Prototype	0.94	0.02	0.89	0.96	12	136.00	0.38276
			Commercial	0.73	0.07	0.56	0.86	19		
		**PPV**	Prototype	0.73	0.08	0.51	0.83	12	125.50	0.65545
			Commercial	0.94	0.02	0.91	0.98	19		
		**NPV**	Prototype	0.94	0.02	0.89	0.96	12	137.00	0.36107
			Commercial	0.72	0.07	0.54	0.88	19		
		**F1**	Prototype	0.71	0.08	0.50	0.81	11	109.50	0.96623
			Commercial	0.67	0.08	0.46	0.86	19		
		**MCC**	Prototype	0.66	0.09	0.40	0.77	12	116.00	0.95149
Wake			Commercial	0.94	0.03	0.89	0.98	20		
		**ACC**	Prototype	0.94	0.04	0.84	0.98	12	156.50	0.21223
			Commercial	0.77	0.11	0.42	0.95	20		
		**κ**	Prototype	0.78	0.09	0.52	0.88	12	136.00	0.64518
			Commercial	0.80	0.12	0.40	0.98	22		
		**SE**	Prototype	0.83	0.10	0.53	0.92	12	155.00	0.67718
			Commercial	0.96	0.02	0.91	0.99	21		
		**SP**	Prototype	0.96	0.02	0.90	0.99	12	177.00	0.11244
			Commercial	0.83	0.11	0.47	0.98	21		
		**PPV**	Prototype	0.82	0.07	0.70	0.93	12	124.00	0.75683
			Commercial	0.95	0.02	0.89	0.99	20		
		**NPV**	Prototype	0.96	0.03	0.86	1.00	12	167.00	0.09887
			Commercial	0.81	0.11	0.46	0.98	21		
		**F1**	Prototype	0.82	0.08	0.61	0.90	12	126.00	0.81284
			Commercial	0.77	0.11	0.42	0.95	19		
		**MCC**	Prototype	0.78	0.09	0.53	0.88	12	120.00	0.82342
N1			Commercial	0.91	0.04	0.78	0.97	20		
		**ACC**	Prototype	0.92	0.03	0.86	0.99	12	144.00	0.44281
			Commercial	0.38	0.15	0.07	0.65	20		
		**κ**	Prototype	0.39	0.13	0.04	0.56	12	146.00	0.39851
			Commercial	0.45	0.18	0.14	0.73	22		
		**SE**	Prototype	0.47	0.15	0.05	0.63	12	166.00	0.42502
		**SP**	Commercial	0.95	0.04	0.79	0.99	21	140.00	0.81276

		Prototype	0.96	0.03	0.92	1.00	12		
		Commercial	0.45	0.15	0.07	0.78	21		
	**PPV**	Prototype	0.51	0.12	0.04	0.64	12	173.00	0.15020
		Commercial	0.95	0.03	0.85	0.99	20		
	**NPV**	Prototype	0.96	0.02	0.91	0.99	12	132.50	0.74421
		Commercial	0.42	0.16	0.11	0.66	21		
	**F1**	Prototype	0.43	0.14	0.07	0.61	12	153.50	0.46626
		Commercial	0.39	0.15	0.09	0.65	19		
	**MCC**	Prototype	0.39	0.12	0.05	0.56	12	136.00	0.38319
		Commercial	0.86	0.05	0.77	0.97	20		
	**ACC**	Prototype	0.83	0.04	0.74	0.89	12	117.50	0.83273
		Commercial	0.71	0.09	0.52	0.91	20		
	**κ**	Prototype	0.66	0.08	0.47	0.77	12	123.00	1.00000
		Commercial	0.83	0.07	0.69	0.94	22		
	**SE**	Prototype	0.81	0.09	0.58	0.91	12	141.50	0.98616
		Commercial	0.87	0.06	0.73	0.99	21		
**N2**	**SP**	Prototype	0.85	0.06	0.71	0.98	12	132.00	0.98547
		Commercial	0.83	0.08	0.59	0.95	21		
	**PPV**	Prototype	0.80	0.07	0.70	0.94	12	139.50	0.82695
		Commercial	0.88	0.06	0.74	0.97	20		
	**NPV**	Prototype	0.87	0.05	0.77	0.94	12	109.00	0.59115
		Commercial	0.83	0.05	0.70	0.93	21		
	**F1**	Prototype	0.80	0.06	0.70	0.86	12	144.50	0.68860
		Commercial	0.72	0.09	0.54	0.91	19		
	**MCC**	Prototype	0.66	0.08	0.47	0.77	12	106.50	0.77645
		Commercial	0.95	0.03	0.88	0.99	20		
	**ACC**	Prototype	0.94	0.02	0.91	0.96	12	132.00	0.75883
		Commercial	0.76	0.09	0.41	0.91	20		
	**κ**	Prototype	0.80	0.07	0.61	0.86	12	167.00	0.09897
		Commercial	0.78	0.10	0.58	0.98	22		
	**SE**	Prototype	0.84	0.08	0.74	1.00	12	191.00	0.09592
		Commercial	0.97	0.01	0.95	0.99	21		
**N3**	**SP**	Prototype	0.96	0.02	0.91	0.99	12	109.50	0.40159
		Commercial	0.82	0.08	0.57	0.90	21		
	**PPV**	Prototype	0.84	0.08	0.59	0.95	12	169.00	0.19578
		Commercial	0.96	0.03	0.89	1.00	20		
	**NPV**	Prototype	0.97	0.02	0.93	1.00	12	139.50	0.55192
		Commercial	0.79	0.07	0.65	0.91	21		
	**F1**	Prototype	0.84	0.06	0.66	0.89	12	176.50	0.11721
		Commercial	0.77	0.07	0.62	0.91	19		
	**MCC**	Prototype	0.80	0.07	0.61	0.86	12	146.00	0.20134
		Commercial	0.94	0.03	0.85	0.99	20		
	**ACC**	Prototype	0.93	0.04	0.80	0.98	12	111.50	0.65895
		Commercial	0.77	0.09	0.57	0.92	20		
	**κ**	Prototype	0.75	0.13	0.34	0.90	12	137.50	0.60440
		Commercial	0.82	0.09	0.53	0.94	22		
	**SE**	Prototype	0.77	0.14	0.46	0.96	12	148.50	0.84869
		Commercial	0.96	0.03	0.89	1.00	21		
**REM**	**SP**	Prototype	0.96	0.03	0.88	0.99	12	132.50	1.00000
		Commercial	0.79	0.09	0.55	0.93	21		
	**PPV**	Prototype	0.82	0.11	0.47	0.96	12	166.00	0.23644
		Commercial	0.97	0.02	0.92	1.00	20		
	**NPV**	Prototype	0.95	0.03	0.87	0.99	12	91.00	0.21922
		Commercial	0.81	0.08	0.63	0.93	21		
	**F1**	Prototype	0.79	0.12	0.47	0.92	12	148.00	0.59735
		Commercial	0.77	0.09	0.58	0.92	19		
	**MCC**	Prototype	0.76	0.13	0.34	0.90	12	122.50	0.74555

ACC: accuracy, *κ*: Cohen's Kappa, MCC: Matthew's Correlation Coefficient, NPV: negative predictive value, PPV: positive predictive value, SE: sensitivity, SP: specificity.

Supplementary Table S8. Influence of electrode location (forehead vs ear) on evaluation metrics in studies classifying 5 stages. Significant *p*-values are highlighted in bold.

		Metric	Type	Stats					Forehead vs ear	
				Mean	SD	Min	Max	Count	U-stat	*p*-value
Overall	Micro-averaged	MCC	Forehead	0.71	0.08	0.53	0.90	19		
			Ear	0.70	0.08	0.45	0.78	12	114.00	1.00000
		κ	Forehead	0.67	0.08	0.45	0.86	19		
			Ear	0.65	0.09	0.40	0.77	12	166.50	0.69694
		ACC	Forehead	0.92	0.03	0.87	0.98	19		
			Ear	0.91	0.02	0.84	0.94	12	162.50	0.44122
		SE	Forehead	0.80	0.06	0.66	0.95	19		
			Ear	0.78	0.06	0.60	0.84	12	120.50	0.80765
		SP	Forehead	0.95	0.02	0.92	0.99	19		
			Ear	0.95	0.02	0.90	0.96	12	108.00	0.82328
		PPV	Forehead	0.80	0.06	0.66	0.95	19		
			Ear	0.78	0.06	0.60	0.84	12	120.50	0.80765
		NPV	Forehead	0.95	0.02	0.92	0.99	19		
			Ear	0.95	0.02	0.90	0.96	12	108.00	0.82328
		F1	Forehead	0.80	0.06	0.66	0.95	19		
			Ear	0.78	0.06	0.60	0.84	12	120.50	0.80765
	Macro-averaged	ACC	Forehead	0.92	0.03	0.87	0.98	19		
			Ear	0.91	0.02	0.84	0.94	12	111.50	0.93529
		κ	Forehead	0.67	0.08	0.45	0.86	19		
			Ear	0.65	0.09	0.40	0.77	12	133.00	0.45309
		SE	Forehead	0.73	0.07	0.55	0.89	19		
			Ear	0.71	0.09	0.52	0.80	12	120.00	0.82348
		SP	Forehead	0.94	0.02	0.91	0.98	19		
			Ear	0.94	0.02	0.89	0.96	12	105.50	0.74535
		PPV	Forehead	0.73	0.07	0.56	0.86	19		
			Ear	0.73	0.08	0.51	0.83	12	113.50	1.00000
		NPV	Forehead	0.94	0.02	0.91	0.98	19		
			Ear	0.94	0.02	0.89	0.96	12	104.00	0.69977
		F1	Forehead	0.72	0.07	0.54	0.88	19		
			Ear	0.71	0.08	0.50	0.81	11	120.00	0.51853
		MCC	Forehead	0.67	0.08	0.46	0.86	19		
			Ear	0.66	0.09	0.40	0.77	12	127.00	0.61220
Wake		ACC	Forehead	0.94	0.03	0.89	0.98	20		
			Ear	0.94	0.04	0.84	0.98	12	99.00	0.42472
		κ	Forehead	0.77	0.11	0.42	0.95	20		
			Ear	0.78	0.09	0.52	0.88	12	104.00	0.54625
		SE	Forehead	0.80	0.12	0.40	0.98	22		
			Ear	0.83	0.10	0.53	0.92	12	108.00	0.39695
		SP	Forehead	0.96	0.02	0.91	0.99	21		
			Ear	0.96	0.02	0.90	0.99	12	106.00	0.46475
		PPV	Forehead	0.83	0.11	0.47	0.98	21		
			Ear	0.82	0.07	0.70	0.93	12	144.00	0.51248
		NPV	Forehead	0.95	0.02	0.89	0.99	20		
			Ear	0.96	0.03	0.86	1.00	12	86.00	0.19212
		F1	Forehead	0.81	0.11	0.46	0.98	21		
			Ear	0.82	0.08	0.61	0.90	12	115.00	0.69435
		MCC	Forehead	0.77	0.11	0.42	0.95	19		
			Ear	0.78	0.09	0.53	0.88	12	102.00	0.64080
N1		ACC	Forehead	0.91	0.04	0.78	0.97	20		
			Ear	0.92	0.03	0.86	0.99	12	96.00	0.36029
		κ	Forehead	0.38	0.15	0.07	0.65	20		
			Ear	0.39	0.13	0.04	0.56	12	112.50	0.78522
		SE	Forehead	0.45	0.18	0.14	0.73	22		
			Ear	0.47	0.15	0.05	0.63	12	127.00	0.87117
		SP	Forehead	0.95	0.04	0.79	0.99	21		
			Ear	0.96	0.03	0.92	1.00	12	119.50	0.82226

		Forehead	0.45	0.15	0.07	0.78	21		
	**PPV**	Ear	0.51	0.12	0.04	0.64	12	92.00	0.20995
		Forehead	0.95	0.03	0.85	0.99	20		
	**NPV**	Ear	0.96	0.02	0.91	0.99	12	98.50	0.41347
		Forehead	0.42	0.16	0.11	0.66	21		
	**F1**	Ear	0.43	0.14	0.07	0.61	12	127.50	0.97014
		Forehead	0.39	0.15	0.09	0.65	19		
	**MCC**	Ear	0.39	0.12	0.05	0.56	12	104.50	0.71509
		Forehead	0.86	0.05	0.77	0.97	20		
	**ACC**	Ear	0.83	0.04	0.74	0.89	12	158.50	0.13877
		Forehead	0.71	0.09	0.52	0.91	20		
	**κ**	Ear	0.66	0.08	0.47	0.77	12	156.00	0.16702
		Forehead	0.83	0.07	0.69	0.94	22		
	**SE**	Ear	0.81	0.09	0.58	0.91	12	154.00	0.43838
		Forehead	0.87	0.06	0.73	0.99	21		
**N2**	**SP**	Ear	0.85	0.06	0.71	0.98	12	152.00	0.33992
		Forehead	0.83	0.08	0.59	0.95	21		
	**PPV**	Ear	0.80	0.07	0.70	0.94	12	166.50	0.13431
		Forehead	0.88	0.06	0.74	0.97	20		
	**NPV**	Ear	0.87	0.05	0.77	0.94	12	138.00	0.49575
		Forehead	0.83	0.05	0.70	0.93	21		
	**F1**	Ear	0.80	0.06	0.70	0.86	12	162.50	0.17782
		Forehead	0.72	0.09	0.54	0.91	19		
	**MCC**	Ear	0.66	0.08	0.47	0.77	12	155.50	0.09629
		Forehead	0.95	0.03	0.88	0.99	20		
	**ACC**	Ear	0.94	0.02	0.91	0.96	12	139.50	0.45948
		Forehead	0.76	0.09	0.41	0.91	20		
	**κ**	Ear	0.80	0.07	0.61	0.86	12	78.00	0.10623
		Forehead	0.78	0.10	0.58	0.98	22		
	**SE**	Ear	0.84	0.08	0.74	1.00	12	90.00	0.13474
		Forehead	0.97	0.01	0.95	0.99	21		
**N3**	**SP**	Ear	0.96	0.02	0.91	0.99	12	160.50	0.20270
		Forehead	0.82	0.08	0.57	0.90	21		
	**PPV**	Ear	0.84	0.08	0.59	0.95	12	88.50	0.16597
		Forehead	0.96	0.03	0.89	1.00	20		
	**NPV**	Ear	0.97	0.02	0.93	1.00	12	129.50	0.72602
		Forehead	0.79	0.07	0.65	0.91	21		
	**F1**	Ear	0.84	0.06	0.66	0.89	12	73.50	0.05161
		Forehead	0.77	0.07	0.62	0.91	19		
	**MCC**	Ear	0.80	0.07	0.61	0.86	12	78.00	0.14987
		Forehead	0.94	0.03	0.85	0.99	20		
	**ACC**	Ear	0.93	0.04	0.80	0.98	12	148.00	0.28429
		Forehead	0.77	0.09	0.57	0.92	20		
	**κ**	Ear	0.75	0.13	0.34	0.90	12	135.00	0.57240
		Forehead	0.82	0.09	0.53	0.94	22		
	**SE**	Ear	0.77	0.14	0.46	0.96	12	165.50	0.23427
		Forehead	0.96	0.03	0.89	1.00	21		
**REM**	**SP**	Ear	0.96	0.03	0.88	0.99	12	125.50	1.00000
		Forehead	0.79	0.09	0.55	0.93	21		
	**PPV**	Ear	0.82	0.11	0.47	0.96	12	104.50	0.43188
		Forehead	0.97	0.02	0.92	1.00	20		
	**NPV**	Ear	0.95	0.03	0.87	0.99	12	175.50	**0.03217**
		Forehead	0.81	0.08	0.63	0.93	21		
	**F1**	Ear	0.79	0.12	0.47	0.92	12	141.00	0.58734
		Forehead	0.77	0.09	0.58	0.92	19		
	**MCC**	Ear	0.76	0.13	0.34	0.90	12	131.50	0.49046

ACC: accuracy, *κ*: Cohen's Kappa, MCC: Matthew's Correlation Coefficient, NPV: negative predictive value, PPV: positive predictive value, SE: sensitivity, SP: specificity.

Supplementary Table S9. Influence of using dry vs wet electrodes on evaluation metrics in studies classifying 5 stages. Significant *p*-values are highlighted in bold.

	Metric	Type	Stats					Dry vs wet electrodes	
			Mean	SD	Min	Max	Count	U-stat	*p*-value
Overall	Multiclass MCC	Dry	0.71	0.08	0.53	0.90	19		
		Wet	0.70	0.08	0.45	0.78	12	127.00	0.36359
	κ	Dry	0.70	0.09	0.47	0.90	22		
		Wet	0.68	0.10	0.42	0.78	14	211.50	0.06433
Overall, Micro-averaged	ACC	Dry	0.80	0.06	0.66	0.95	20		
		Wet	0.76	0.07	0.59	0.84	14	178.00	0.10094
	SE	Dry	0.80	0.06	0.66	0.95	19		
		Wet	0.78	0.06	0.60	0.84	12	132.00	0.26255
	SP	Dry	0.95	0.02	0.92	0.99	19		
		Wet	0.95	0.02	0.90	0.96	12	118.50	0.58231
	PPV	Dry	0.80	0.06	0.66	0.95	19		
		Wet	0.78	0.06	0.60	0.84	12	132.00	0.26255
	NPV	Dry	0.95	0.02	0.92	0.99	19		
		Wet	0.95	0.02	0.90	0.96	12	118.50	0.58231
	F1	Dry	0.80	0.06	0.66	0.95	19		
		Wet	0.78	0.06	0.60	0.84	12	132.00	0.26255
Overall, Macro-averaged	ACC	Dry	0.92	0.03	0.87	0.98	19		
		Wet	0.91	0.02	0.84	0.94	12	121.50	0.49853
	κ	Dry	0.67	0.08	0.45	0.86	19		
		Wet	0.65	0.09	0.40	0.77	12	105.00	1.00000
	SE	Dry	0.73	0.07	0.55	0.89	19		
		Wet	0.71	0.09	0.52	0.80	12	111.50	0.79983
	SP	Dry	0.94	0.02	0.91	0.98	19		
		Wet	0.94	0.02	0.89	0.96	12	112.50	0.76715
	PPV	Dry	0.73	0.07	0.56	0.86	19		
		Wet	0.73	0.08	0.51	0.83	12	94.50	0.67254
	NPV	Dry	0.94	0.02	0.91	0.98	19		
		Wet	0.94	0.02	0.89	0.96	12	113.00	0.75108
	F1	Dry	0.72	0.07	0.54	0.88	19		
		Wet	0.71	0.08	0.50	0.81	11	85.00	0.68375
	MCC	Dry	0.67	0.08	0.46	0.86	19		
		Wet	0.66	0.09	0.40	0.77	12	102.00	0.91586
Wake	ACC	Dry	0.94	0.03	0.89	0.98	20		
		Wet	0.94	0.04	0.84	0.98	12	138.00	0.38256
	κ	Dry	0.77	0.11	0.42	0.95	20		
		Wet	0.78	0.09	0.52	0.88	12	116.00	0.82305
	SE	Dry	0.80	0.12	0.40	0.98	22		
		Wet	0.83	0.10	0.53	0.92	12	161.50	0.29587
	SP	Dry	0.96	0.02	0.91	0.99	21		
		Wet	0.96	0.02	0.90	0.99	12	137.00	0.55320
	PPV	Dry	0.83	0.11	0.47	0.98	21		
		Wet	0.82	0.07	0.70	0.93	12	130.50	0.73105
	NPV	Dry	0.95	0.02	0.89	0.99	20		
		Wet	0.96	0.03	0.86	1.00	12	129.00	0.60590
	F1	Dry	0.81	0.11	0.46	0.98	21		
		Wet	0.82	0.08	0.61	0.90	12	140.00	0.47987
	MCC	Dry	0.77	0.11	0.42	0.95	19		
		Wet	0.78	0.09	0.53	0.88	12	114.00	0.71934
N1	ACC	Dry	0.91	0.04	0.78	0.97	20		
		Wet	0.92	0.03	0.86	0.99	12	142.00	0.30223

		Dry	0.38	0.15	0.07	0.65	20		
	**κ**	Wet	0.39	0.13	0.04	0.56	12	65.50	0.07359
		Dry	0.45	0.18	0.14	0.73	22		
	**SE**	Wet	0.47	0.15	0.05	0.63	12	103.00	0.30439
		Dry	0.95	0.04	0.79	0.99	21		
	**SP**	Wet	0.96	0.03	0.92	1.00	12	152.50	0.23627
		Dry	0.45	0.15	0.07	0.78	21		
	**PPV**	Wet	0.51	0.12	0.04	0.64	12	75.00	0.08228
		Dry	0.95	0.03	0.85	0.99	20		
	**NPV**	Wet	0.96	0.02	0.91	0.99	12	131.00	0.55157
		Dry	0.42	0.16	0.11	0.66	21		
	**F1**	Wet	0.43	0.14	0.07	0.61	12	77.50	0.10053
		Dry	0.39	0.15	0.09	0.65	19		
	**MCC**	Wet	0.39	0.12	0.05	0.56	12	63.00	0.07945
**N2**		Dry	0.86	0.05	0.77	0.97	20		
	**ACC**	Wet	0.83	0.04	0.74	0.89	12	135.00	0.45057
		Dry	0.71	0.09	0.52	0.91	20		
	**κ**	Wet	0.66	0.08	0.47	0.77	12	126.00	0.52859
		Dry	0.83	0.07	0.69	0.94	22		
	**SE**	Wet	0.81	0.09	0.58	0.91	12	146.50	0.61384
		Dry	0.87	0.06	0.73	0.99	21		
	**SP**	Wet	0.85	0.06	0.71	0.98	12	157.00	0.17519
		Dry	0.83	0.08	0.59	0.95	21		
	**PPV**	Wet	0.80	0.07	0.70	0.94	12	178.00	**0.03091**
		Dry	0.88	0.06	0.74	0.97	20		
	**NPV**	Wet	0.87	0.05	0.77	0.94	12	107.00	0.75093
		Dry	0.83	0.05	0.70	0.93	21		
	**F1**	Wet	0.80	0.06	0.70	0.86	12	150.00	0.27634
		Dry	0.72	0.09	0.54	0.91	19		
	**MCC**	Wet	0.66	0.08	0.47	0.77	12	126.50	0.37476
**N3**		Dry	0.95	0.03	0.88	0.99	20		
	**ACC**	Wet	0.94	0.02	0.91	0.96	12	154.00	0.13156
		Dry	0.76	0.09	0.41	0.91	20		
	**κ**	Wet	0.80	0.07	0.61	0.86	12	126.00	0.52859
		Dry	0.78	0.10	0.58	0.98	22		
	**SE**	Wet	0.84	0.08	0.74	1.00	12	183.00	0.06875
		Dry	0.97	0.01	0.95	0.99	21		
	**SP**	Wet	0.96	0.02	0.91	0.99	12	127.00	0.83344
		Dry	0.82	0.08	0.57	0.90	21		
	**PPV**	Wet	0.84	0.08	0.59	0.95	12	101.00	0.45627
		Dry	0.96	0.03	0.89	1.00	20		
	**NPV**	Wet	0.97	0.02	0.93	1.00	12	169.50	**0.03373**
		Dry	0.79	0.07	0.65	0.91	21		
	**F1**	Wet	0.84	0.06	0.66	0.89	12	144.00	0.39012
		Dry	0.77	0.07	0.62	0.91	19		
	**MCC**	Wet	0.80	0.07	0.61	0.86	12	128.50	0.33099
**REM**		Dry	0.94	0.03	0.85	0.99	20		
	**ACC**	Wet	0.93	0.04	0.80	0.98	12	118.50	0.92097
		Dry	0.77	0.09	0.57	0.92	20		
	**κ**	Wet	0.75	0.13	0.34	0.90	12	134.00	0.33928
		Dry	0.82	0.09	0.53	0.94	22		
	**SE**	Wet	0.77	0.14	0.46	0.96	12	182.50	0.07152
		Dry	0.96	0.03	0.89	1.00	21		
	**SP**	Wet	0.96	0.03	0.88	0.99	12	87.00	0.20055
		Dry	0.79	0.09	0.55	0.93	21		
	**PPV**	Wet	0.82	0.11	0.47	0.96	12	111.00	0.71673

	Dry	0.97	0.02	0.92	1.00	20		
**NPV**	Wet	0.95	0.03	0.87	0.99	12	146.00	0.23364
	Dry	0.81	0.08	0.63	0.93	21		
**F1**	Wet	0.79	0.12	0.47	0.92	12	144.50	0.37971
	Dry	0.77	0.09	0.58	0.92	19		
**MCC**	Wet	0.76	0.13	0.34	0.90	12	128.00	0.34161

ACC: accuracy, κ: Cohen's Kappa, MCC: Matthew's Correlation Coefficient, NPV: negative predictive value, PPV: positive predictive value, SE: sensitivity, SP: specificity.

Supplementary Table S10. Influence of device scoring methods on evaluation metrics in studies classifying 5 stages. Significant *p*-values are highlighted in bold.

	Metric	Type	Stats					Machine learning vs Manual scoring		Machine learning vs Proprietary algorithm		Machine learning vs Deep learning		Manual scoring vs Proprietary algorithm		Manual scoring vs Deep learning		Proprietary algorithm vs Deep learning	
			Mean	SD	Min	Max	Count	U-stat	*p*-value	U-stat	*p*-value	U-stat	*p*-value	U-stat	*p*-value	U-stat	*p*-value	U-stat	*p*-value
Overall	Multiclass MCC	Machine learning	0.68	0.09	0.45	0.77	10												
		Manual scoring	0.73	0.08	0.62	0.90	9												
		Proprietary algorithm	0.72	0.08	0.59	0.80	4												
		Deep learning	0.73	0.05	0.63	0.78	7	34.00	0.39127	14.00	0.45355	19.00	0.13307	18.00	1.00000	24.00	0.46976	14.00	1.00000
	κ	Machine learning	0.67	0.09	0.45	0.77	11												
		Manual scoring	0.71	0.10	0.42	0.90	11												
		Proprietary algorithm	0.68	0.12	0.47	0.80	5												
		Deep learning	0.72	0.05	0.61	0.78	8	49.00	0.47010	25.00	0.82692	23.50	0.09850	30.00	0.82692	33.50	0.40876	17.00	0.72416
	ACC	Machine learning	0.76	0.07	0.60	0.84	11												
		Manual scoring	0.79	0.08	0.59	0.95	11												
		Proprietary algorithm	0.80	0.07	0.69	0.89	4												
		Deep learning	0.81	0.03	0.73	0.84	7	49.50	0.49040	15.00	0.41172	17.50	0.06323	20.00	0.85128	24.00	0.21091	13.00	0.92727
Micro-averaged	SE	Machine learning	0.76	0.07	0.60	0.84	10												
		Manual scoring	0.81	0.06	0.74	0.95	9												
		Proprietary algorithm	0.80	0.07	0.69	0.89	4												
		Deep learning	0.81	0.03	0.73	0.84	7	33.50	0.36890	14.00	0.45355	17.50	0.09690	18.00	1.00000	23.00	0.40787	13.00	0.92727
	SP	Machine learning	0.94	0.02	0.90	0.96	10												
		Manual scoring	0.95	0.01	0.93	0.99	9												
		Proprietary algorithm	0.95	0.02	0.92	0.97	4												
		Deep learning	0.95	0.01	0.93	0.96	7	27.50	0.16457	14.50	0.47853	17.50	0.09588	19.00	0.93842	28.00	0.75047	13.00	0.92455
	PPV	Machine learning	0.76	0.07	0.60	0.84	10	33.50	0.36890	14.00	0.45355	17.50	0.09690	18.00	1.00000	23.00	0.40787	13.00	0.92727

Macro-averaged		Manual scoring	0.81	0.06	0.74	0.95	9												
		Proprietary algorithm	0.80	0.07	0.69	0.89	4												
		Deep learning	0.81	0.03	0.73	0.84	7												
		Machine learning	0.94	0.02	0.90	0.96	10												
	NPV	Manual scoring	0.95	0.01	0.93	0.99	9												
		Proprietary algorithm	0.95	0.02	0.92	0.97	4												
		Deep learning	0.95	0.01	0.93	0.96	7	27.50	0.16457	14.50	0.47853	17.50	0.09588	19.00	0.93842	28.00	0.75047	13.00	0.92455
		Machine learning	0.76	0.07	0.60	0.84	10												
	F1	Manual scoring	0.81	0.06	0.74	0.95	9												
		Proprietary algorithm	0.80	0.07	0.69	0.89	4												
		Deep learning	0.81	0.03	0.73	0.84	7	33.50	0.36890	14.00	0.45355	17.50	0.09690	18.00	1.00000	23.00	0.40787	13.00	0.92727
		Machine learning	0.90	0.03	0.84	0.94	10												
	ACC	Manual scoring	0.93	0.02	0.90	0.98	9												
		Proprietary algorithm	0.92	0.03	0.88	0.96	4												
		Deep learning	0.92	0.01	0.89	0.94	7	28.00	0.17753	14.00	0.45355	17.50	0.09609	19.00	0.93986	26.00	0.59636	13.00	0.92455
		Machine learning	0.61	0.09	0.40	0.69	10												
	κ	Manual scoring	0.69	0.10	0.52	0.86	9												
		Proprietary algorithm	0.67	0.08	0.54	0.77	4												
		Deep learning	0.69	0.03	0.65	0.75	7	21.00	0.05501	13.00	0.37363	12.00	**0.02499**	18.00	1.00000	31.00	1.00000	12.00	0.78788
		Machine learning	0.69	0.09	0.52	0.78	10												
	SE	Manual scoring	0.75	0.08	0.61	0.89	9												
		Proprietary algorithm	0.72	0.07	0.61	0.80	4												
		Deep learning	0.75	0.03	0.70	0.79	7	29.50	0.22047	14.00	0.45355	21.00	0.19323	21.00	0.71049	29.00	0.83706	11.00	0.64848
		Machine learning	0.93	0.02	0.89	0.96	10												
	SP	Manual scoring	0.95	0.02	0.93	0.98	9												
		Proprietary algorithm	0.94	0.02	0.92	0.97	4												
		Deep learning	0.95	0.01	0.93	0.96	7	28.00	0.17734	14.00	0.45355	20.00	0.15654	19.00	0.93842	25.50	0.55900	14.00	1.00000

	PPV	Machine learning	0.70	0.07	0.51	0.76	10												
		Manual scoring	0.75	0.08	0.61	0.86	9												
		Proprietary algorithm	0.74	0.06	0.65	0.83	4												
		Deep learning	0.75	0.03	0.71	0.81	7	26.00	0.13074	18.00	0.83916	14.50	0.05082	19.00	0.93842	30.00	0.91564	11.00	0.64848
	NPV	Machine learning	0.93	0.02	0.89	0.95	10												
		Manual scoring	0.95	0.02	0.92	0.98	9												
		Proprietary algorithm	0.95	0.02	0.92	0.97	4												
		Deep learning	0.95	0.01	0.92	0.96	7	28.00	0.17677	14.00	0.43617	20.00	0.15680	19.00	0.93833	32.00	1.00000	14.00	1.00000
	F1	Machine learning	0.67	0.08	0.50	0.73	10												
		Manual scoring	0.75	0.08	0.60	0.88	8												
		Proprietary algorithm	0.72	0.07	0.62	0.81	4												
		Deep learning	0.74	0.03	0.71	0.80	7	14.00	**0.02340**	12.00	0.30370	11.00	**0.01851**	20.00	0.55152	32.00	0.68518	11.00	0.64848
	MCC	Machine learning	0.62	0.09	0.40	0.69	10												
		Manual scoring	0.69	0.09	0.53	0.86	9												
		Proprietary algorithm	0.68	0.08	0.55	0.77	4												
		Deep learning	0.69	0.03	0.66	0.75	7	23.00	0.07918	13.00	0.37363	14.00	**0.04309**	18.00	1.00000	32.00	1.00000	12.00	0.78788
Wake	ACC	Machine learning	0.93	0.04	0.84	0.98	10												
		Manual scoring	0.95	0.02	0.92	0.98	9												
		Proprietary algorithm	0.92	0.03	0.89	0.96	4												
		Deep learning	0.94	0.03	0.89	0.98	8	42.50	0.87023	26.00	0.45355	36.50	0.78970	24.50	0.35388	36.00	1.00000	10.00	0.36768
	κ	Machine learning	0.74	0.12	0.42	0.85	10												
		Manual scoring	0.78	0.11	0.44	0.95	10												
		Proprietary algorithm	0.81	0.08	0.73	0.91	4												
		Deep learning	0.79	0.05	0.74	0.88	7	40.00	0.47268	12.00	0.30370	28.00	0.53620	18.00	0.83916	28.00	0.53620	13.00	0.92727
	SE	Machine learning	0.80	0.13	0.44	0.91	10												
		Manual scoring	0.83	0.13	0.40	0.98	10												
		Proprietary algorithm	0.80	0.12	0.60	0.97	5	44.50	0.70525	28.00	0.75925	43.00	0.82412	29.00	0.67865	44.00	0.76183	17.00	0.72416

		Deep learning	0.80	0.06	0.70	0.92	8												
	SP	Machine learning	0.96	0.02	0.90	0.99	10												
		Manual scoring	0.97	0.01	0.94	0.98	10												
		Proprietary algorithm	0.95	0.02	0.93	0.98	4												
		Deep learning	0.97	0.03	0.91	0.99	8	40.00	0.47151	25.00	0.53946	32.00	0.50428	28.00	0.28778	36.50	0.78896	10.00	0.36768
	PPV	Machine learning	0.77	0.11	0.47	0.91	10												
		Manual scoring	0.84	0.10	0.57	0.98	10												
		Proprietary algorithm	0.90	0.05	0.82	0.96	4												
		Deep learning	0.83	0.04	0.76	0.88	8	26.00	0.07566	5.00	**0.03596**	23.00	0.14242	11.00	0.23976	43.00	0.82412	28.00	0.05039
	NPV	Machine learning	0.96	0.04	0.86	1.00	10												
		Manual scoring	0.96	0.02	0.92	0.99	9												
		Proprietary algorithm	0.93	0.02	0.89	0.95	4												
		Deep learning	0.96	0.02	0.93	0.99	8	50.00	0.71330	32.00	0.10589	42.50	0.85888	31.50	**0.04457**	35.00	0.96257	2.00	**0.01616**
	F1	Machine learning	0.78	0.11	0.46	0.87	10												
		Manual scoring	0.82	0.11	0.47	0.98	10												
		Proprietary algorithm	0.87	0.07	0.79	0.97	4												
		Deep learning	0.81	0.04	0.76	0.90	8	34.00	0.24132	11.00	0.23976	33.00	0.57260	17.00	0.73327	45.00	0.69647	23.00	0.28283
	MCC	Machine learning	0.74	0.12	0.42	0.85	10												
		Manual scoring	0.79	0.12	0.45	0.95	9												
		Proprietary algorithm	0.81	0.07	0.73	0.91	4												
		Deep learning	0.79	0.05	0.74	0.88	7	34.00	0.39127	12.00	0.30370	28.00	0.53620	18.00	1.00000	30.50	0.95776	13.00	0.92727
N1	ACC	Machine learning	0.92	0.04	0.86	0.99	10												
		Manual scoring	0.90	0.03	0.84	0.96	9												
		Proprietary algorithm	0.90	0.03	0.87	0.93	4												
		Deep learning	0.92	0.05	0.78	0.97	8	54.00	0.48767	26.00	0.45355	35.00	0.69647	21.00	0.71049	22.50	0.21068	9.00	0.28283
	κ	Machine learning	0.24	0.08	0.04	0.33	10												
		Manual scoring	0.44	0.14	0.14	0.65	10	20.00	**0.02569**	14.00	0.43617	8.00	**0.00966**	25.00	0.53946	35.50	1.00000	9.00	0.41212

		Proprietary algorithm	0.39	0.16	0.14	0.56	4												
		Deep learning	0.41	0.08	0.23	0.47	7												
	SE	Machine learning	0.37	0.14	0.05	0.52	10												
		Manual scoring	0.51	0.15	0.16	0.70	10												
		Proprietary algorithm	0.40	0.17	0.14	0.60	5												
		Deep learning	0.50	0.17	0.17	0.73	8	25.00	0.06402	22.00	0.76790	24.00	0.17281	35.00	0.25441	44.00	0.76183	14.00	0.43512
	SP	Machine learning	0.96	0.03	0.91	1.00	10												
		Manual scoring	0.95	0.02	0.92	0.99	10												
		Proprietary algorithm	0.95	0.02	0.92	0.97	4												
		Deep learning	0.95	0.06	0.79	0.99	8	54.50	0.76228	21.00	0.94505	37.50	0.85888	18.50	0.88741	30.00	0.39813	9.00	0.28283
	PPV	Machine learning	0.45	0.16	0.04	0.64	10												
		Manual scoring	0.51	0.16	0.12	0.78	10												
		Proprietary algorithm	0.44	0.15	0.26	0.62	4												
		Deep learning	0.47	0.07	0.30	0.57	8	35.00	0.27304	17.00	0.73327	26.00	0.23699	25.00	0.53946	34.00	0.63344	10.00	0.36768
	NPV	Machine learning	0.96	0.03	0.89	0.99	10												
		Manual scoring	0.95	0.04	0.85	0.98	9												
		Proprietary algorithm	0.94	0.02	0.90	0.96	4												
		Deep learning	0.95	0.02	0.93	0.98	8	56.00	0.39085	29.00	0.22831	50.50	0.37352	24.00	0.41399	35.50	1.00000	11.00	0.46061
	F1	Machine learning	0.28	0.09	0.07	0.37	10												
		Manual scoring	0.49	0.15	0.17	0.66	10												
		Proprietary algorithm	0.43	0.16	0.20	0.61	4												
		Deep learning	0.45	0.11	0.25	0.56	8	18.00	**0.01726**	11.00	0.23976	16.50	**0.04089**	25.00	0.53946	43.00	0.82856	14.00	0.80808
	MCC	Machine learning	0.26	0.07	0.05	0.34	10												
		Manual scoring	0.46	0.14	0.15	0.65	9												
		Proprietary algorithm	0.39	0.16	0.16	0.56	4												
		Deep learning	0.42	0.08	0.24	0.47	7	17.00	**0.02474**	15.00	0.53946	10.50	**0.01910**	24.00	0.41399	33.00	0.91818	9.00	0.41212
N2	ACC	Machine learning	0.82	0.04	0.74	0.87	10	23.50	0.08600	10.00	0.18781	20.00	0.08285	14.00	0.60420	36.50	1.00000	18.50	0.73365

	Manual scoring	0.86	0.05	0.81	0.97	9												
	Proprietary algorithm	0.87	0.06	0.77	0.93	4												
	Deep learning	0.86	0.03	0.79	0.92	8												
κ	Machine learning	0.64	0.08	0.47	0.74	10												
	Manual scoring	0.71	0.09	0.58	0.91	10												
	Proprietary algorithm	0.72	0.10	0.52	0.79	4												
	Deep learning	0.73	0.07	0.59	0.83	7	29.00	0.12122	10.00	0.18781	16.00	0.07024	16.00	0.63536	28.00	0.53620	14.00	1.00000
**SE**	Machine learning	0.79	0.09	0.58	0.89	10												
	Manual scoring	0.85	0.05	0.76	0.94	10												
	Proprietary algorithm	0.83	0.07	0.69	0.91	5												
	Deep learning	0.84	0.06	0.70	0.94	8	29.00	0.12122	19.50	0.53993	27.00	0.27428	27.00	0.85914	40.50	1.00000	18.00	0.83294
**SP**	Machine learning	0.85	0.07	0.71	0.98	10												
	Manual scoring	0.86	0.05	0.80	0.99	10												
	Proprietary algorithm	0.87	0.08	0.73	0.96	4												
	Deep learning	0.88	0.04	0.83	0.97	8	46.00	0.79134	17.00	0.73327	29.00	0.35993	18.00	0.83916	27.00	0.27428	15.00	0.93333
**PPV**	Machine learning	0.81	0.08	0.70	0.94	10												
	Manual scoring	0.82	0.07	0.71	0.95	10												
	Proprietary algorithm	0.79	0.10	0.59	0.85	4												
	Deep learning	0.85	0.06	0.76	0.95	8	44.50	0.70535	19.00	0.94505	27.00	0.27428	21.00	0.94505	23.00	0.14571	9.50	0.30733
**NPV**	Machine learning	0.84	0.05	0.77	0.90	10												
	Manual scoring	0.89	0.05	0.80	0.97	9												
	Proprietary algorithm	0.92	0.03	0.87	0.95	4												
	Deep learning	0.87	0.06	0.74	0.93	8	26.00	0.13091	4.00	**0.02398**	28.00	0.31542	12.00	0.41399	40.00	0.74299	25.00	0.15354
**F1**	Machine learning	0.79	0.06	0.70	0.86	10												
	Manual scoring	0.83	0.05	0.77	0.93	10												
	Proprietary algorithm	0.82	0.06	0.70	0.86	4												
	Deep learning	0.84	0.04	0.78	0.90	8	31.50	0.17346	14.00	0.45355	17.00	**0.04342**	20.00	1.00000	28.00	0.31542	12.00	0.56970

	**MCC**	Machine learning	0.64	0.08	0.47	0.74	10												
		Manual scoring	0.72	0.09	0.61	0.91	9												
		Proprietary algorithm	0.72	0.09	0.54	0.79	4												
		Deep learning	0.73	0.07	0.60	0.83	7	24.50	0.10232	10.00	0.18781	15.00	0.05533	15.50	0.75730	28.00	0.75769	14.00	1.00000
**N3**	**ACC**	Machine learning	0.94	0.02	0.88	0.96	10												
		Manual scoring	0.95	0.03	0.90	0.99	9												
		Proprietary algorithm	0.95	0.02	0.92	0.98	4												
		Deep learning	0.95	0.02	0.93	0.98	8	35.00	0.43794	14.00	0.45355	27.00	0.27428	16.00	0.82517	35.50	1.00000	19.00	0.68283
	**κ**	Machine learning	0.78	0.09	0.61	0.86	10												
		Manual scoring	0.78	0.11	0.41	0.91	10												
		Proprietary algorithm	0.77	0.05	0.68	0.82	4												
		Deep learning	0.77	0.08	0.62	0.85	7	59.00	0.52052	24.00	0.63536	41.00	0.60088	25.00	0.53946	36.00	0.96226	12.00	0.78788
	**SE**	Machine learning	0.84	0.11	0.60	1.00	10												
		Manual scoring	0.80	0.09	0.67	0.98	10												
		Proprietary algorithm	0.76	0.12	0.61	0.93	5												
		Deep learning	0.78	0.08	0.58	0.87	8	60.00	0.47268	33.50	0.32675	48.00	0.51479	33.00	0.37096	40.00	1.00000	16.00	0.62160
	**SP**	Machine learning	0.96	0.02	0.91	0.98	10												
		Manual scoring	0.97	0.02	0.95	0.99	10												
		Proprietary algorithm	0.98	0.00	0.97	0.98	4												
		Deep learning	0.98	0.01	0.95	0.99	8	29.50	0.12970	2.00	**0.01283**	19.00	0.06754	15.00	0.52360	33.50	0.59281	15.00	0.93209
	**PPV**	Machine learning	0.82	0.08	0.59	0.92	10												
		Manual scoring	0.85	0.03	0.80	0.89	10												
		Proprietary algorithm	0.82	0.05	0.75	0.87	4												
		Deep learning	0.81	0.12	0.57	0.95	8	40.00	0.47234	23.00	0.73327	37.00	0.82856	26.00	0.43567	44.50	0.72215	14.00	0.80808
	**NPV**	Machine learning	0.96	0.03	0.89	1.00	10												
		Manual scoring	0.96	0.02	0.93	1.00	9												
		Proprietary algorithm	0.97	0.02	0.93	1.00	4	49.00	0.77496	21.00	0.94357	38.50	0.92913	15.50	0.75730	29.00	0.54142	15.00	0.93333

		Deep learning	0.97	0.02	0.94	0.99	8												
		Machine learning	0.82	0.08	0.66	0.89	10												
	**F1**	Manual scoring	0.82	0.05	0.73	0.91	10												
		Proprietary algorithm	0.79	0.04	0.73	0.83	4												
		Deep learning	0.79	0.08	0.65	0.88	8	54.00	0.79134	26.00	0.45355	53.00	0.27428	26.50	0.39562	47.50	0.53375	15.00	0.93333
		Machine learning	0.79	0.08	0.61	0.86	10												
	**MCC**	Manual scoring	0.80	0.06	0.71	0.91	9												
		Proprietary algorithm	0.77	0.05	0.69	0.82	4												
		Deep learning	0.77	0.08	0.62	0.86	7	49.00	0.77505	25.00	0.53946	41.50	0.55794	25.00	0.33007	33.50	0.87375	13.00	0.92727
		Machine learning	0.91	0.05	0.80	0.96	10												
	**ACC**	Manual scoring	0.96	0.03	0.90	0.99	9												
		Proprietary algorithm	0.96	0.02	0.93	0.98	4												
		Deep learning	0.94	0.02	0.89	0.96	8	16.50	**0.02213**	7.00	0.07678	24.00	0.16823	19.00	0.93986	51.50	0.14867	21.00	0.46061
		Machine learning	0.71	0.14	0.34	0.88	10												
	**κ**	Manual scoring	0.81	0.08	0.69	0.92	10												
		Proprietary algorithm	0.75	0.11	0.61	0.89	4												
		Deep learning	0.79	0.04	0.69	0.83	7	24.00	0.05372	16.00	0.63536	19.00	0.13307	28.00	0.30370	38.00	0.81253	9.00	0.41212
**REM**		Machine learning	0.77	0.15	0.46	0.96	10												
	**SE**	Manual scoring	0.83	0.09	0.67	0.94	10												
		Proprietary algorithm	0.75	0.14	0.53	0.93	5												
		Deep learning	0.85	0.03	0.80	0.88	8	35.00	0.27304	28.00	0.76790	20.00	0.08300	36.00	0.20646	38.50	0.92913	9.00	0.12377
		Machine learning	0.94	0.03	0.88	0.98	10												
	**SP**	Manual scoring	0.98	0.02	0.91	1.00	10												
		Proprietary algorithm	0.97	0.02	0.95	0.99	4												
		Deep learning	0.96	0.02	0.90	0.98	8	13.50	**0.00644**	7.00	0.07678	25.50	0.21281	24.50	0.57076	67.00	**0.01842**	23.00	0.26877
	**PPV**	Machine learning	0.76	0.11	0.47	0.89	10												
		Manual scoring	0.86	0.06	0.72	0.96	10	18.00	**0.01726**	20.00	1.00000	28.50	0.32813	31.00	0.14186	62.00	0.05453	12.00	0.56970

	Proprietary algorithm	0.77	0.10	0.62	0.88	4												
	Deep learning	0.80	0.05	0.70	0.89	8												
	Machine learning	0.95	0.03	0.87	0.98	10												
**NPV**	Manual scoring	0.97	0.02	0.93	1.00	9												
	Proprietary algorithm	0.98	0.01	0.96	0.99	4												
	Deep learning	0.97	0.01	0.95	0.99	8	23.00	0.07905	5.50	**0.04723**	22.50	0.13032	15.50	0.75730	44.50	0.44086	23.00	0.26877
	Machine learning	0.76	0.13	0.47	0.92	10												
**F1**	Manual scoring	0.84	0.06	0.74	0.93	10												
	Proprietary algorithm	0.77	0.10	0.65	0.91	4												
	Deep learning	0.82	0.03	0.76	0.86	8	25.00	0.06402	19.00	0.94505	20.00	0.08314	31.00	0.14186	43.50	0.78970	9.00	0.28283
	Machine learning	0.71	0.14	0.34	0.89	10												
**MCC**	Manual scoring	0.82	0.07	0.70	0.92	9												
	Proprietary algorithm	0.75	0.11	0.61	0.89	4												
	Deep learning	0.79	0.04	0.69	0.83	7	19.00	**0.03717**	14.00	0.45355	19.00	0.13307	26.00	0.26014	37.00	0.60647	9.00	0.41212

ACC: accuracy, κ: Cohen’s Kappa, MCC: Matthew’s Correlation Coefficient, NPV: negative predictive value, PPV: positive predictive value, SE: sensitivity, SP: specificity.

Supplementary Table S11. ***Leave-one-out sensitivity analysis of wEEG performance metrics.*** This table summarizes the results of a leave-one-out analysis to evaluate the influence of individual studies on reported performance metrics across overall and stage-specific evaluations. For each metric, the baseline value reflects the result from the full dataset. "Max Δ" indicates the largest change observed when a single study was removed, and "Max Δ %" expresses this change as a percentage of the baseline. "Max |Δ| > 0.03" indicates whether any individual study's exclusion caused a change greater than ±0.03. The "Most influential study" identifies the study whose exclusion produced the largest absolute change. No metric showed a shift exceeding 0.03, suggesting that the results are robust to the exclusion of any single study.

		Metric	Baseline	Max Δ	Max |Δ| > 0.03	Max Δ %	Most influential study
**Overall**		**Multiclass**					
		**MCC**	0.70	0.008	No	1.184	Mikkelsen et al., 2017
		**κ**	0.68	0.007	No	1.089	Sterr et al., 2018
	**Micro-averaged**	**ACC**	0.78	0.006	No	0.729	Sterr et al., 2018
		**SE**	0.79	0.006	No	0.770	Mikkelsen et al., 2017
		**SP**	0.95	0.002	No	0.162	Mikkelsen et al., 2017
		**PPV**	0.79	0.006	No	0.770	Mikkelsen et al., 2017
		**NPV**	0.95	0.002	No	0.162	Mikkelsen et al., 2017
		**F1**	0.79	0.006	No	0.770	Mikkelsen et al., 2017
	**Macro-averaged**	**ACC**	0.92	0.002	No	0.267	Mikkelsen et al., 2017
		**κ**	0.65	0.008	No	1.302	Mikkelsen et al., 2017
		**SE**	0.71	0.007	No	0.916	Mikkelsen et al., 2017
		**SP**	0.94	0.002	No	0.174	Mikkelsen et al., 2017
		**PPV**	0.72	0.007	No	0.975	Mikkelsen et al., 2017
		**NPV**	0.94	0.002	No	0.182	Mikkelsen et al., 2017
		**F1**	0.71	0.007	No	1.012	Mikkelsen et al., 2017
		**MCC**	0.65	0.008	No	1.289	Mikkelsen et al., 2017
**Wake**		**ACC**	0.94	0.003	No	0.329	Mikkelsen et al., 2017
		**κ**	0.75	0.011	No	1.443	Finan et al., 2016a
		**SE**	0.79	0.012	No	1.484	Finan et al., 2016b
		**SP**	0.96	0.002	No	0.200	Hammour et al., 2024
		**PPV**	0.81	0.011	No	1.298	Finan et al., 2016a
		**NPV**	0.96	0.003	No	0.315	Mikkelsen et al., 2017
		**F1**	0.80	0.011	No	1.331	Finan et al., 2016a
		**MCC**	0.76	0.011	No	1.501	Finan et al., 2016a
**N1**		**ACC**	0.91	0.004	No	0.446	Um et al., 2025
		**κ**	0.31	-0.011	No	3.528	Li et al., 2025b
		**SE**	0.36	-0.011	No	3.058	Um et al., 2025
		**SP**	0.95	0.005	No	0.513	Um et al., 2025
		**PPV**	0.40	-0.012	No	2.938	Seol et al., 2024
		**NPV**	0.95	0.003	No	0.335	Seol et al., 2024
		**F1**	0.35	-0.010	No	2.786	Li et al., 2025b
		**MCC**	0.33	-0.011	No	3.288	Li et al., 2025b
**N2**		**ACC**	0.85	-0.004	No	0.463	Li et al., 2025b
		**κ**	0.68	-0.007	No	1.078	Li et al., 2025b
		**SE**	0.82	0.007	No	0.872	Jørgensen et al., 2023
		**SP**	0.86	0.005	No	0.534	Nakamura et al., 2020
		**PPV**	0.Ravindran et al., 2025[2]				
		**NPV**	0.87	0.004	No	0.496	Um et al., 2025
		**F1**	0.81	0.004	No	0.447	Ravindran et al., 2025
		**MCC**	0.69	-0.007	No	1.064	Li et al., 2025b
**N3**		**ACC**	0.94	0.002	No	0.222	Finan et al., 2016a
		**κ**	0.76	0.011	No	1.490	Oz et al., 2023
		**SE**	0.79	0.006	No	0.809	McMahon et al., 2024
		**SP**	0.97	0.002	No	0.194	Jørgensen et al., 2023
		**PPV**	0.82	0.008	No	0.954	Massie et al., 2025

	**NPV**	0.96	0.002	No	0.235	Finan et al., 2016a
	**F1**	0.80	0.005	No	0.593	Massie et al., 2025
	**MCC**	0.78	0.005	No	0.702	Mikkelsen et al., 2017
**REM**	**ACC**	0.93	0.004	No	0.478	Mikkelsen et al., 2017
	**κ**	0.74	0.013	No	1.746	Mikkelsen et al., 2017
	**SE**	0.79	0.010	No	1.263	Nakamura et al., 2020
	**SP**	0.96	0.003	No	0.264	Mikkelsen et al., 2017
	**PPV**	0.79	0.010	No	1.239	Mikkelsen et al., 2017
	**NPV**	0.96	0.003	No	0.309	Mikkelsen et al., 2017
	**F1**	0.79	0.010	No	1.267	Mikkelsen et al., 2017
	**MCC**	0.75	0.014	No	1.810	Mikkelsen et al., 2017

ACC: accuracy, κ: Cohen's Kappa, MCC: Matthew's Correlation Coefficient, NPV: negative predictive value, PPV: positive predictive value, SE: sensitivity, SP: specificity.

Supplementary Table S12. ***Leave-one-out (LOO) sensitivity analysis for stratified meta-analytic performance estimates.*** For each subgroup (e.g., population type, environment, device characteristics), we conducted a leave-one-out analysis by removing one study at a time and recalculating the pooled performance metric. Only metrics that shifted by more than ±0.03 in absolute terms are shown. "Δ (+−)" denotes the signed change in the pooled value after omitting the most influential study; a negative value indicates that the pooled estimate decreased. "|Δ|" is the absolute change, and "Δ%" shows the relative change as a percentage of the baseline.

Group	Stage	Metric	Baseline	Δ (+−)	|Δ|	Δ%	Study
**Population type**							
**Healthy** (*n* = 24)	No metric in the "Healthy" subgroup exceeded the 0.03 threshold						
**Mixed** (*n* = 7)	**OA macro-averaged**	κ	0.687	-0.034	0.034	5.004	Li et al., 2025b
			0.687	0.030	0.030	4.369	Ravindran et al., 2025
		MCC	0.690	-0.034	0.034	4.912	Li et al., 2025b
	**OA**	Multiclass MCC	0.733	-0.033	0.033	4.538	Li et al., 2025b
	**N1**	κ	0.322	-0.054	0.054	16.748	Li et al., 2025b
		SE	0.370	-0.055	0.055	14.820	Li et al., 2025b
				0.038	0.038	10.315	Ravindran et al., 2025
				0.035	0.035	9.459	Oz et al., 2023
				-0.034	0.034	9.144	Myllymaa et al., 2016
		PPV	0.400	-0.039	0.039	9.737	Li et al., 2025b
		F1	0.367	-0.050	0.050	13.529	Li et al., 2025b
		MCC	0.343	-0.061	0.061	17.658	Li et al., 2025b
				0.037	0.037	10.673	Ravindran et al., 2025
	**N2**	κ	0.712	-0.032	0.032	4.556	Li et al., 2025b
				0.032	0.032	4.486	Ravindran et al., 2025
		PPV	0.805	0.036	0.036	4.479	Ravindran et al., 2025
		MCC	0.738	0.039	0.039	5.251	Ravindran et al., 2025
				-0.034	0.034	4.564	Li et al., 2025b
	**N3**	κ	0.740	0.055	0.055	7.428	Oz et al., 2023
	**REM**	MCC	0.761	0.031	0.031	4.082	Ravindran et al., 2025
**Clinical** (*n* = 5)	**N1**	SE	0.496	0.069	0.069	13.902	McMahon et al., 2024
				-0.033	0.033	6.578	Jørgensen et al., 2020
		PPV	0.542	-0.059	0.059	10.965	Seol et al., 2024
				0.041	0.041	7.479	McMahon et al., 2024
		F1	0.509	0.057	0.057	11.237	McMahon et al., 2024
				-0.036	0.036	7.000	Seol et al., 2024
	**N3**	κ	0.723	0.036	0.036	4.989	Massie et al., 2025
		SE	0.709	0.032	0.032	4.554	McMahon et al., 2024
		PPV	0.792	0.056	0.056	7.093	Massie et al., 2025
		MCC	0.727	0.035	0.035	4.814	Massie et al., 2025
**Environment**							
**Controlled** (*n* = 26)	No metric in the "Controlled" subgroup exceeded the 0.03 threshold						
**Home** (*n* = 10)	**N1**	PPV	0.4037	0.0402	0.0402	9.9551	Mikkelsen et al., 2017
	**REM**	κ	0.6953	0.0394	0.0394	5.6618	Mikkelsen et al., 2017
		SE	0.7296	0.0302	0.0302	4.1357	Nakamura et al., 2020
		PPV	0.7755	0.0335	0.0335	4.3194	Mikkelsen et al., 2017
		F1	0.7483	0.0313	0.0313	4.1769	Mikkelsen et al., 2017
		MCC	0.6977	0.0396	0.0396	5.6806	Mikkelsen et al., 2017
**Commercial/Prototype**							
**Prototype** (*n* = 21)	No metric in the "Prototype" subgroup exceeded the 0.03 threshold						
	**N1**	PPV	0.368	-0.032	0.032	8.609	Seol et al., 2024

**Commercial** (*n* = 15)		MCC	0.311	-0.030	0.030	9.810	Li et al., 2025b
**Electrode placement**							
**Forehead** (*n* = 22)	No metric in the “Forehead” subgroup exceeded the 0.03 threshold						
**Ear** (*n* = 14)	**N1**	PPV	0.433	0.036	0.036	8.209	Mikkelsen et al., 2017
	**REM**	κ	0.719	0.034	0.034	4.778	Mikkelsen et al., 2017
		MCC	0.721	0.035	0.035	4.793	Mikkelsen et al., 2017
**Electrode type**							
**Dry** (*n* = 22)	No metric in the “Dry” subgroup exceeded the 0.03 threshold						
**Wet** (*n* = 14)	**N1**	κ	0.372	0.037	0.037	9.947	Mikkelsen et al., 2017
				0.031	0.031	8.454	Nakamura et al., 2020
		SE	0.402	0.032	0.032	7.984	Nakamura et al., 2020
		PPV	0.471	0.043	0.043	9.109	Mikkelsen et al., 2017
				-0.031	0.031	6.545	Seol et al., 2024
		F1	0.412	0.035	0.035	8.375	Mikkelsen et al., 2017
				0.032	0.032	7.792	Nakamura et al., 2020
		MCC	0.386	0.037	0.037	9.699	Mikkelsen et al., 2017
	**REM**	κ	0.704	0.040	0.040	5.726	Mikkelsen et al., 2017
		PPV	0.788	0.031	0.031	3.983	Mikkelsen et al., 2017
		MCC	0.707	0.041	0.041	5.755	Mikkelsen et al., 2017
**Device scoring**							
**Manual scoring** (*n* = 11)	**Wake**	κ	0.755	0.035	0.035	4.654	Finan et al., 2016b
		SE	0.794	0.044	0.044	5.499	Finan et al., 2016b
		F1	0.803	0.037	0.037	4.582	Finan et al., 2016b
		MCC	0.765	0.040	0.040	5.192	Finan et al., 2016b
	**N1**	SE	0.445	0.032	0.032	7.117	Oz et al., 2023
		PPV	0.444	-0.037	0.037	8.417	Seol et al., 2024
				0.036	0.036	8.082	Finan et al., 2016b
		MCC	0.402	0.032	0.032	7.900	Finan et al., 2016b
				-0.030	0.030	7.579	Li et al., 2025b
	**N3**	κ	0.753	0.038	0.038	5.063	Oz et al., 2023
**Machine learning** (*n* = 10)	**Wake**	κ	0.703	0.036	0.036	5.082	Finan et al., 2016a
		SE	0.762	0.040	0.040	5.229	Finan et al., 2016a
		PPV	0.739	0.033	0.033	4.487	Finan et al., 2016a
		F1	0.745	0.036	0.036	4.819	Finan et al., 2016a
		MCC	0.706	0.036	0.036	5.114	Finan et al., 2016a
	**N1**	PPV	0.327	-0.040	0.040	12.080	Nakamura et al., 2020
				0.036	0.036	10.894	Mikkelsen et al., 2017
				0.032	0.032	9.786	Finan et al., 2016a
	**REM**	κ	0.670	0.041	0.041	6.138	Mikkelsen et al., 2017
		SE	0.723	0.033	0.033	4.576	Nakamura et al., 2020
				0.033	0.033	4.542	Mikkelsen et al., 2017
		PPV	0.740	0.033	0.033	4.494	Mikkelsen et al., 2017
		F1	0.727	0.033	0.033	4.473	Mikkelsen et al., 2017
		MCC	0.672	0.041	0.041	6.160	Mikkelsen et al., 2017
**Deep learning** (*n* = 8)	**N1**	SE	0.413	-0.046	0.046	11.042	Um et al., 2025
				0.035	0.035	8.542	Kwon et al., 2023a
	**N3**	PPV	0.791	0.032	0.032	4.049	Massie et al., 2025
**Proprietary algorithm** (*n* = 5)	**OA**	ACC	0.795	0.035	0.035	4.412	Ravindran et al., 2025
				-0.032	0.032	3.971	Li et al., 2025a
		κ	0.659	0.047	0.047	7.165	Roach et al., 2025
				-0.034	0.034	5.168	Li et al., 2025a

		MCC	0.710	0.039	0.039	5.520	Ravindran et al., 2025
	**OA micro-averaged**	SE	0.795	0.035	0.035	4.412	Ravindran et al., 2025
				-0.032	0.032	3.971	Li et al., 2025a
		PPV	0.795	0.035	0.035	4.412	Ravindran et al., 2025
				-0.032	0.032	3.971	Li et al., 2025a
		F1	0.795	0.035	0.035	4.412	Ravindran et al., 2025
				-0.032	0.032	3.971	Li et al., 2025a
	**OA macro-averaged**	κ	0.662	0.042	0.042	6.294	Ravindran et al., 2025
				-0.034	0.034	5.186	Borges et al., 2024
		SE	0.715	0.034	0.034	4.765	Ravindran et al., 2025
		PPV	0.730	-0.032	0.032	4.325	Borges et al., 2024
		F1	0.717	0.034	0.034	4.676	Ravindran et al., 2025
				-0.031	0.031	4.350	Borges et al., 2024
		MCC	0.666	0.039	0.039	5.896	Ravindran et al., 2025
				-0.033	0.033	5.019	Borges et al., 2024
	**Wake**	κ	0.800	-0.036	0.036	4.440	Li et al., 2025a
		SE	0.779	-0.048	0.048	6.146	Li et al., 2025a
				0.045	0.045	5.735	Roach et al., 2025
		F1	0.859	-0.036	0.036	4.141	Li et al., 2025a
		MCC	0.802	-0.035	0.035	4.323	Li et al., 2025a
	**N1**	κ	0.309	-0.083	0.083	26.861	Borges et al., 2024
				0.055	0.055	17.799	Ravindran et al., 2025
				0.031	0.031	9.924	Levendowski et al., 2017a
		SE	0.318	-0.071	0.071	22.297	Borges et al., 2024
				0.044	0.044	13.922	Ravindran et al., 2025
				0.032	0.032	10.072	Roach et al., 2025
		PPV	0.396	-0.075	0.075	19.057	Borges et al., 2024
				0.045	0.045	11.350	Levendowski et al., 2017a
		F1	0.362	-0.083	0.083	22.981	Borges et al., 2024
				0.054	0.054	15.022	Ravindran et al., 2025
		MCC	0.314	-0.082	0.082	25.997	Borges et al., 2024
				0.051	0.051	16.321	Ravindran et al., 2025
				0.032	0.032	10.048	Levendowski et al., 2017a
	**N2**	ACC	0.862	0.031	0.031	3.618	Ravindran et al., 2025
		κ	0.700	0.060	0.060	8.563	Ravindran et al., 2025
				-0.031	0.031	4.490	Li et al., 2025a
		SE	0.824	0.034	0.034	4.066	Roach et al., 2025
		SP	0.860	0.044	0.044	5.155	Ravindran et al., 2025
				-0.033	0.033	3.798	Li et al., 2025a
		PPV	0.778	0.063	0.063	8.081	Ravindran et al., 2025
		F1	0.811	0.038	0.038	4.627	Ravindran et al., 2025
		MCC	0.707	0.054	0.054	7.667	Ravindran et al., 2025
	**N3**	SE	0.744	-0.047	0.047	6.284	Li et al., 2025a
				0.034	0.034	4.503	Roach et al., 2025
	**REM**	κ	0.737	-0.053	0.053	7.128	Borges et al., 2024
				0.044	0.044	5.952	Ravindran et al., 2025
		SE	0.721	-0.053	0.053	7.342	Borges et al., 2024
				0.048	0.048	6.628	Roach et al., 2025
		PPV	0.760	0.048	0.048	6.360	Ravindran et al., 2025
				-0.041	0.041	5.395	Borges et al., 2024
		F1	0.761	-0.049	0.049	6.452	Borges et al., 2024

				0.038	0.038	5.028	Ravindran et al., 2025
		MCC	0.739	-0.052	0.052	7.019	Borges et al., 2024
			0.739	0.044	0.044	5.981	Ravindran et al., 2025

ACC: accuracy, κ: Cohen’s Kappa, MCC: Matthew’s Correlation Coefficient, NPV: negative predictive value, PPV: positive predictive value, SE: sensitivity, SP: specificity.

PRISMA 2020 Checklist

Section and Topic	Item #	Checklist item	Location where item is reported
**TITLE**			
Title	1	Identify the report as a systematic review.	Title page
**ABSTRACT**			
Abstract	2	See the PRISMA 2020 for Abstracts checklist.	Abstract
**INTRODUCTION**			
Rationale	3	Describe the rationale for the review in the context of existing knowledge.	Introduction
Objectives	4	Provide an explicit statement of the objective(s) or question(s) the review addresses.	Introduction
**METHODS**			
Eligibility criteria	5	Specify the inclusion and exclusion criteria for the review and how studies were grouped for the syntheses.	Methods (Study selection)
Information sources	6	Specify all databases, registers, websites, organisations, reference lists and other sources searched or consulted to identify studies. Specify the date when each source was last searched or consulted.	Methods (Search strategy)
Search strategy	7	Present the full search strategies for all databases, registers and websites, including any filters and limits used.	Methods (Search strategy)
Selection process	8	Specify the methods used to decide whether a study met the inclusion criteria of the review, including how many reviewers screened each record and each report retrieved, whether they worked independently, and if applicable, details of automation tools used in the process.	Methods (Study selection)
Data collection process	9	Specify the methods used to collect data from reports, including how many reviewers collected data from each report, whether they worked independently, any processes for obtaining or confirming data from study investigators, and if applicable, details of automation tools used in the process.	Methods (Data extraction)
Data items	10a	List and define all outcomes for which data were sought. Specify whether all results that were compatible with each outcome domain in each study were sought (e.g. for all measures, time points, analyses), and if not, the methods used to decide which results to collect.	Methods (Data extraction)
	10b	List and define all other variables for which data were sought (e.g. participant and intervention characteristics, funding sources). Describe any assumptions made about any missing or unclear information.	Methods (Data extraction)
Study risk of bias assessment	11	Specify the methods used to assess risk of bias in the included studies, including details of the tool(s) used, how many reviewers assessed each study and whether they worked independently, and if applicable, details of automation tools used in the process.	Methods (Data extraction)
Effect measures	12	Specify for each outcome the effect measure(s) (e.g. risk ratio, mean difference) used in the synthesis or presentation of results.	Methods (Data analysis)
Synthesis methods	13a	Describe the processes used to decide which studies were eligible for each synthesis (e.g. tabulating the study intervention characteristics and comparing against the planned groups for each synthesis (item #5)).	Methods (Study selection)
	13b	Describe any methods required to prepare the data for presentation or synthesis, such as handling of missing summary statistics, or data conversions.	Methods (Data extraction and Data analysis)
	13c	Describe any methods used to tabulate or visually display results of individual studies and syntheses.	Methods (Data extraction), Results (Tables 1-3)
	13d	Describe any methods used to synthesize results and provide a rationale for the choice(s). If meta-analysis was performed, describe the model(s), method(s) to identify the presence and extent of statistical heterogeneity, and software package(s) used.	Methods (Data analysis)
	13e	Describe any methods used to explore possible causes of heterogeneity among study results (e.g. subgroup analysis, meta-regression).	Methods (Data

PRISMA 2020 Checklist

Section and Topic	Item #	Checklist item	Location where item is reported
			analysis)
	13f	Describe any sensitivity analyses conducted to assess robustness of the synthesized results.	Methods (Data analysis)
Reporting bias assessment	14	Describe any methods used to assess risk of bias due to missing results in a synthesis (arising from reporting biases).	N/R
Certainty assessment	15	Describe any methods used to assess certainty (or confidence) in the body of evidence for an outcome.	N/R
**RESULTS**			
Study selection	16a	Describe the results of the search and selection process, from the number of records identified in the search to the number of studies included in the review, ideally using a flow diagram.	Methods (Search strategy, Study selection, Figure 8)
	16b	Cite studies that might appear to meet the inclusion criteria, but which were excluded, and explain why they were excluded.	Methods (Search strategy, Study selection, Figure 8)
Study characteristics	17	Cite each included study and present its characteristics.	Results (Tables 1, 2 and 3)
Risk of bias in studies	18	Present assessments of risk of bias for each included study.	N/R
Results of individual studies	19	For all outcomes, present, for each study: (a) summary statistics for each group (where appropriate) and (b) an effect estimate and its precision (e.g. confidence/credible interval), ideally using structured tables or plots.	Results (Analysis of validation studies, Influence of methodological variability on wEEG performance, Tables 1-3)
Results of syntheses	20a	For each synthesis, briefly summarise the characteristics and risk of bias among contributing studies.	Results (Analysis of validation studies, Influence of methodological variability on wEEG performance, Figures 4-7, Supplementary Figures S1-S8, Supplementary Tables S1-S10)
	20b	Present results of all statistical syntheses conducted. If meta-analysis was done, present for each the summary estimate and its precision (e.g. confidence/credible interval) and measures of statistical heterogeneity. If comparing groups, describe the direction of the effect.	Results (Analysis of validation studies, Influence

PRISMA

PRISMA 2020 Checklist

Section and Topic	Item #	Checklist item	Location where item is reported
			of methodological variability on wEEG performance, Figures 4-7, Supplementary Figures S1-S8, Supplementary Tables S1-S10)
	20c	Present results of all investigations of possible causes of heterogeneity among study results.	Results (Analysis of validation studies, Influence of methodological variability on wEEG performance, Figures 4-7, Supplementary Figures S1-S8, Supplementary Tables S1-S10)
	20d	Present results of all sensitivity analyses conducted to assess the robustness of the synthesized results.	Results (Sensitivity analysis, Supplementary Figure S9, Supplementary Tables S11 and S12)
Reporting biases	21	Present assessments of risk of bias due to missing results (arising from reporting biases) for each synthesis assessed.	N/R (not formally assessed, limitations discussed in Discussion)
Certainty of evidence	22	Present assessments of certainty (or confidence) in the body of evidence for each outcome assessed.	N/R (not formally assessed, limitations discussed in Discussion)
**DISCUSSION**			
Discussion	23a	Provide a general interpretation of the results in the context of other evidence.	Discussion (paragraph 1-3)
	23b	Discuss any limitations of the evidence included in the review.	Discussion (paragraph 8)

PRISMA 2020 Checklist

Section and Topic	Item #	Checklist item	Location where item is reported
	23c	Discuss any limitations of the review processes used.	Discussion (paragraph 8)
	23d	Discuss implications of the results for practice, policy, and future research.	Discussio (paragraph 9)
**OTHER INFORMATION**			
Registration and protocol	24a	Provide registration information for the review, including register name and registration number, or state that the review was not registered.	N/A
	24b	Indicate where the review protocol can be accessed, or state that a protocol was not prepared.	N/A
	24c	Describe and explain any amendments to information provided at registration or in the protocol.	N/A
Support	25	Describe sources of financial or non-financial support for the review, and the role of the funders or sponsors in the review.	Acknowledgments
Competing interests	26	Declare any competing interests of review authors.	Competing interests
Availability of data, code and other materials	27	Report which of the following are publicly available and where they can be found: template data collection forms; data extracted from included studies; data used for all analyses; analytic code; any other materials used in the review.	Data availability

From: Page MJ, McKenzie JE, Bossuyt PM, Boutron I, Hoffmann TC, Mulrow CD, et al. The PRISMA 2020 statement: an updated guideline for reporting systematic reviews. BMJ 2021;372:n71. doi: 10.1136/bmj.n71. This work is licensed under CC BY 4.0. To view a copy of this license, visit https://creativecommons.org/licenses/by/4.0/

References

1. Li, X. *et al.* Exploring the potential of a new wearable sleep monitoring device for clinical application. *Biomed Signal Process Control* **99**, 106856 (2025).
2. G Ravindran, K. K. *et al.* Evaluation of Dreem headband for sleep staging and EEG spectral analysis in people living with Alzheimer's and older adults. *Sleep* (2025) doi:10.1093/sleep/zsaf122.
3. Seol, J. *et al.* Validation of sleep-staging accuracy for an in-home sleep electroencephalography device compared with simultaneous polysomnography in patients with obstructive sleep apnea. *Sci Rep* **14**, 3533 (2024).
4. Rusanen, M. *et al.* Generalizable deep learning-based sleep staging approach for ambulatory textile electrode headband recordings. *IEEE J Biomed Health Inform* 1–12 (2023) doi:10.1109/JBHI.2023.3240437.
5. Casciola, A. A. *et al.* A Deep Learning Strategy for Automatic Sleep Staging Based on Two-Channel EEG Headband Data. *Sensors* **21**, 3316 (2021).
6. Arnal, P. J. *et al.* The Dreem Headband compared to polysomnography for electroencephalographic signal acquisition and sleep staging. *Sleep* **43**, (2020).
7. Lin, C.-T. *et al.* Forehead EEG in Support of Future Feasible Personal Healthcare Solutions: Sleep Management, Headache Prevention, and Depression Treatment. *IEEE Access* **5**, 10612–10621 (2017).
8. Levendowski, D. J. *et al.* The accuracy, night-to-night variability, and stability of frontopolar sleep electroencephalography biomarkers. *Journal of Clinical Sleep Medicine* **13**, 791–803 (2017).
9. Finan, P. H. *et al.* Validation of a wireless, self-application, ambulatory electroencephalographic sleep monitoring device in healthy volunteers. *Journal of Clinical Sleep Medicine* **12**, 1443–1451 (2016).
10. Liang, S.-F. *et al.* Development of an EOG-based automatic sleep-monitoring eye mask. *IEEE Trans Instrum Meas* **64**, 2977–2985 (2015).
11. Massie, F., Vits, S., Verbraecken, J. & Bergmann, J. The evaluation of a novel single-lead biopotential device for home sleep testing. *Sleep* **48**, (2025).
12. Um, H.-K. *et al.* Mobile sleep stage analysis using multichannel wearable devices integrated with stretchable transparent electrodes. *ACS Sens* (2025) doi:10.1021/acssensors.4c03602.
13. Kwon, S. *et al.* At-home wireless sleep monitoring patches for the clinical assessment of sleep quality and sleep apnea. *Sci Adv* **9**, (2023).
14. Matsumori, S. *et al.* HARU sleep: a deep learning-based sleep scoring system with wearable sheet-type frontal EEG sensors. *IEEE Access* **10**, 13624–13632 (2022).
15. Myllymaa, S. *et al.* Assessment of the suitability of using a forehead EEG electrode set and chin EMG electrodes for sleep staging in polysomnography. *J Sleep Res* **25**, 636–645 (2016).
16. Borges, D. F. *et al.* A custom-built single-channel in-ear electroencephalography sensor for sleep phase detection: an interdependent solution for at-home sleep studies. *J Sleep Res* **34**, e14368 (2025).
17. Hammour, G. *et al.* From scalp to ear-EEG: A generalizable transfer learning model for automatic sleep scoring in older people. *IEEE J Transl Eng Health Med* **12**, 448–456 (2024).
18. Borup, K., Kidmose, P., Phan, H. & Mikkelsen, K. Automatic sleep scoring using patient-specific ensemble models and knowledge distillation for ear-EEG data. *Biomed Signal Process Control* **81**, 104496 (2023).

19. Tabar, Y. R. *et al.* At-home sleep monitoring using generic ear-EEG. *Front Neurosci* **17**, (2023).
20. Jørgensen, S. D. *et al.* Long-term ear-EEG monitoring of sleep – A case study during shift work. *J Sleep Res* (2023) doi:10.1111/jsr.13853.
21. Kjaer, T. W., Rank, M. L., Hemmsen, M. C., Kidmose, P. & Mikkelsen, K. Repeated automatic sleep scoring based on ear-EEG is a valuable alternative to manually scored polysomnography. *PLOS Digital Health* **1**, e0000134 (2022).
22. Jørgensen, S. D., Zibrandtsen, I. C. & Kjaer, T. W. Ear-EEG-based sleep scoring in epilepsy: A comparison with scalp-EEG. *J Sleep Res* **29**, (2020).
23. Nakamura, T., Alqurashi, Y. D., Morrell, M. J. & Mandic, D. P. Hearables: automatic overnight sleep monitoring with standardized in-ear EEG sensor. *IEEE Trans Biomed Eng* **67**, 203–212 (2020).
24. Mikkelsen, K. B. *et al.* Accurate whole-night sleep monitoring with dry-contact ear-EEG. *Sci Rep* **9**, 16824 (2019).
25. Mikkelsen, K. B., Villadsen, D. B., Otto, M. & Kidmose, P. Automatic sleep staging using ear-EEG. *Biomed Eng Online* **16**, 111 (2017).
26. da Silva Souto, C. F., Pätzold, W., Paul, M., Debener, S. & Wolf, K. I. Pre-gelled electrode grid for self-applied EEG sleep monitoring at home. *Front Neurosci* **16**, (2022).
27. da Silva Souto, C. F. *et al.* Flex-printed ear-EEG sensors for adequate sleep staging at home. *Front Digit Health* **3**, (2021).
28. Chen, X. *et al.* Validation of a wearable forehead sleep recorder against polysomnography in sleep staging and desaturation events in a clinical sample. *Journal of Clinical Sleep Medicine* **19**, 711–718 (2023).
29. Markwald, R. R., Bessman, S. C., Reini, S. A. & Drummond, S. P. A. Performance of a Portable Sleep Monitoring Device in Individuals with High Versus Low Sleep Efficiency. *Journal of Clinical Sleep Medicine* **12**, 95–103 (2016).
30. Rostaminia, S., Homayounfar, S. Z., Kiaghadi, A., Andrew, T. & Ganesan, D. PhyMask: Robust sensing of brain activity and physiological signals during sleep with an all-textile eye mask. *ACM Trans Comput Healthc* **3**, 1–35 (2022).
31. Hsieh, T.-H., Liu, M.-H., Kuo, C.-E., Wang, Y.-H. & Liang, S.-F. Home-use and real-time sleep-staging system based on eye masks and mobile devices with a deep learning model. *J Med Biol Eng* (2021) doi:10.1007/s40846-021-00649-5.
32. Kaplan, R., Wang, Y., Loparo, K. & Kelly, M. Evaluation of an automated single-channel sleep staging algorithm. *Nat Sci Sleep* 101 (2015) doi:10.2147/NSS.S77888.
33. Nakamura, T., Goverdovsky, V., Morrell, M. J. & Mandic, D. P. Automatic sleep monitoring using ear-EEG. *IEEE J Transl Eng Health Med* **5**, 1–8 (2017).
